# The biosynthetic pathway of potato solanidanes diverged from that of spirosolanes due to evolution of a dioxygenase

**DOI:** 10.1038/s41467-021-21546-0

**Published:** 2021-02-26

**Authors:** Ryota Akiyama, Bunta Watanabe, Masaru Nakayasu, Hyoung Jae Lee, Junpei Kato, Naoyuki Umemoto, Toshiya Muranaka, Kazuki Saito, Yukihiro Sugimoto, Masaharu Mizutani

**Affiliations:** 1 Graduate School of Agricultural Science, Kobe University, Hyogo, Japan; 2 Institute for Chemical Research, Kyoto University, Uji, Kyoto Japan; 3 RIKEN Center for Sustainable Resource Science, Yokohama, Kanagawa Japan; 4 Department of Biotechnology, Graduate School of Engineering, Osaka University, Osaka, Japan; 5 Graduate School of Pharmaceutical Sciences, Chiba University, Chiba, Japan; 6 Present Address: Research Institute for Sustainable Humanosphere, Kyoto University, Uji, Kyoto Japan

**Keywords:** Oxidoreductases, Secondary metabolism

## Abstract

Potato (*Solanum tuberosum*), a worldwide major food crop, produces the toxic, bitter tasting solanidane glycoalkaloids α-solanine and α-chaconine. Controlling levels of glycoalkaloids is an important focus on potato breeding. Tomato (*Solanum lycopersicum*) contains a bitter spirosolane glycoalkaloid, α-tomatine. These glycoalkaloids are biosynthesized from cholesterol via a partly common pathway, although the mechanisms giving rise to the structural differences between solanidane and spirosolane remained elusive. Here we identify a 2-oxoglutarate dependent dioxygenase, designated as DPS (Dioxygenase for Potato Solanidane synthesis), that is a key enzyme for solanidane glycoalkaloid biosynthesis in potato. DPS catalyzes the ring-rearrangement from spirosolane to solanidane via C-16 hydroxylation. Evolutionary divergence of spirosolane-metabolizing dioxygenases contributes to the emergence of toxic solanidane glycoalkaloids in potato and the chemical diversity in Solanaceae.

## Abstract (web summary)

One goal of potato breeding is to reduce the accumulation of toxic solanidane glycoalkaloids. Here the authors show that potato DPS, a 2-oxoglutarate dependent dioxygenase, catalyzes ring rearrangement of a biosynthetic precursor to differentiate solanidanes from spirosolanes that are found in other solanaceous plants.

## Introduction

Plants produce a vast array of specialized metabolites, many of which play essential roles in plant adaptation to environment and contribute to human health^1^. Steroidal glycoalkaloids (SGAs), a class of specialized metabolites, are typically found in the plant family Solanaceae. While they serve as chemical protectant against plant pathogens and herbivores^2,3^, some are notorious as antinutritional substance exerting negative effect on quality and marketability of *Solanum* (Family: Solanaceae) staple food crops, such as potato (*Solanum tuberosum*), tomato (*S. lycopersicum*), and eggplant (*S. melongena*)^4^. Therefore, controlling the SGA level is one of important targets for breeding of Solanaceae species.

**Fig. 1:**
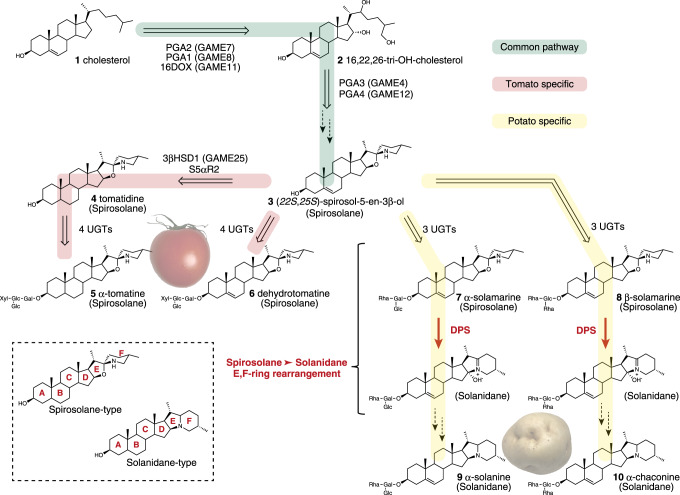
Biosynthesis of solanidane and spirosolane glycoalkaloids in potato and tomato. Common steps between potato and tomato are shaded green. Steps specific to tomato or potato are indicated by red or yellow shade, respectively. White and dashed black arrows represent characterized and uncharacterized reactions, respectively. The reaction step characterized in this work, by DPS (Dioxygenase for Potato Solanidane synthesis), is shown in red arrow. PGA POTATO GLYCOALKALOID BIOSYNTHESIS, GAME GLYCOALKALOID METABOLISM, 16DOX 22,26-hydroxycholesterol 16α-hydroxylase, 3βHSD1 3β-hydroxysteroid dehydrogenases 1, S5αR2 steroid 5α-reductase 2, UGT uridine diphosphate-dependent glycosyltransferases, Glc glucose, Gal galactose, Rha rhamnose, Xyl xylose. Photos of tomato and potato were taken by Ryota Akiyama, Graduate School of Agricultural Science, Kobe University.

SGAs consist of two structural components; the aglycone unit composed of nitrogen containing C_27_ steroid derived from cholesterol and oligosaccharide attached to the hydroxy group at C-3^3,5^. Based on the skeletal structure of the aglycone, SGAs can be divided into two general classes, solanidane or spirosolane (Fig. 1)^3,6^. Minor structural variations of these two ring types such as C-5 saturation/unsaturation or isomerization at C-22, in combination with various sugar moieties, generate the enormous structural diversity of SGAs^7,8^. In addition, their chemical structures reflect their biological activities, for example, toxicity to animals, anti-cancer properties, and anti-microbial activities^3,9^. Most representatives of solanidane glycoalkaloids are potato toxins, α-solanine **9** and α-chaconine **10**, that comprise upward of 90% of the total SGAs in cultivated potatoes^10,11^. Additionally, more than 50 different SGAs, including spirosolane glycoalkaloids, have been identified in a variety of wild potato species and cultivars^11,12^. Tomato and eggplant, on the other hands, contain only spirosolane glycoalkaloids^3,13^. In tomato, α-tomatine **5** and dehydrotomatine **6** are predominant in green tissues, while the non-bitter SGA esculeoside A is the major component in the red mature fruits^14,15^. α-Solasonine and α-solamargine are the two major spirosolane glycoalkaloids produced in eggplant^16^.

SGA biosynthesis can be divided into two main parts: aglycone formation and glycosylation. Recent research in potato and tomato identified several SGA biosynthetic genes involved in aglycone formation. Three cytochrome P450 monooxygenases (CYPs) named as PGA2 (GAME7), PGA1 (GAME8), and PGA3 (GAME4) have been found to be involved in hydroxylation of cholesterol **1** at C-22 and C-26 and oxygenation at C-26, respectively^17,18^. A 2-oxoglutarate-dependent dioxygenase (DOX) named as 16DOX (GAME11), and an aminotransferase were reported to be required for the C-16α-hydroxylation and C-26 amination during SGA biosynthesis^18,19^. These enzymes and functions are common to potato and tomato, suggesting that they are involved in the biosynthetic steps common to solanidanes and spirosolanes (Fig. 1). In addition, several uridine diphosphate-dependent glycosyltransferases (UGTs) involved in the glycosylation steps of SGA biosynthesis have been identified in potato and tomato^20–24^. However, the steps and enzymes involved in the metabolic branch point between solanidane-skeleton and spirosolane-skeleton formation remains an unsolved mystery.

In our efforts to elucidate the missing steps in SGAs biosynthesis, we focused on SGA biosynthesis in potato. Here, we find that solanidane glycoalkaloids are biosynthesized from spirosolane glycoalkaloids via ring rearrangement (Fig. 1). We discover DPS, a member of the DOX superfamily, is a key enzyme required for solanidane biosynthesis. DPS in vitro exhibits a novel activity converting the spirosolane-skeleton to a solanidane-skeleton (Fig. 1). Furthermore, studies of the genome organization of DPS homologs in a number of species from the *Solanum* genus reveal the evolutionary origin of this transformation. These results offer insights into the evolution of chemical diversity of SGAs in *Solanum* species.

## Results

### Discovery of the biosynthetic pathway of solanidane glycoalkaloids

**Fig. 2:**
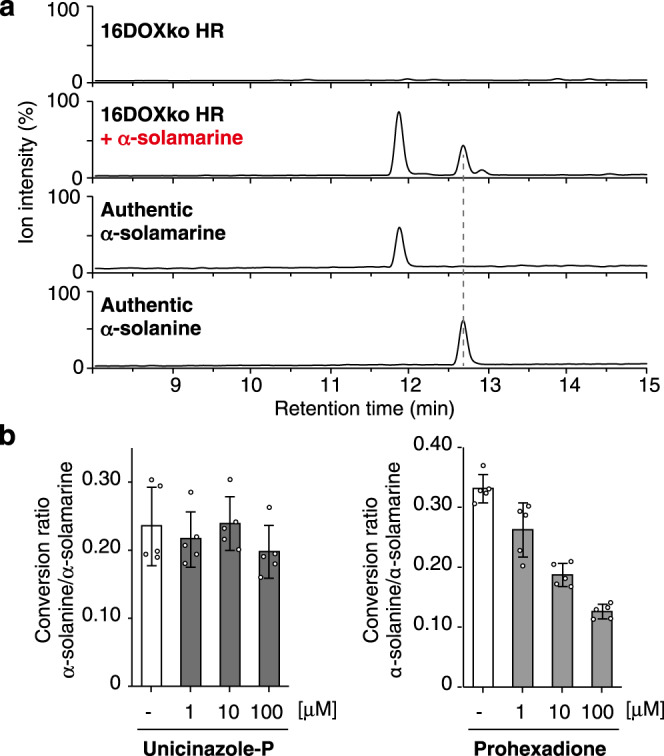
Bioconversion of the solanidane-skeleton to the spirosolane-skeleton in potato hairy roots. **a** LC–MS detection of conversion from administered α-solamarine to α-solanine in 16DOX-disrupted potato hairy roots (16DOXko HR). Total ion current (TIC) chromatogram obtained in positive ionization mode with a full-scan range of 350–1250*m/z* are shown. Production of α-solanine was confirmed by comparison with the authentic standard of α-solanine in terms of retention time and mass spectrum provided in Supplementary Fig. 1. **b** Inhibition of the bioconversion of α-solamarine to α-solanine by uniconazole-P or prohexadione. After administering α-solamarine for 3 days in the presence of uniconazole-P or prohexadione, the SGAs accumulated in 16DOXko HR were extracted and quantified using LC–MS. Bars represent the ratio of α-solamarine to α-solanine accumulation in hairy roots. Error bars represent ± standard deviation of mean (*n* = 5).

Most cultivated potatoes mostly contain only solanidane glycoalkaloids (α-solanine and α-chaconine), while some wild potato species and a few cultivars accumulate spirosolane glycoalkaloids, α-solamarine **7** and β-solamarine **8**, which share the same sugar moiety with α-solanine **9** and α-chaconine **10**, respectively^12,25^. We speculated that modification of the spirosolane-skeleton would form the solanidane-skeleton in potato. To explore this hypothesis, we conducted feeding experiments using SGA-deficient potato hairy roots (16DOXko HR), in which the SGA biosynthetic gene, *St16DOX*, was disrupted^26^. Firstly, the administration of α-solamarine **7** into 16DOXko HR resulted in restoration of the accumulation of α-solanine **9** in 16DOXko HR (Fig. 2a and Supplementary Fig. 1). Similar results were obtained from the feeding experiments with β-solamarine **8**, in which the accumulation of α-chaconine **10** was detected (Supplementary Fig. 2). When feeding with (*22S*,*25S*)-spirosol-5-en-3β-ol **3**, which is an aglycone of α-solamarine **7** and β-solamarine **8**, the accumulation of α-solanine **9**, α-chaconine **10**, and uncharacterized minor SGAs was confirmed (Supplementary Fig. 3). Additionally, the conversion of α-tomatine **6** to its corresponding solanidane, demissine **11**, was confirmed with retention of the lycotetraose attached at the hydroxy group at C-3 (Supplementary Fig. 4). Furthermore, addition of the DOX inhibitor prohexadione during the α-solamarine **7** feeding assay caused the suppression of the conversion of α-solamarine to α-solanine **9** in a dose-dependent manner, whereas uniconazole-P, a CYP inhibitor, had little effects on the bioconversion (Fig. 2b). These data suggest that solanidane glycoalkaloids (i.e., α-solanine **9** and α-chaconine **10**) are biosynthesized from spirosolane glycoalkaloids in potato and that a DOX family enzyme is involved in the conversion reactions.

### Selection of candidate *DOXs* responsible for solanidane biosynthesis

The DOX superfamily is one of the largest enzyme superfamily in plant kingdom and associated with the oxygenation reactions of various specialized metabolites^27^. Recently, involvement of DOXs in SGA biosynthesis and modification was reported^14,15,18,19^. In our previous study, *St16DOX*, which showed the highest fragments per kilobase of exon per million mapped fragments (FPKM) value in tuber sprout among the 256 DOX transcripts in RNA-Seq Gene Expression Data obtained from Spud DB (http://solanaceae.plantbiology.msu.edu/), was identified to encode the steroid 16α-hydroxylase^19^. In this study, we focused on the gene *Sotub01g007130*, which showed the second highest FPKM value in the tuber sprouts among the potato *DOXs* (Supplementary Table 1). Quantitative reverse transcription PCR revealed that *Sotub01g007130* shared a similar expression pattern with previously identified SGA biosynthetic genes^19^ (Supplementary Fig. 5), and we henceforth named *Sotub01g007130* as *DPS* (dioxygenase for potato solanidane synthesis). Many SGA biosynthetic genes were reported to be clustered on chromosomes 7 and 12 in potato and tomato^18^, while *DPS* is positioned on chromosome 1. We found that *DPS* exists in a gene cluster (spanning about 160 kbp) with seven *DOXs* designated as *St7070*, *St7080*, *St7090*, *St7100*, *St7110*, *St7120*, and *St7150*, respectively. *St7090*, *St7100*, *St7120*, and *St7150* appeared to encode truncated proteins lacking canonical motifs conserved among the DOX superfamily^27^. Among these clustered *DOXs*, *St7070*, *St7100*, and *St7110* also showed high FPKM values in potato tuber sprouts (Supplementary Table 1). We successfully amplified the full-length open-reading frame (ORF) sequences of *DPS* and *St7070* using the cDNAs derived from tuber sprouts of potato. DPS shares 85.8% amino acid sequence identity to St7070 and a shorter C-terminus of 36-amino acid residues than St7070.

### Characterization of *DPS*-silenced transgenic potato plants

**Fig. 3:**
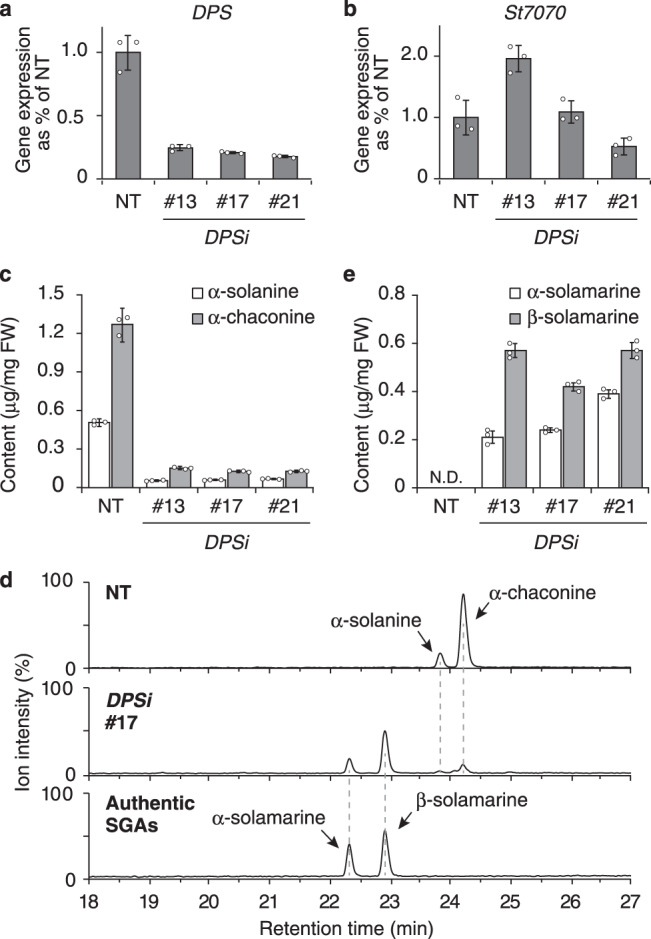
Silencing of *DPS* in potato plants. Real-time quantitative RT-PCR analysis of *DPS* (**a**) and *St7070* (**b**) gene transcript levels in non-transformed and *DPS*-silenced (*DPSi*) potato plants. **c** Quantification of solanidane SGAs accumulated in non-transformed and *DPSi* plants using LC–MS. **d** SGA profiles determined by LC–MS. Total ion current chromatogram obtained in positive ionization mode with a full-scan range of 350–1250*m/z* are shown. Accumulation of α-solamarine and β-solamarine was confirmed by comparison with authentic standards in terms of retention time and mass spectrum provided in Supplementary Fig. 7. **e** Quantification of spirosolane SGAs accumulated in non-transformed and *DPSi* potato plants. Error bars represent ± standard deviation of mean (*n* = 3). FW fresh weight, NT non-transformed control plant, ND not detected, #13, #17, #21 independent transgenic lines.

To investigate the contribution of *DPS* to solanidane biosynthesis, we generated *DPS*-silenced transgenic potato plants (i.e., *DPSi* lines) by using RNA-interference (RNAi) vector containing a stem-loop partial fragment of *DPS* cDNA (Supplementary Fig. 6). *DPSi* plants showed ~80% reduction in *DPS* transcript levels relative to non-transformed (NT) plants (Fig. 3a), and no off-target silencing effect on *St7070* was observed (Fig. 3b). The levels of solanidane glycoalkaloids (α-solanine **9** and α-chaconine **10**) in the leaves of in vitro-grown *DPSi* plants were reduced by ∼90% compared with those of the NT plants (Fig. 3c), suggesting that *DPS* is involved in solanidane biosynthesis in potato. Analysis of endogenous metabolites accumulated in *DPSi* plants detected two new peaks, of which the retention time (Rt) and mass spectrum matched with those of the authentic compounds, α-solamarine **7** and β-solamarine **8** (Fig. 3d and Supplementary Fig. 7). The amounts of α-solamarine **7** and β-solamarine **8** accumulated in *DPSi* plants were comparable to those of α-solanine and α-chaconine in NT potato plants, respectively (Fig. 3e). These results implicate *DPS* as a key gene in the conversion from spirosolanes to solanidanes in potato.

### Characterization of DPS function

**Fig. 4:**
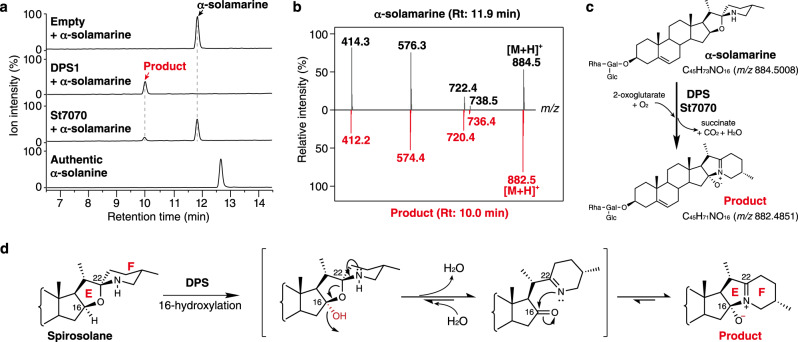
The enzymatic activity of DPS and St7070. **a** LC–MS analysis of the reaction products from the recombinant DPS and St7070 proteins with α-solamarine as a substrate. Total ion current (TIC) chromatogram obtained in positive ionization mode with a full-scan range of 350–1250*m/z* are shown. The negative control reaction was performed using a purified protein fraction from *E. coli* cells transformed with an empty pCold ProS2 vector. **b** Mass spectrum of the peaks at a retention time of 11.9 min (substrate, α-solamarine) and 10.0 min (reaction product). MS fragmentation pathways are shown in Supplementary Fig. 9. **c** Enzymatic reaction DPS or St7070. **d** Putative reaction mechanism of DPS or St7070.

Recombinant His-tagged protein of DPS was prepared with a bacterial expression system in *Escherichia coli* and purified by cobalt-affinity chromatography (Supplementary Fig. 8). The purified DPS was assayed with several spirosolanes as substrates, and the reaction products were analyzed using liquid chromatography–mass spectrometry (LC–MS). DPS metabolized α-solamarine **7** to form an unknown product, of which the retention time and the mass fragment pattern was not identical to α-solanine **9** (Fig. 4a, b). The product gave a parental ion mass at *m*/*z* 882.5 [M + H]^+^ which is two mass smaller than that of the substrate α-solamarine: *m*/*z* 884.5 [M + H]^+^ (Fig. 4b and Supplementary Fig. 9). Negative control reactions using either denatured DPS, lacking cofactors, or containing ethylenediaminetetraacetic acid (EDTA) as an Fe chelator failed to yield any products (Supplementary Fig. 10). The other spirosolane glycoalkaloids (β-solamarine **8** and α-tomatine **6**) and the spirosolane aglycones ((*22S*,*25S*)-spirosol-5-en-3β-ol **3** and tomatidine **4**) were also accepted as substrates, and the mass decrease of 2*m*/*z* was detected for each product (Supplementary Figs. 11–15). On the other hand, DPS is not active with solanidanes as substrates (Supplementary Fig. 16).

To identify the nature of the DPS reaction, we performed high-resolution mass spectrometry (HRMS) (Supplementary Fig. 17) and nuclear magnetic resonance (NMR) (Supplementary Data 1) analyses of the DPS enzymatic product obtained by reacting with α-tomatine **6**, which is commercially available in enough quantities for structural analysis. The structure of the DPS reaction product was determined as shown in Supplementary Fig. 18 (Supplementary Fig. 19, Supplementary Tables 2, 3). The solanidane-skeleton scaffold is formed, and an iminium ion structure is constructed between C-16, the nitrogen atom, and C-22. Thus, DPS seems to catalyze hydroxylation at C-16α of the spirosolane-skeleton to form a hemiacetal that then undergoes condensation with the secondary amine of the piperidine F ring to form the tertiary amine of solanidane, and hence, this enzymatic transformation by DPS is quite unusual (Fig. 4c). To gain insight into the reaction mechanism of dehydration and E/F ring rearrangement by DPS, we investigated the origin of the oxygen atom of the oxy ion in the DPS enzymatic reaction product by ^18^O_2_-labeling experiments. DOXs typically catalyze oxygenation/hydroxylation reactions, and DOX-dependent reactions in the presence of ^18^O_2_ result in the incorporation of ^18^O atom into the product. Accordingly, when the enzyme assay with Sl23DOX, which catalyzes C-23 hydroxylation of α-solamarine, was conducted in the presence of ^18^O_2_, the reaction product gave 2 mass higher than the product assayed with ^16^O_2_, indicating ^18^O incorporation into the 23-hydroxylated product by Sl23DOX (Supplementary Fig. 20a–c). In contrast, the DPS enzymatic assay in the presence of ^18^O_2_ did not yield any ^18^O-labeled product (Supplementary Fig. 20d–f). This result indicates that the oxy ion at C-16α in the DPS-reaction product is not derived from molecular oxygen and also suggests that the hydroxy moiety introduced by the DPS-dependent oxygenation leaves as water to form the solanidane. Based on these findings, we propose the reaction mechanism shown in Fig. 4d. First, DPS catalyzes hydroxylation at C-16α of spirosolane-skeleton to form a 16-hemiacetal intermediate. Second, the lone pair on the nitrogen atom of the intermediate migrates to between C-22 and the nitrogen atom. Concomitantly, the carbon-oxygen bond at C-22 cleaves, and the hydroxy group at C-16α introduced by DPS leaves as water to afford a 16-oxo species. Third, the nitrogen atom attacks the C-16 carbonyl carbon to form an imine-oxy zwitterion, the DPS reaction product.

Kinetic analyses revealed that the *k*_cat_/*K*_m_ value of DPS for α-solamarine **7** was 1123-fold greater than that for (*22S*,*25S*)-spirosol-5-en-3β-ol **3**, the aglycone of α-solamarine **7** (Supplementary Fig. 21 and Supplementary Table 4). The strong substrate preference of DPS for the glycosides suggests that conversion of spirosolane to solanidane occurs after the glycosylation step at the C-3 hydroxy group (Fig. 1). This is consistent with the observation that α-tomatine **6** was converted to its solanidane glycoalkaloid demissine **11** with retention of the lycotetraose at the C-3 hydroxy group (Supplementary Fig. 4). Recombinant St7070 assays with the above substrates gave the same reaction products as DPS, but the substrate preference of St7070 was significantly different from that of DPS. St7070 showed lower activity for α-solamarine **7** than DPS (Fig. 4a) and also showed only 4.5-fold higher preference for α-solamarine **7** than for its aglycone (*22S*,*25S*)-spirosol-5-en-3β-ol **3** (Supplementary Fig 21 and Supplementary Table 4).

Thus, DPS was found to catalyze the conversion of spirosolanes to form the corresponding solanidanes in vitro, but the resultant DPS-reaction products were not the solanidane end-products completed in planta. To resolve this inconsistency, the DPS-reaction products were fed to 16DOXko HR to examine their conversion to the corresponding end-products in vivo. The DPS-reaction product with α-solamarine **7** was converted into α-solanine **9** in 16DOXko HR (Supplementary Fig. 22). Similarly, administration of the DPS-reaction product with β-solamarine **8** to 16DOXko HR restored the accumulation of α-chaconine **10** (Supplementary Fig. 22). These results indicate that the DPS-reaction products serve as intermediates of solanidane glycoalkaloid biosynthesis in potato.

### Evolution of SGA biosynthetic pathways in *Solanum* species

**Fig. 5:**
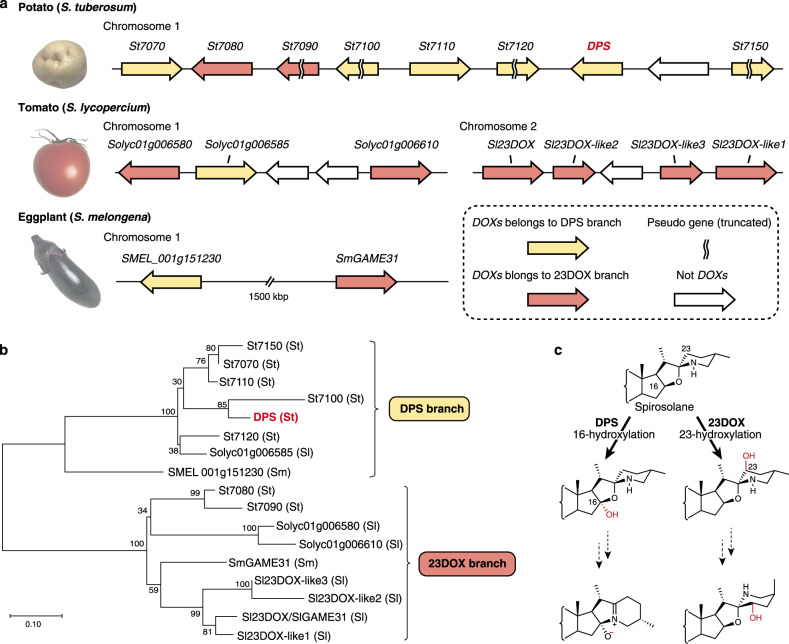
Evolution of the *DPS* homologous genes associated with SGA metabolism in *Solanum* plants. **a** Genomic organization of the *DPS* homologous genes in potato, tomato, and eggplant. Genes belonging to the DPS branch and genes belonging to the 23DOX branch in phylogenetic analysis are shown in yellow arrows and red arrows, respectively. White arrows indicate genes not belonging to the DOX superfamily. **b** Phylogenetic analysis of the *DPS* homologous genes. A phylogenetic tree was generated using the maximum-likelihood method in MEGA X. Bootstrap values based on 1000 replicates are shown at the branching points. St *S. tuberosum* (potato), Sl *S. lycopersicum* (tomato), Sm *S. melongena* (eggplant). **c** Reactions catalyzed by DPS and 23DOX. 23DOX catalyzes C-23 hydroxylation of α-tomatine, and the product spontaneously isomerizes to neorickiioside B, which is an intermediate in α-tomatine metabolism that appears during ripening. Photos of tomato, potato, and eggplant were taken by Ryota Akiyama, Graduate School of Agricultural Science, Kobe University.

With the establishment of DPS-associated metabolic pathway converting spirosolanes to solanidanes, we next linked this discovery to the evolution of SGA metabolism in the *Solanum* genus. We conducted a Basic Local Alignment Search Tool (BLAST) search of DPS against the Tomato ITAG protein database (https://solgenomics.net/). This analysis identified a single distinct homolog Solyc01g006585 in the tomato genome, which shows 85% amino acid sequence identity to DPS. Recombinant Solyc01g006585 catalyzed the same reaction as DPS on the spirosolanes and was inactive with the solanidanes (Supplementary Figs. 23–29). Very low or no expression of *Solyc01g006585* observed in RNA-seq data from the Tomato Functional Genomics Database (http://ted.bti.cornell.edu) (Supplementary Table 5) explains the lack of solanidane glycoalkaloids and accumulation of spirosolane glycoalkaloids (i.e., α-tomatine etc.) in tomato. *Solyc01g006585* is embedded in the gene cluster containing three *DOX* genes in tomato chromosome 1 (Fig. 5a). Genes flanking this cluster showed considerable homology with genes flanking the *DOX* clusters harboring *DPS*, suggesting a common origin of these regions. Additionally, the eggplant DPS homolog, *SMEL_001g151230* is present on the chromosome 1. SMEL_001g151230 protein, which shares 69% amino acid sequence identity to DPS, also had the same catalytic activity as DPS (Supplementary Fig. 30), while the expression of *SMEL_001g151230* is quite low in aerial parts of eggplant^28^ (Supplementary Table 6). These results indicate that the evolution of these orthologous *DPS* genes from duplication and functional divergence of the *DOX* family may have occurred before the speciation of *Solanum* species. Consequently, it is most likely that functional expression of the *DPS* gene or its orthologs is a prerequisite for the endogenous accumulation of toxic solanidane glycoalkaloids in *Solanum* species.

Based on the classification of the plant DOXs^27^, all the clustered DOXs in potato, tomato, and eggplant are classified into the clade DOXC20. DOXC20 includes the α-tomatine 23-hydroxylase (Sl23DOX/GAME31) involved in conversion of bitter α-tomatine **6** to non-bitter esculeoside A during tomato fruit ripening^14^. Sl23DOX/GAME31 shares 55% amino acid sequence identity to DPS, and the homologs of *Sl23DOX* are clustered in tomato chromosome 1 and 2 (Fig. 5a). In the phylogenetic analysis, DPS forms a distinct branch (the DPS branch) which is clearly separated from the 23DOX branch that includes Sl23DOX/GAME31 (Fig. 5b). Among the eight *DOX* genes clustered in potato chromosome 1, *St7070*, *St7100*, *St7110*, *St7120*, *DPS*, and *St7150* are found in the DPS branch while *St7080* and *St7090* are in the 23DOX branch (Fig. 5b). Solyc01g006585 and SMEL_001g151230 are placed in the DPS branch, while the other DOXs clustered in tomato genome are in the 23DOX branch (Fig. 5b). These results indicate that functional diversification between Sl23DOX/GAME31 and DPS may have arisen from a common ancestral DOXC20 before the speciation of *Solanum* species.

## Discussion

In this study, we revealed a key reaction step diverging the biosynthetic pathways of two general classes of SGAs, solanidanes and spirosolanes. By the feeding experiments, we found that solanidane glycoalkaloids are biosynthesized from spirosolanes in potato. Gene silencing experiment and biochemical characterization revealed that potato DPS, belonging to the DOX superfamily, is involved in the metabolic conversion of spirosolanes to solanidanes. Thus, DPS is a key enzyme in an evolutionary origin of the biosynthetic pathway of solanidane glycoalkaloids branched from spirosolanes in potato (Fig. 1).

The strong preference of DPS for glycosides observed in the in vitro assays (Supplementary Table 4) let us hypothesizes that glycosylation at the C-3 hydroxy group occurs before formation of the solanidine backbone. Previously, SGT1 and SGT2 were reported to catalyze the initial transgalactosylation and transglucosylation of solanidine **12** at the C-3 hydroxy group, respectively. These two glycosyltransferases accept spirosolane aglycones as well, and the activities were comparable to the activity for solanidine^21,22^. Additionally, *16DOX*-silencing which disrupts 16α-hydroxylation of 22,26-dihydroxycholesterol resulted in the accumulation of glycosides of 22,26-dihydroxycholesterol **2**^19^. These previous findings support our hypothesis that conversion of spirosolane to solanidane occurs after the glycosylation step at the C-3 hydroxy group (Fig. 1).

Analysis of the catalytic activity of DPS clearly demonstrated that DPS is not sufficient to complete the conversion from spirosolanes to the solanidane end-products accumulated in planta. The structure of the DPS-reaction product allowed us to propose a pathway for completing the solanidane-skeleton formation, which requires two imine-reduction reactions following to DPS reaction (Supplementary Fig. 31). Feeding of the DPS-reaction products to 16DOXko HR indicated their conversion to α-solanine and α-chaconine, suggesting the presence of putative enzyme(s) involved in the reduction of the imine intermediates in vivo (Supplementary Fig. 22). Analysis of transgenic tomato hairy roots constitutively expressing the *DPS* gene detected a new peak, of which the retention time (Rt. 18.7) and mass spectrum matched with those of the reaction product by DPS using α-tomatine as a substrate, but the production of demissine, which is the solanidane end-product derived from α-tomatine, was not detected (Supplementary Fig. 32). These results suggest that, in tomato, putative reductase(s) catalyzing the reduction of the imine intermediate is absent or not expressed. Thus, it is likely that the reductase(s), together with DPS, will be an important enzyme for solanidane glycoalkaloid biosynthesis in potato.

We identified the presence of DPS isoform designated as St7070 in potato genome, and biochemical characterization revealed that St7070 catalyzed the conversion of the spirosolane-skeleton to the solanidane-skeleton. Gene silencing of *DPS* resulted in a significant reduction (~90%) in solanidane glycoalkaloid levels, but no off-target silencing effect on *St7070* was observed (Fig. 3a–c). These results indicate that DPS is the major isoform involved in solanidane glycoalkaloid biosynthesis in potato, although there may be a minor contribution from St7070. We also identified functional DPS orthologs in the non-solanidane producing species, tomato and eggplant, and however, these orthologs were almost not expressed in these species. It has been reported that the transcription factor JRE4/GAME9 comprehensively regulate SGA biosynthetic genes in tomato^10,29,30^. Overexpression of the transcription factor JRE4/GAME9 in potato led to significant increase of DPS transcription levels^10^, while the expression of *Solyc01g006585* was not induced by JRE4/GAME9 overexpression in tomato^10,29,30^. These observations indicate that transcriptional regulation of DPS or its orthologs is a crucial factor diverging between spirosolane and solanidane glycoalkaloids producers. There are two possible evolutionary scenarios; (i) gain of the DPS expression in solanidane-glycoalkaloids producing species such as domesticated potatoes and wild potatoes or (ii) loss of the DPS expression in non-solanidane glycoalkaloids producers such as tomato and eggplant. Further genetic studies are required to clarify the evolutionary events that underpin the differential expression of DPS and its orthologs in *Solanum* species.

Several studies reported that tandem gene duplication and subsequent changes in gene function drive the generation of new biosynthetic pathways or enhancement of existing metabolic flux^31–34^. Therefore, it is noteworthy that the genes belonging to the DPS branch are highly duplicated in tandem on potato chromosome 1 (Fig. 5a). The fact that there is only one DPS ortholog in each tomato and eggplant genome indicates that the clustered genes including *DPS* and *St7070* in potato have arisen from a single-copy ancestral gene via gene duplication after the speciation of *Solanum* species. *DPS* encodes a characteristic shorter polypeptide missing about 40 amino acid residues at the C-terminus as compared to typical DOXC members^14,19,27^ (Supplementary Fig. 33). Nucleic acid sequence alignment suggests that DPS was modified to become shorter via a single nucleotide-insertion (Supplementary Fig. 34). In addition, DPS and St7070 exhibited markedly differences in substrate specificity and catalytic efficiency, with DPS having very strong substrate preference for glycosides compared to St7070 (Supplementary Table 4). Given the major contribution of DPS to solanidane glycoalkaloids biosynthesis, we presume that such improved catalytic property of DPS, in addition to its elevated gene expression, provide an evolutionary benefit by providing the metabolic pathways from spirosolane to solanidane glycoalkaloids in cultivated potatoes. Several wild potato species contain both spirosolanes and solanidanes^12,25^, whereas domesticated potato cultivars produce almost only solanidane glycoalkaloids. To clarify the underlying mechanism of the different glycoalkaloid profiles between wild and cultivated potatoes, further experiments are needed, such as analyzing differences in gene expression, coding sequences, and catalytic properties for *DPS* and its homologous genes between wild and cultivated potatoes.

The SGA biosynthetic pathway is widely conserved among *Solanum* species^18,28^, and evolutionary divergence of spirosolane-metabolizing DOXs contributes to the chemical diversity of SGAs in the *Solanum* genus^15^. Recent studies identified that Sl23DOX/GAME31 in tomato catalyzes C-23 hydroxylation of α-tomatine^14,15^ (Fig. 5c), and this reaction is responsible for the first step of detoxification metabolism from toxic spirosolane glycoalkaloid α-tomatine to non-toxic esculeoside A. Phylogenetic analysis indicates that functional differentiation between Sl23DOX/GAME31 and DPS in the DOXC20 subfamily likely has arisen from a common ancestral enzyme (Fig. 5). In tomato, spirosolane-metabolizing Sl23DOX/GAME31 contributes to the domestication of cultivated tomatoes by the reduction of toxicity and bitterness in mature fruits. In contrast, metabolism of spirosolane glycoalkaloids to solanidane glycoalkaloids in potato may represent evolutionary advantage to produce SGAs more toxic and effective in protection against their enemies.

In conclusion, we have revealed the genetic and enzymatic origin of solanidane glycoalkaloid biosynthesis in potato. This provides insight into the evolution of chemical diversity of SGAs in *Solanum* species and its evolution’s footprints on *Solanum* genomes. Thus, our findings further elucidate understanding of how plants have evolved and developed their specialized metabolic pathways, demonstrating that evolutionary divergence of the DOX family is one of the key driving forces for phytochemical diversity.

## Methods

### Chemicals

Authentic samples of α-solanine **9**, α-chaconine **10**, and solanidine **12** were purchased from Sigma-Aldrich, and α-tomatine **6** was purchased from Tokyo Chemical Industry Co., Ltd. (*22S*,*25S*)-spirosol-5-en-3β-ol **3** were isolated in our laboratory from tomatidine **4** (Chromadex), respectively, using HPLC^35,36^. α-Solamarine **7** and β-solamarine **8** were purified from the diploid potato clone 97H32-6 and the chemical structures were confirmed using NMR in our laboratory^37^.

### Feeding experiments

*St16DOX*-disrupted potato hairy roots were established in our laboratory^26^ and subcultured every month on an orbital shaker (100 rpm) in Gamborg B5 (B5) liquid medium containing 2% (w/v) sucrose and cefotaxime (250 μg ml^−1^) at 20 °C under 24-h dark conditions and used for the feeding experiments. α-Solamarine **7**, β-solamarine **8**, and (*22S*,*25S*)-spirosol-5-en-3β-ol **3** (MeOH solution, final concentration 10 μM) were added individually to the liquid medium of 3-week-old hairy roots. For the inhibition experiment, prohexadione (acetone solution) or uniconazole-P (acetone solution) was concomitantly added to the aquaculture with α-solamarine **7**. After 3 days of cultivation, SGAs contained in the harvested hairy roots were extracted three times from 100 mg fresh samples with 300 μl methanol. The extracting solution was evaporated and the residue dissolved in 200 μl methanol. After centrifugation, 10 μl of supernatant was diluted with 290 μl methanol and an aliquot (2 μl) of the filtrate was analyzed using LC–MS. The analysis was performed using an ACQUITY UPLC H-Class System (Waters) with an SQ Detector 2 (Waters); data acquisition and analyses were performed using MassLynx 4.1 software (Waters). Each sample was injected into an ACQUITY UPLC HSS T3 chromatographic column (100 × 2.1 mm, 1.7 μm; Waters), with a column temperature of 40 °C and flow rate of 0.2 ml min^−1^. The mass spectra were obtained in positive electrospray ionization mode, with a capillary voltage of 3 kV and a sample cone voltage of 60 V. Mass spectrometry scan mode with a mass range of *m*/*z* 350–1250 was used. The mobile phases were water with 0.1% (v/v) formic acid (A) and acetonitrile (B), using gradient conditions as follows: solvent B ramped linearly from 10% to 42.5% over 15 min; solvent B increased linearly to 100% over 4 min, held at solvent B 100% for 5 min; solvent B then returned immediately to 10%, followed by a 5 min re-equilibration period. In the α-tomatine **6** feeding experiment, the mobile phases were water with 0.1% (v/v) formic acid (A), acetonitrile (B), and methanol (C), with a linear gradient elution as follows: from solvent A 90%/solvent B 5%/solvent C 5% to solvent A 45%/solvent B 27.5%/solvent C 27.5% over 30 min; from solvent A 45%/solvent B 27.5%/solvent C 27.5% to solvent A 25%/solvent B 37.5%/solvent C 37.5% over 5 min; from solvent A 25%/solvent B 37.5%/solvent C 37.5% to solvent A 0%/solvent B 50%/solvent C 50%, held at solvent A 0%/solvent B 50%/solvent C 50%; solvent B and solvent C then returned immediately to 5%; followed by a 5 min re-equilibration period. Conversion of α-tomatine **6** to corresponding solanidane was confirmed by comparison with demissine **11** extracted from leaves of the wild potato *S. acaule*^38^.

### Real-time quantitative RT-PCR analysis

Total RNA was extracted using the RNeasy Plant Mini Kit (Qiagen) and the RNase-Free DNase Set (Qiagen). Total RNAs of potato (*S. tuberosum* cv Sassy) were prepared from the leaves, stems, flowers, roots, stolons, tuber peels, and tuber sprouts. The extracted total RNAs of potato were used to synthesize first-strand cDNAs, using the ReverTra Ace^®^ qPCR RT Master Mix with gDNA remover. Quantitative reverse transcriptional-polymerase chain reaction (RT-PCR) was performed with a LightCyclerNano (Roche), using FastStart Essential DNA Green Master (Roche) with the following primer sets: 1 and 2 for *DPS*, 3 and 4 for *St7070*, and 5 and 6 for *EF1α*, which is a housekeeping gene used for real-time RT-PCR normalization^39^ (Supplementary Table 7). Cycling was carried out as follows: 95 °C for 10 min, 45 cycles at 95 °C for 10 s, 60 °C for 10 s, amplification at 72 °C for 15 s, and holding at 95 °C for 30 s. This was followed by ramping up from 60 to 95 °C at 0.1 °C s^−1^ to perform a melting curve analysis. Three biological replicates were analyzed in duplicate. The values obtained for the *EF1α* gene were used as internal references in potato, and the gene expression levels were normalized against these values. Data acquisition and analysis were performed using LightCyclerNano software (Roche).

### Cloning of *DPS* and *St7070* cDNAs

The potato cDNA template was prepared from mRNA isolated from sprouts of *S. tuberosum* cv Sassy, as described above. The cDNA fragments that contained the open-reading frames of *DPS* and *St7070* were amplified by RT-PCR, using cDNA from tuber sprouts as a template, with primers 7 and 8 for *DPS* and primers 9 and 10 for *St7070* (Supplementary Table 7). These primers were designed from the potato unigene sequences Sotub01g007130 and Sotub01g007070, respectively, using the Potato ITAG protein database (http://solanaceae.plantbiology.msu.edu/index.shtml). The PCR products were cloned into the pCR^®^4 Blunt-TOPO^®^ vector (Thermo Fisher Scientific).

### Generation of *DPSi* transgenic potato plants

The *DPS-*RNAi binary vector carried the inverted repeat of the partial *DPS* fragment interposing the third intron of the Arabidopsis (*Arabidopsis thaliana*) *At4g14210* gene^40^ under the control of the *cauliflower mosaic virus* 35S (CaMV35s) promoter in the T-DNA region. pRI201-AS (TaKaRa) was digested with *Kpn*I and *Sma*I to disrupt multi-cloning site 2. The protruding end of the digested site was blunted with T4 DNA polymerase (TaKaRa) and then the blunted ends were ligated. The resulting plasmid was named pRI201-*Δ*MCS2 in this work. The third intron of *At4g14210* was PCR amplified from pKT258^19^ with primers 11 and 12, which contained restriction sites (Supplementary Table 7). The amplified fragments were cloned into the *Xba*I and *Nde*I sites of pRI201-*Δ*MCS2, and the resulting plasmid was named pRI201-*Δ*MCS2_PDS3. A 262 bp fragment of the *DPS* coding region was PCR-amplified using primers 13 and 14 (Supplementary Table 7). The fragment was inserted into the *Xba*I/*Bam*HI and *Nde*I/*Sal*I sites of pRI201-*Δ*MCS2_PDS3 to construct a *DPSi* vector. Potatoes (*S. tuberosum* cv Sassy) were then transformed using *A. tumefaciens* EHA105 cells harboring the *DPSi* vector. Briefly, internodal stem sections were excised from in vitro-grown potato shoots and cut into ~5 mm sections. Explants were co-cultivated with agrobacterium for 48 h at 20 °C on MS medium containing 3% (w/v) sucrose, *trans*-zeatin (2 μg ml^−1^), indole-3-acetic acid (50 ng ml^−1^), kanamycin (50 μg ml^−1^), and acetosyringone (100 μM) under dark conditions. After the co-cultivation period, the stem pieces were transferred to shoot induction medium composed of agarose-solidified MS medium containing 3% (w/v) sucrose, *trans*-zeatin (2 μg ml^−1^), indole-3-acetic acid (50 ng ml^−1^), kanamycin (50 μg ml^−1^), and meropenem hydrate (50 μg ml^−1^), and cultured at 20 °C under 16-h/8-h light/dark cycles. Stem pieces were transferred to fresh shoot induction medium every 2 weeks, until plantlets emerged. Well-developed plantlets were excised and transferred to MS medium containing 3% w/v sucrose, kanamycin (50 μg ml^−1^), and meropenem hydrate (50 μg ml^−1^). After 2–3 weeks, well-rooted shoots were selected and transformants were individually screened by genomic PCR using primer sets 15 and 16 (Supplementary Table 7), which targeted the kanamycin-resistance gene in the T-DNA region that was integrated into the genome.

### Characterization of *DPSi* transgenic potato plants

Total RNA was then prepared from the leaves of four independent lines of the following in vitro-cultured plants: #13, #17, and #21. Quantitative RT-PCR analysis of *DPS* and *St7070* were performed using primers 1/2 and 3/4, respectively (Supplementary Table 7), as described above. The SGAs that accumulated in the *DPSi* transgenic plants were extracted as described previously^19^ with minor modification: Briefly, 50 mg of fresh plant material was frozen and homogenized with a mixer mill at 4 °C. The homogenate was then extracted with 300 μl MeOH. After centrifugation, the supernatant was collected. The extraction was repeated three times and the extract, dried in vacuo, was re-dissolved in 400 μl MeOH. A 5 μl sample of solution was diluted with 295 μl MeOH and filtered through a 0.22-μm polytetrafluoroethylene membrane filter. An aliquot (2 μl) was then analyzed by UPLC–MS. LC–MS analysis was performed as described above, under “Feeding experiments”, with minor modifications. Briefly, the mobile phases were water with 0.1% (v/v) formic acid (A) and acetonitrile (B), using the following gradient conditions: solvent B ramped linearly from 10% to 32.5% over 30 min; solvent B increased linearly to 100% over 1 min, held at 100% solvent B for 4 min; solvent B then returned immediately to 10%; followed by a 5 min re-equilibration period. The mass spectra were obtained in positive electrospray ionization mode, with a capillary voltage of 3 kV and a sample cone voltage of 60 V. Mass spectrometry scan mode with a mass range of *m/z* 350–1250 was used. Quantification of SGAs was carried out using selected ion monitoring (SIM). α-Solanine **9**, α-chaconine **10**, α-solamarine **7**, and β-solamarine **8** were monitered at *m/z* 868, 852, 884, and 868, respectively, and were quantified using the calibration curves of the authentic samples.

### Expression of the recombinant proteins in *E. coli*

The coding sequences of *DPSi* and *St7070* were digested from the pCR^®^4 Blunt-TOPO^®^ vector (Thermo Fisher Scientific) with *Nde*I and *Sal*I. Since *Solyc01g006580* could not be amplified from cDNA in any tissue in tomato, the coding sequence of *Solyc01g006580* with *Nde*I and *Sal*I sites was synthesized (Eurofins Genomics Inc.) The DNA fragments were then ligated into the *Nde*I-*Sal*I sites of pCold ProS2 (TaKaRa). *E. coli* strain BL21 (DE3) (Clontech) transformed with the constructed plasmid was grown at 37 °C in LB medium containing ampicillin (50 μg ml^−1^) until the OD_600_ reached 0.5. Recombinant protein production was induced by adding 0.1 mM isopropyl β-d-1-thiogalactopyranoside and the culture was grown for 24 h at 15 °C. The culture was then centrifuged at 7720 × *g* for 10 min at 4 °C, and the cell pellets were resuspended in 5 ml of cold sonication buffer containing 50 mM Bis–Tris–HCl (pH7.2), 150 mM NaCl, 10% (v/v) glycerol, and 5 mM dithiothreitol. The solution was then sonicated three times for 30 s each on ice using a Bandelin Sonopuls HD 2070 ultrasonic homogenizer type MS73 (Sigma-Aldrich), at a sound intensity of 200 W cm^−2^ and centrifuged at 20,630x*g* for 10 min at 4 °C. The recombinant His-tagged protein contained in the soluble fraction was purified using a His SpinTrap TALON column (GE Healthcare), according to the manufacturer’s instructions. After two column washes, the adsorbed proteins were eluted twice in 200 μl of an elution buffer containing 50 mM sodium phosphate (pH 7.4), 30 mM NaCl, and 150 mM imidazole, and the elution was mixed. The purified recombinant proteins were used for further analyses.

### In vitro activity assay of the recombinant enzymes

The SGAs and their aglycones, α-solamarine **7**, β-solamarine **8**, α-tomatine **6**, α-solanine **9**, α-chaconine **10**, (*22S*,*25S*)-spirosol-5-en-3β-ol **3**, tomatidine **4**, and solanidine **12**, were used as substrates in recombinant protein assays. An in vitro enzyme activity assay was performed using 100 μl of reaction mixture containing 100 mM Bis–Tris–HCl (pH 7.2), 5 mM 2-oxoglutarate, 10 mM sodium ascorbate, 0.2 mM FeSO_4_, 25 μM substrate, and 1 μg purified recombinant proteins as the enzyme. The reaction was initiated by the addition of the enzyme and was carried out at 30 °C for 30 min. The reaction was then stopped by incubation for 2 min at 90 °C. After centrifugation, 20 μl of supernatant was diluted with 180 μl methanol and filtered through 0.2-μm nylon membrane filters (Acrodisc, Waters). An aliquot (2 μl) was analyzed using LC–MS, as described in “Feeding experiments” above. We determined the kinetic parameters of the recombinant DPS and St7070 in triplicate assays. The assays were conducted as described above, with minor modifications. Briefly, 0.1 μg purified DPS protein or 0.02 μg purified St7070 were used. The activity was assayed using α-solamarine **7** or (*22S*,*25S*)-spirosol-5-en-3β-ol **3** at concentrations ranging from 0.25 to 50 μM, and the reaction was carried out at 30 °C for 10 min. A 20 μl sample of assay mixture was diluted with 180 μl methanol and filtered through 0.2-μm nylon membrane filters (Acrodisc, Waters). An aliquot (2 μl) was analyzed using LC–MS, as described above. The kinetic parameters were determined by nonlinear regression, using the ANEMONA program^41^.

### ^18^O_2_-treated assay

A freshly prepared solution (1 ml) containing 100 mM Bis–Tris–HCl (pH 7.2), 5 mM 2-oxoglutrate, 10 mM sodium ascorbate, 0.2 mM FeSO_4_, 25 μM α-solamarine, was introduced into 10 ml round bottomed flask with a stir bar. Then, the flask was capped by a three-way cock which was connected to a vacuum pump line and a balloon filled with ^18^O_2_, respectively. The reaction mixture was degassed in vacuo, and then ^18^O_2_ was incorporated into the reaction mixture by purging once and stirring with a stirrer bar. An aliquot of the reaction mixture was quickly transferred to 1.5 ml microtube and mixed with the purified enzyme. The reaction was carried out at 30 °C for 30 min. The reaction was then stopped by incubation for 2 min at 90 °C. After centrifugation, 20 μl of supernatant was diluted with 180 μl methanol and filtered through 0.2-μm nylon membrane filters (Acrodisc, Waters). An aliquot (2 μl) was analyzed using LC–MS, as described in “Feeding experiments” above.

### **S**tructural determination of the DPS enzymatic reaction product

The DPS enzymatic reaction with α-tomatine **6** was performed using 50 ml of reaction mixture as described above, with a minor modification, to determine the structure of the product of the enzymatic reaction catalyzed by recombinant DPS: the reaction was conducted using the crude enzyme overnight. The reaction mixture was extracted with water-saturated butanol after the pH was adjusted to 3.0 by the addition of hydrochloric acid. The butanol layer was corrected and, before the dried residue was dissolved in methanol, DPS product was concentrated in vacuo. The DPS reaction product dissolved in methanol was subjected to preparative scale high performance liquid chromatography (HPLC). The sample was injected into an ODS column (COSMOSIL 5C_18_-PAQ, 20 × 250 mm, Nacalai) at a column temperature of 40 °C, and the flow rate was set to 4 mL min^−1^. Separation was performed with isocratic mobile phases of 22% acetonitrile in H_2_O containing 0.1% trifluoroacetic acid (v/v), and the elute was detected at 203 nm. Then, the separated DPS reaction product was concentrated.

The molecular formula of the DPS enzymatic reaction product was determined to be C_50_H_81_NO_21_, based on HRMS (FAB) analysis: found *m/z* 1032.5376 [M + H]^+^ (calcd. for C_50_H_82_NO_21_, *m/z* 1032.5380 [M + H]^+^; Δppm = 0.1) (Supplementary Fig. 17). Its structure was determined by two-dimensional NMR measurements, based on correlation spectroscopy (COSY), nuclear Overhauser effect spectroscopy (NOESY), heteronuclear single-quantum correlation spectroscopy (HSQC), and heteronuclear multiple-bond correlation spectroscopy (HMBC) techniques (Supplementary Data 1). The NMR measurements in this study were carried out in CD_3_SOCD_3_ and CD_3_OD. The ^13^C and ^1^H assignments are summarized in Supplementary Tables 3 and 4, and COSY and HMBC correlations are shown in Supplementary Fig. 19. Samples were dissolved in NMR solvent immediately prior to measurement. In CD_3_OD, signals corresponding to H-23, observed at 2.84 ppm (br ddd, *J* = 21.3, 8.9, 6.8 Hz) and 3.12 ppm (1H, br ddd, *J* = 21.3, 4.1, 4.1) at the beginning, disappeared within 21 h. This observation indicated H–D exchange in CD_3_OD. Accordingly, C-23 was observed as a severely broadened signal in CD_3_OD by C–D coupling. In both solutions, C-16 signals were observed at 110.95 ppm (in CD_3_SOCD_3_) and 117.85 ppm (in CD_3_OD), indicating their acetal like nature. Since C-16 of α-tomatine (the substrate of the DPS reaction) bears two carbon, one oxygen, and one hydrogen atom, the chemical shift in C-16 of the DPS reaction product indicates that oxidation occurred on C-16. In contrast, the chemical shift of C-22, within the hemiaminal structure of α-tomatine, observed at 192.21 ppm (in CD_3_SOCD_3_) and 196.80 ppm (in CD_3_OD) in the DPS product at first suggested that C-22 exists as a carbonyl carbon of ketone or aldehyde. However, the HMBC correlation between H-26 and C-22 indicated that C-22 exists as an imine carbon instead of a carbonyl carbon. Since chemical shifts of typical imine carbons are observed around 140–170 ppm^42^, the chemical shift of C-22 of the DPS product appeared at a considerably lower field density. The observation indicated the presence of an iminium ion structure in the DPS product, and the reported ^13^C chemical shift of tri- or tetra-substituted carbon atoms of iminium ions (over 180 ppm, Supplementary Fig. 35)^43^ is comparable to the C-22 chemical shift of the DPS reaction product. In particular, the chemical shift of the tetra-substituted iminium ion in Supplementary Fig. 35 (195.1 ppm) is in good accordance with those observed in the DPS product (192.21 or 196.80 ppm). Furthermore, the NOE correlation between H-26_b_ and H-15β indicated the proximity of these hydrogen atoms. Additionally, the above-mentioned ^13^C chemical shift of C-16 indicated that not only one oxygen atom but also one nitrogen atom binds to C-16. Accordingly, the structure of the DPS product was determined to be a zwitterion species, as shown in Supplementary Fig. 18, in which an iminium ion structure is constructed between C-16, the nitrogen atom, and C-22. The previously mentioned H–D exchange of H-23 in CD_3_OD, which further confirmed the iminium ionic form of the DPS product, is illustrated in Supplementary Fig. 36. The cationic nature of the nitrogen atom would increase the acidity of H-23 and accelerate the H–D exchange reaction.

### Phylogenetic analysis

The amino acids sequences of St7070 (Sotub01g007070), St7080 (Sotub01g007080), St7090 (Sotub01g007090), St7100 (Sotub01g007100), St7110 (Sotub01g007100), St7120 (Sotub01g007120), DPS (Sotub01g007130), and St7150 (Sotub01g007150) were obtained from the Spud DB potato genomics resource (http://solanaceae.plantbiology.msu.edu/). Amino acid sequences of Sl23DOX/GAME31 (Solyc02g062460), Sl23DOX/GAME31-like1 (Solyc02g06250), Sl23DOX/GAME31-like2 (Solyc02g062470), Sl23DOX/GAME31-like3 (Solyc02g062490), Solyc01g006580, Solyc01g006585, and Solyc01g006610 were downloaded from the Sol Genomics Network (https://solgenomics.net). Information about these DOX genes is summarized in Supplementary Data 2. Sequence alignments were performed using MUSCLE^44^ and the maximum-likelihood tree was inferred in MEGA10^45^, using 1000 bootstrap replications.

### Overexpression of *DPS* in tomato hairy roots

A binary vector for *DPS* overexpression was constructed from the binary vector pBINPLUS^46^: the coding region of *DPS* fused with the 5′-UTR of the *A. thaliana* alcohol dehydrogenase (*AtADH*) gene was inserted under the control of cauliflower mosaic virus 35S (CaMV35S) promoter and the heat shock protein terminator in the T-DNA region. The constructed binary vector was electroporated into *A. rhizogenes* ATCC15834. To generate transgenic hairy roots, tomato hypocotyls from 7-day-old seedlings were infected with *A. rhizogenes* strain ATCC15834 harboring a binary vector as described previously^29^. Briefly, bottom end of a hypocotyl segments (1.5 cm in length) were touched to a bacterial colony and then the segments were stood on the on solidified B5 medium containing 2% (w/v) sucrose with the contacted end up. Hairy roots emerging from infected sites were excised and subcultured twice every week on solidified B5 medium containing 2% (w/v) sucrose, 300 mg l^−1^ cefotaxime for disinfection, and 50 mg l^−1^ kanamycin for drug resistance selection. The selected lines were maintained by subculturing every week in 100 ml glass flasks filled with 20 ml of liquid B5 medium supplemented with 2% (w/v) sucrose with shaking at 100 rpm. in the dark. The accumulated SGAs in DPS overexpressed tomato hairy roots were extracted as described above in “Feeding experiment” and then analyzed using LC–MS by the same condition as α-tomatine **6** feeding experiment in “Feeding experiment”.

### Reporting summary

Further information on research design is available in the Nature Research Reporting Summary linked to this article.

## Supplementary information

Supplementary Information

Descriptions of Additional Supplementary Files

Supplementary Data 1

Supplementary Data 2

Reporting Summary

## Data Availability

Sequence data for DPS (LC547753), St7070 (LC547754), and Solyc01g006585 (LC547755) are deposited in DDBJ. All data is available in the main text or the supplementary materials. Data supporting the findings of this study are available within the article and the supplementary materials. Source data are provided with this paper.

## Source data

Source Data
