# Supplementary material for: The biosynthetic pathway of potato solanidanes diverged from that of spirosolanes due to evolution of a dioxygenase
Source: Nat Commun. 2021 Feb 26;12:1300. doi: 10.1038/s41467-021-21546-0 (PMC7910490; doi:10.1038/s41467-021-21546-0)
Supplement: Supplementary file 1 — Supplementary Information [file 41467_2021_21546_MOESM1_ESM.pdf]

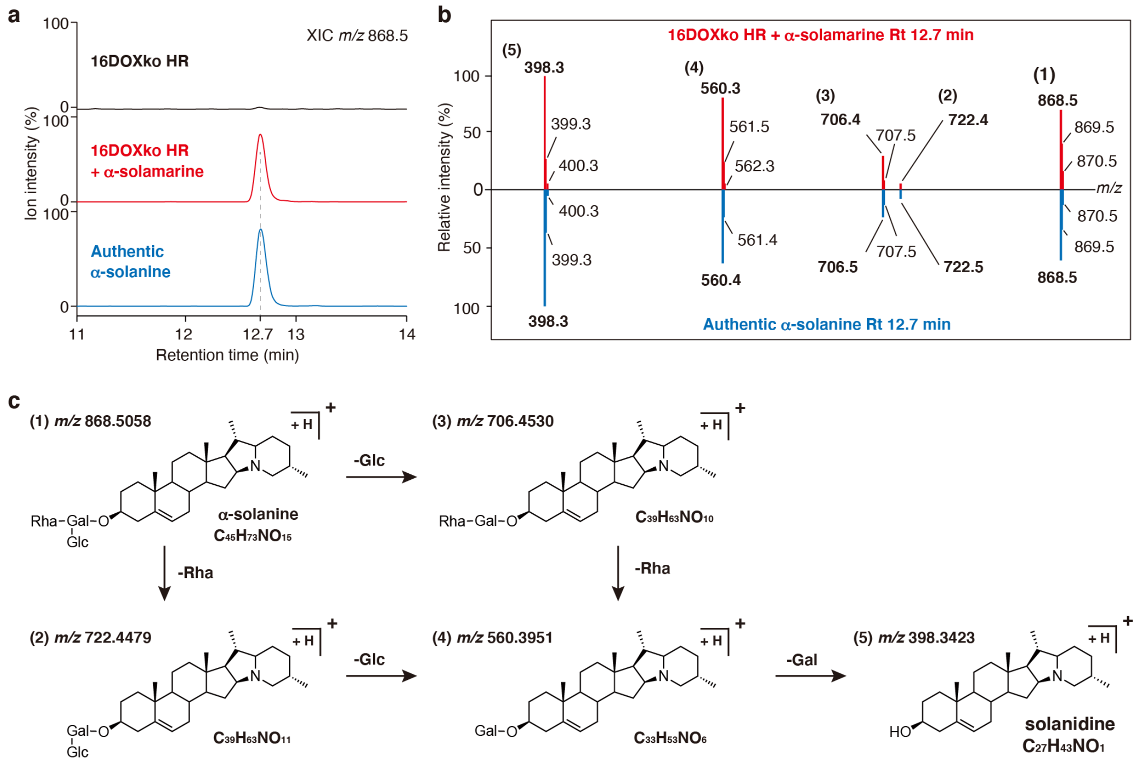

Supplementary Fig. 1| LC-MS analysis of conversion from administered α-solamarine to α-solanine in 16DOX-disrupted potato hairy roots. a Extracted ion chromatograms of *m/z* 868.5 $[M+H]^+$, parental mass of α-solanine **9**. **b** ESI mass fragmentation spectra. Both MS data were obtained in positive ionization mode with a full-scan range of 350-1250 *m/z*. The spectra verify that, despite their different origins, the products are chemically identical. All spectra were obtained from the center of the peaks. **c** MS fragmentation pathways for α-solanine **9**. Abbreviations: 16DOXko HR, 16DOX-disrupted potato hairy roots; XIC, extracted ion chromatogram; Rt, retention time; *m/z*, mass to charge; Glc, glucose; Gal, galactose; Rha, rhamnose.

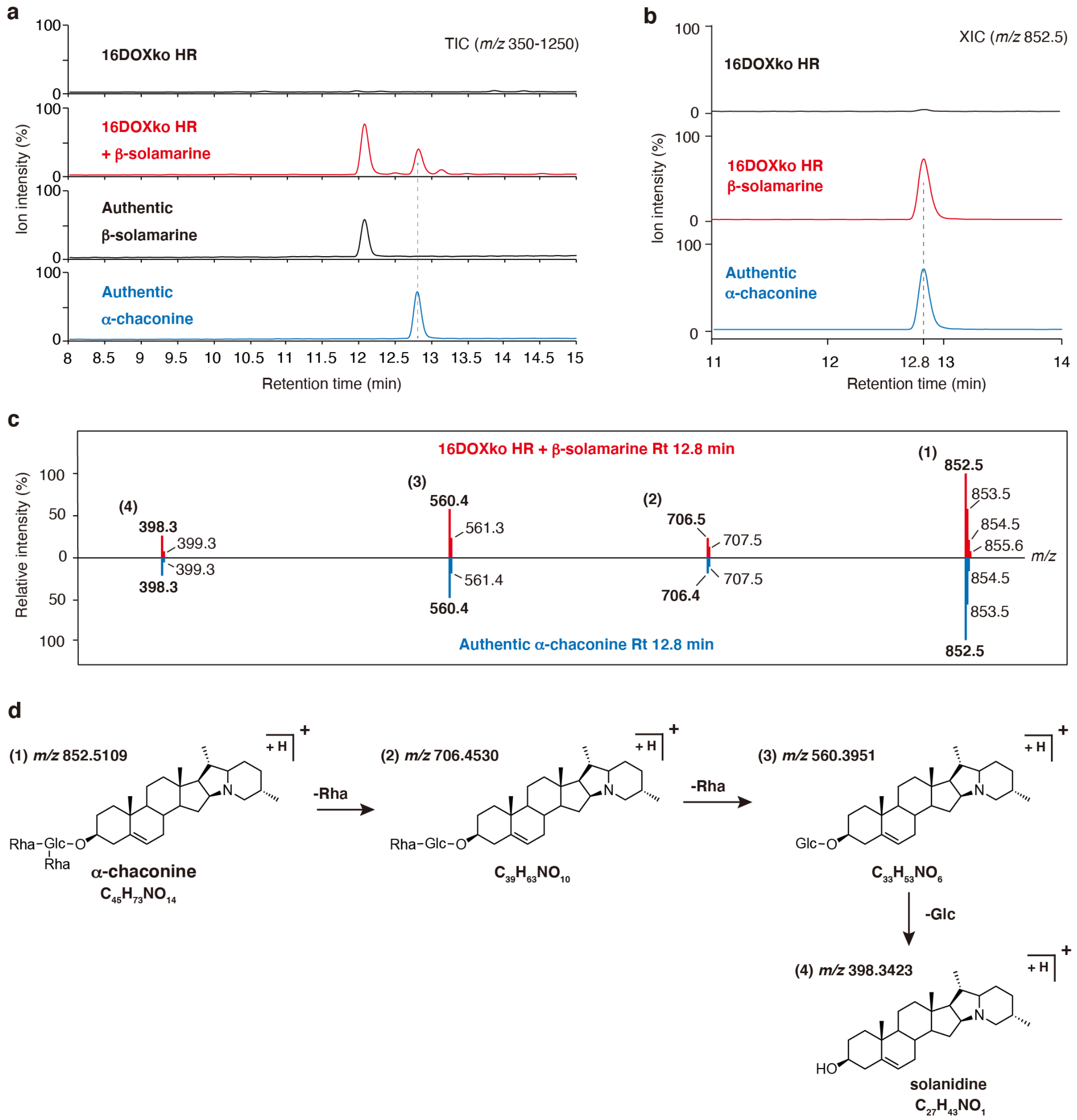

Supplementary Fig. 2| Bioconversion of β-solamarine to α-chaconine in 16DOX-disrupted potato hairy roots. Total SGAs extracted from 16DOX-disrupted potato hairy roots treated with β-solamarine **8** were analyzed using LC-MS. **a** Total ion current chromatogram obtained in positive ionization mode with a full-scan range of 350-1250 *m/z*. **b** Extracted ion chromatograms of *m/z* 852.5 $[M+H]^+$, parental mass of α-chaconine **10**. **c** ESI mass fragmentation spectra. The spectra verify that, despite their different origins, the products are chemically identical. All spectra were obtained from the center of the peaks. **d** MS fragmentation pathways for α-chaconine **10**. Abbreviations: 16DOXko HR, 16DOX-disrupted potato hairy roots; XIC, extracted ion chromatogram; Rt, retention time; *m/z*, mass to charge; Glc, glucose; Rha, rhamnose.

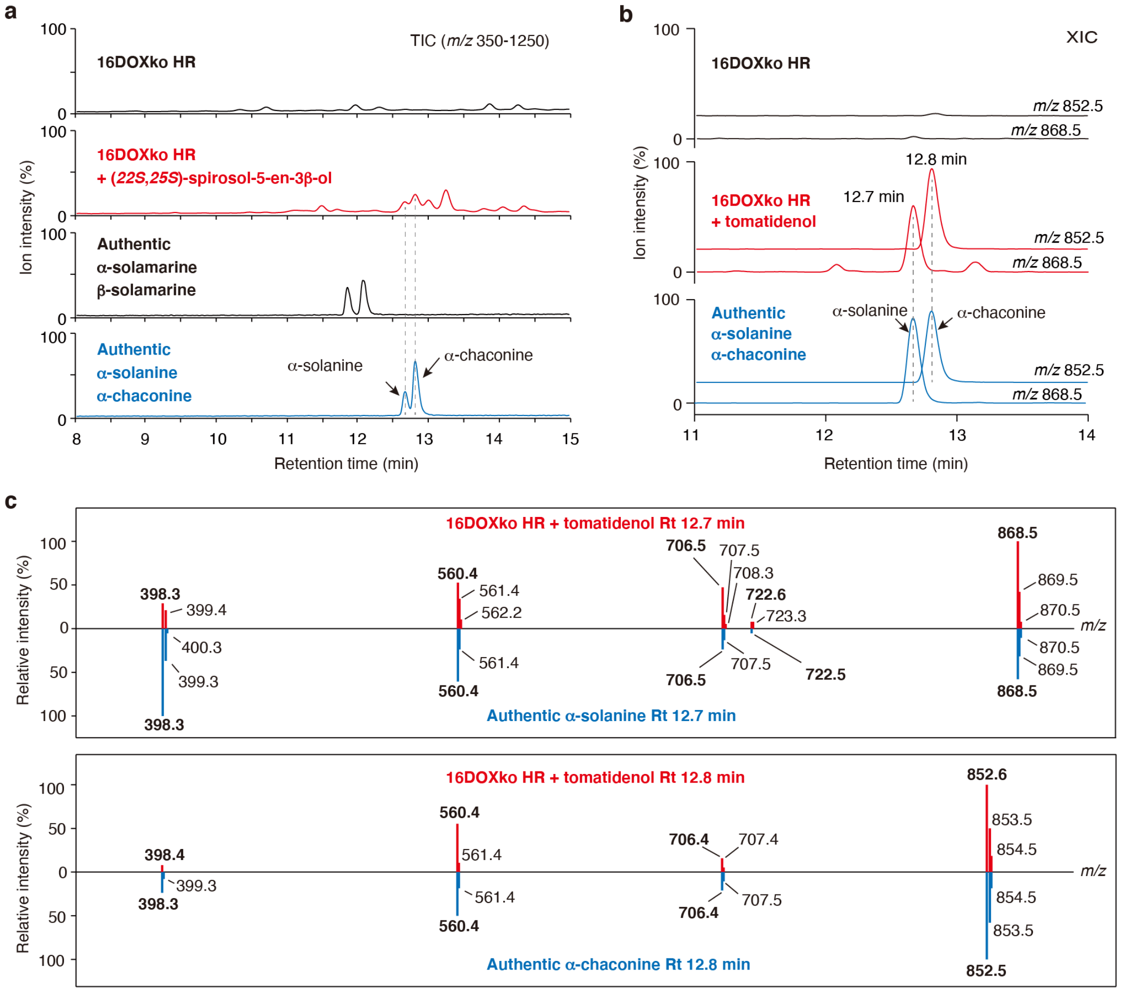

Supplementary Fig. 3| Bioconversion of (*22S*,*25S*)-spirosol-5-en-3β-ol in 16DOX-disrupted potato hairy roots. Total SGAs extracted from 16DOX-disrupted potato hairy roots treated with (*22S*,*25S*)-spirosol-5-en-3β-ol **3** were analyzed using LC-MS. **a** Total ion current chromatogram obtained in positive ionization mode with a full-scan range of 350-1250 *m/z*. **b** Extracted ion chromatograms of *m/z* 852.5 $[M+H]^+$ and 868.5 $[M+H]^+$, parental mass of α-chaconine **10** and α-solanine **9**, respectively. **c** ESI mass fragmentation spectra. The spectra verify that, despite their different origins, the products are chemically identical. All spectra were obtained from the center of the peaks. Abbreviations: 16DOXko HR, 16DOX-disrupted potato hairy roots; XIC, extracted ion chromatogram; Rt retention time; *m/z*, mass to charge.

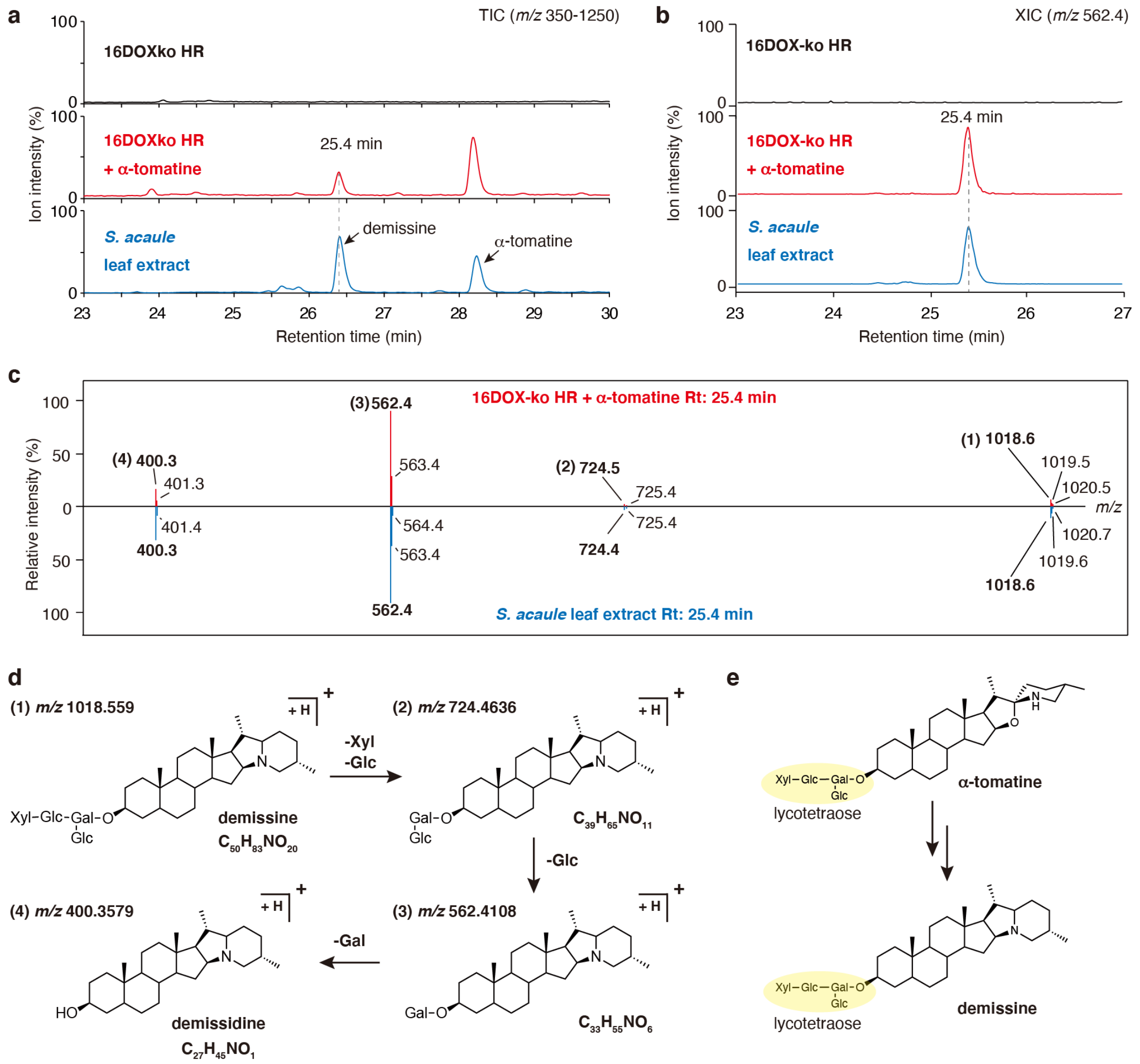

Supplementary Fig. 4| Bioconversion of α-tomatine to demissine in 16DOX-disrupted potato hairy roots. Total SGAs extracted from 16DOX-disrupted potato hairy roots treated with α-tomatine **5** were analyzed using LC-MS. **a** Total ion current chromatogram obtained in positive ionization mode with a full-scan range of 350-1250 *m/z*. **b** Extracted ion chromatograms of *m/z* 562.4 $[M+H\text{-}Xyl\text{-}2{*}Glc]^{+}$, fragment mass of demissine **11**. **c** ESI mass fragmentation spectra. The spectra verify that, despite their different origins, the products are chemically identical. All spectra were obtained from the center of the peaks. **d** MS fragmentation pathways for demissine **11**. **e** Conversion of α-tomatine **6** to demissine **11** with retention of the lycotetraose attached at the hydroxy group at C-3. Abbreviations: 16DOXko HR, 16DOX-disrupted potato hairy roots; XIC, extracted ion chromatogram; Rt, retention time; *m/z*, mass to charge; Glc, glucose; Gal, galactose; Xyl, xylose.

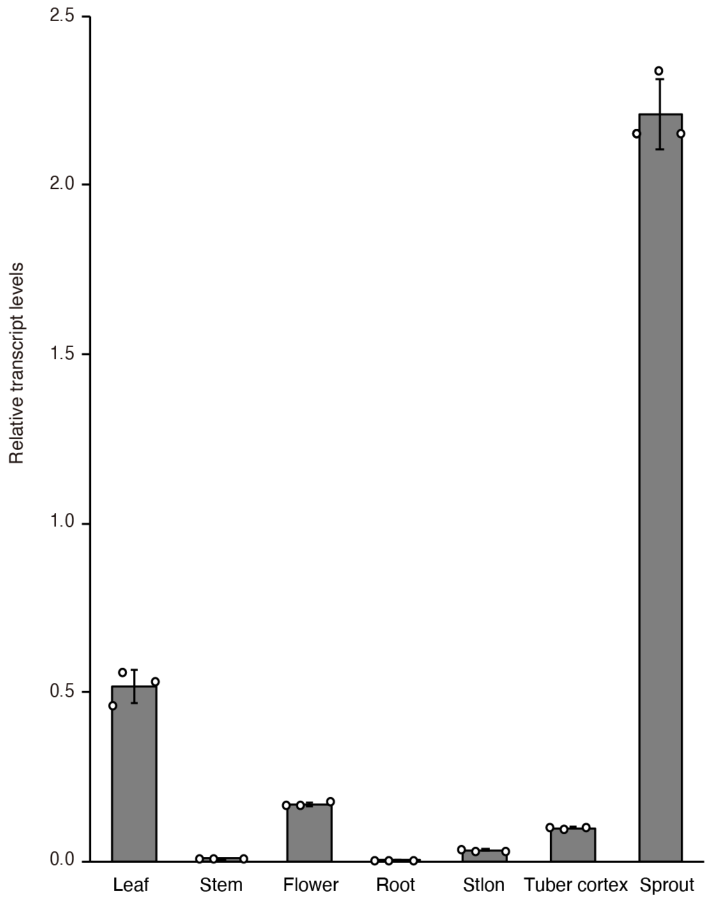

Supplementary Fig. 5| Quantitative RT-PCR analysis of DPS expression patterns in various organs of potato plants. Transcript levels of SGA biosynthetic genes are shown relative to those of *EF1α* as an internal reference gene. Error bars represent ± standard deviation of mean (n = 3). Each biological replicate represents an independently grown plant.

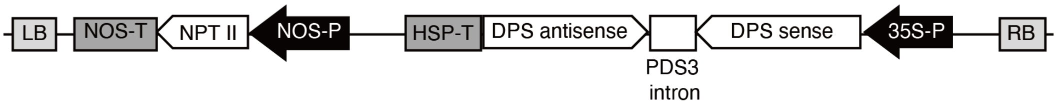

Supplementary Fig. 6| *DPS*-knockdown vector. Abbreviations: 35S-P, Cauliflower mosaic virus 35S promoter; PDS3, the Arabidopsis phytoene desaturase gene (PDS, *At4g14210*); HSP-T, Arabidopsis heat shock protein gene-derived terminator; NOS-P, nopaline synthase promoter; NPT II, neomycin phosphotransferase II; NOS-P, nopaline synthase terminator; LB, left border; RB, right border.

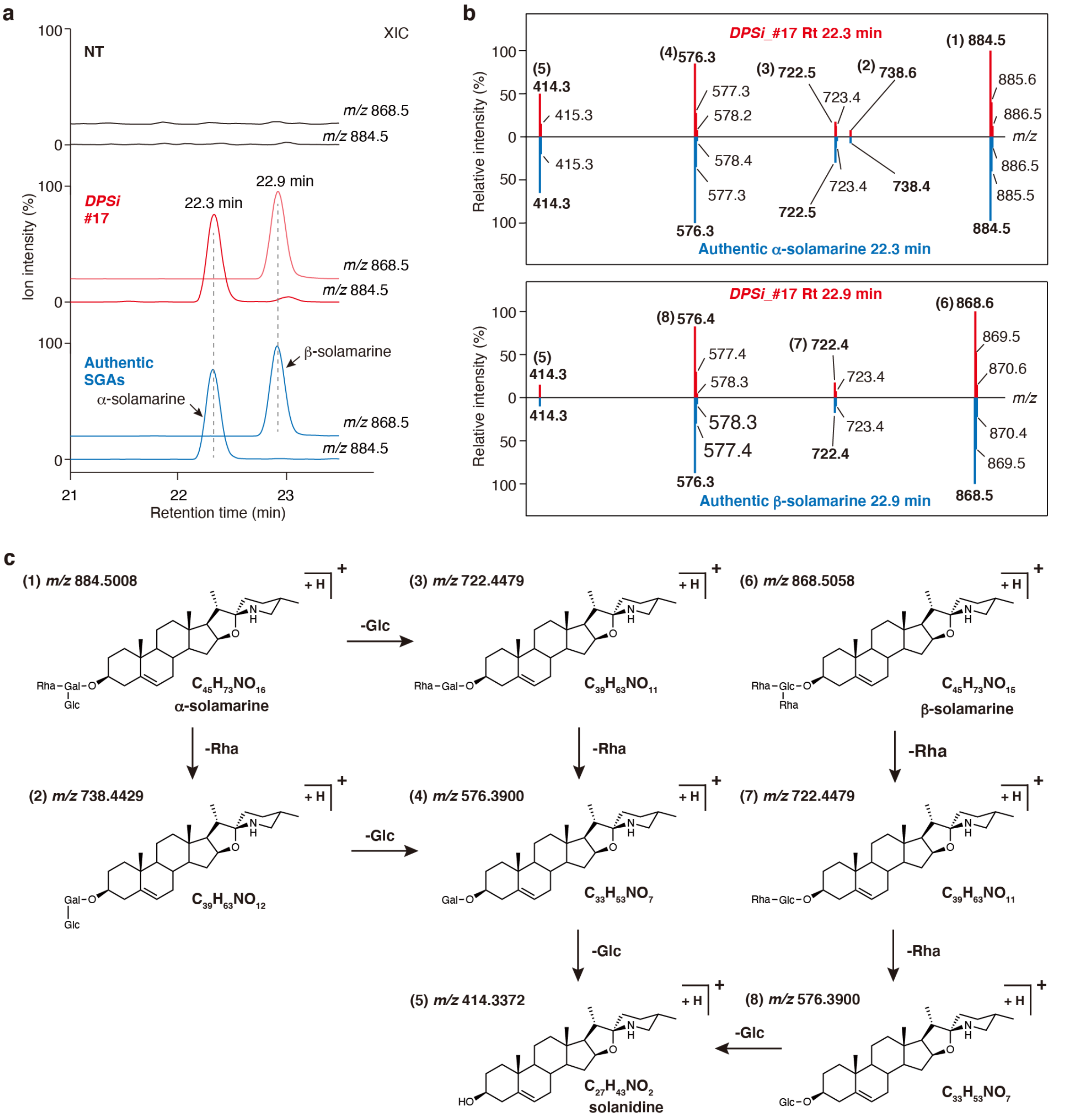

Supplementary Fig. 7| LC-MS characterization of accumulated SGAs in DPS-silenced transgenic potato plants. a Extracted ion chromatograms of *m/z* 884.5 $[M+H]^+$ and 868.5 $[M+H]^+$, parental mass of α-solamarine **7** and β-solamarine **8**, respectively. **b** ESI mass fragmentation spectra. Both MS data were obtained in positive ionization mode with a full-scan range of 350-1250 m/. The spectra verify that, despite their different origins, the products are chemically identical. All spectra were obtained from the center of the peaks. **c** MS fragmentation pathways for α-solamarine **7** and β-solamarine **8**. Abbreviations: NT, non-transformed control plants; XIC, extracted ion chromatogram; Rt, retention time; *m/z*, mass to charge; Glc, glucose; Gal, galactose; Rha, rhamnose.

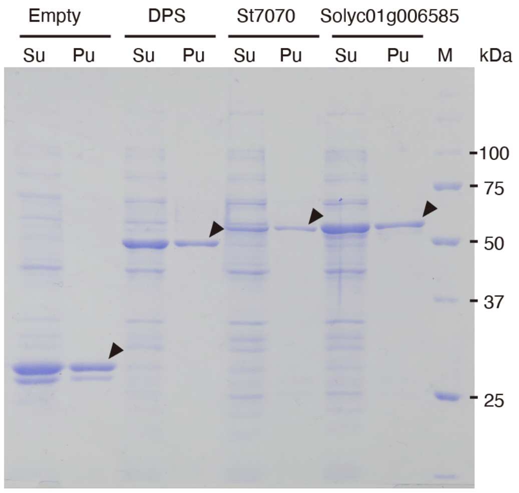

Supplementary Fig. 8| Heterologous expression of recombinant DPS, St7070, and Solyc01g006585 in *E. coli* BL21 (DE3), and purification using a cobalt column. SDS-PAGE analysis was used to confirm the expression and purification of the recombinant proteins. M: Molecular weight protein marker, Su: Supernatant fraction, Pu: Purified protein fraction. Empty: Empty pCold ProS2 vector transformed in to BL21 (DE3) cells used as a negative control. Purified recombinant proteins are marked with black triangles. The protein bands corresponding to the recombinant proteins (DPS, 31.4 kDa; St7070, 35.7 kDa; Solyc01g006585, 35.7 kDa) fused with ProS2 tag (23 kDa) were detected. Experiments were repeated three times with similar results.

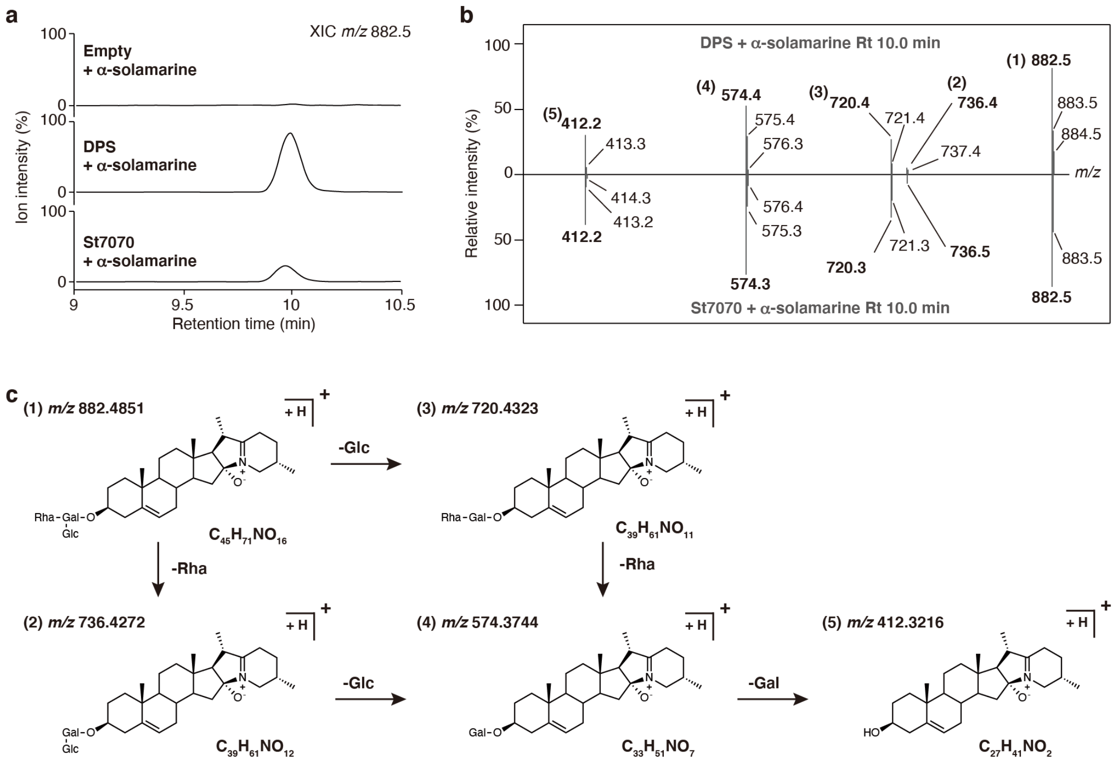

Supplementary Fig. 9| LC-MS analyses of the reaction products from the recombinant DPS and St7070 proteins with α-solamarine as a substrate. a Extracted ion chromatograms of *m/z* 882.5 $[M+H]^+$, parental mass of product of DPS enzymatic reaction using α-solamarine **7** as a substrate. Empty means a negative control reaction carried out with the proteins produced in *E. coli* transformed with an empty vector. **b** ESI mass fragmentation spectra. Both MS data were obtained in positive ionization mode with a full-scan range of 350-1250 *m/z*. The spectra verify that, despite their different origins, the products are chemically identical. All spectra were obtained from the center of the peaks. **c** MS fragmentation pathways for product of DPS enzymatic reaction using α-solamarine **7** as a substrate. Abbreviations: XIC, extracted ion chromatogram; Rt, retention time; *m/z*, mass to charge; Glc, glucose; Gal, galactose; Rha, rhamnose.

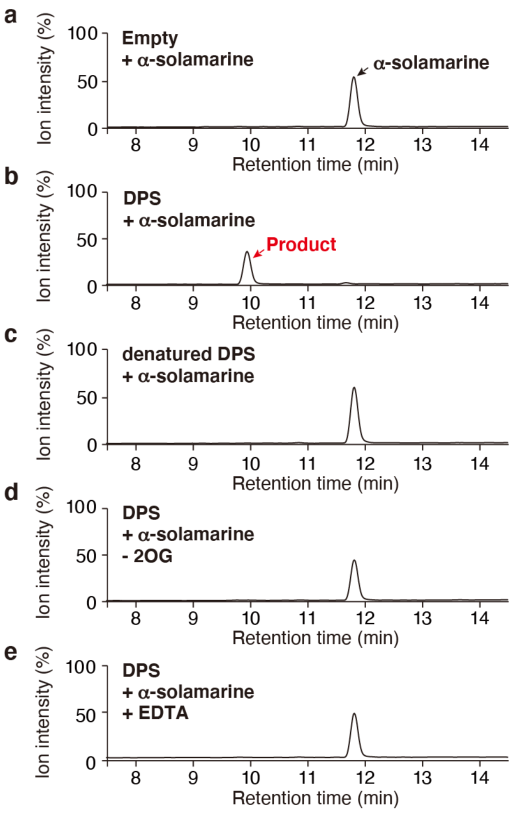

Supplementary Fig. 10| LC-MS analysis of DPS catalyzed *in vitro* reaction product using α-solamarine as a substrate in several conditions. a LC-MS chromatogram of the reaction carried out with the proteins produced in *E. coli* transformed with an empty vector. **b** LC-MS chromatogram of the product produced via the catalytic conversion of α-solamarine by DPS. **c** LC-MS chromatogram of the assay with boiled and denatured DPS enzyme. **d** LC-MS chromatogram of the assay in the absence of 2-oxoglutarate (2OG). **e** LC-MS chromatogram of the assay in the presence of ethylenediaminetetraacetic acid (EDTA) with the ratio (Fe^{2+} : EDTA = 1:1).

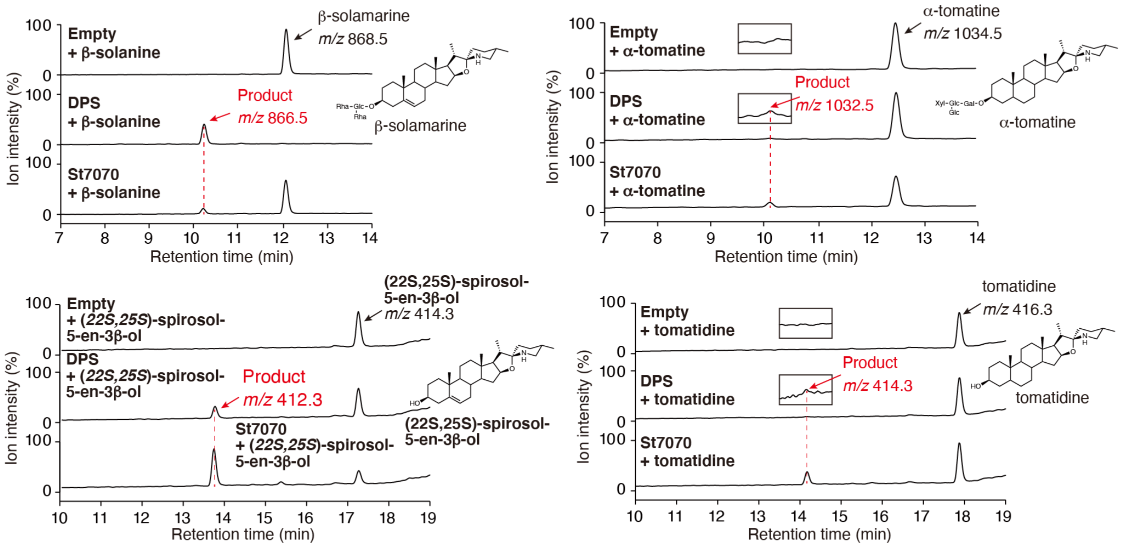

Supplementary Fig. 11| LC-MS analyses of the reaction products from the recombinant DPS and St7070 proteins with spirosolane substrates. Total ion current chromatogram obtained in positive ionization mode with a full-scan range of 350-1250 m/z are shown. Empty means a negative control reaction performed using a purified protein fraction from *E. coli* cells transformed with an empty vector. Mass to charge (*m/z*) is shown for the substrate and the reaction product. MS spectra and fragmentation patterns are provided in Supplementary Figs. 12-15

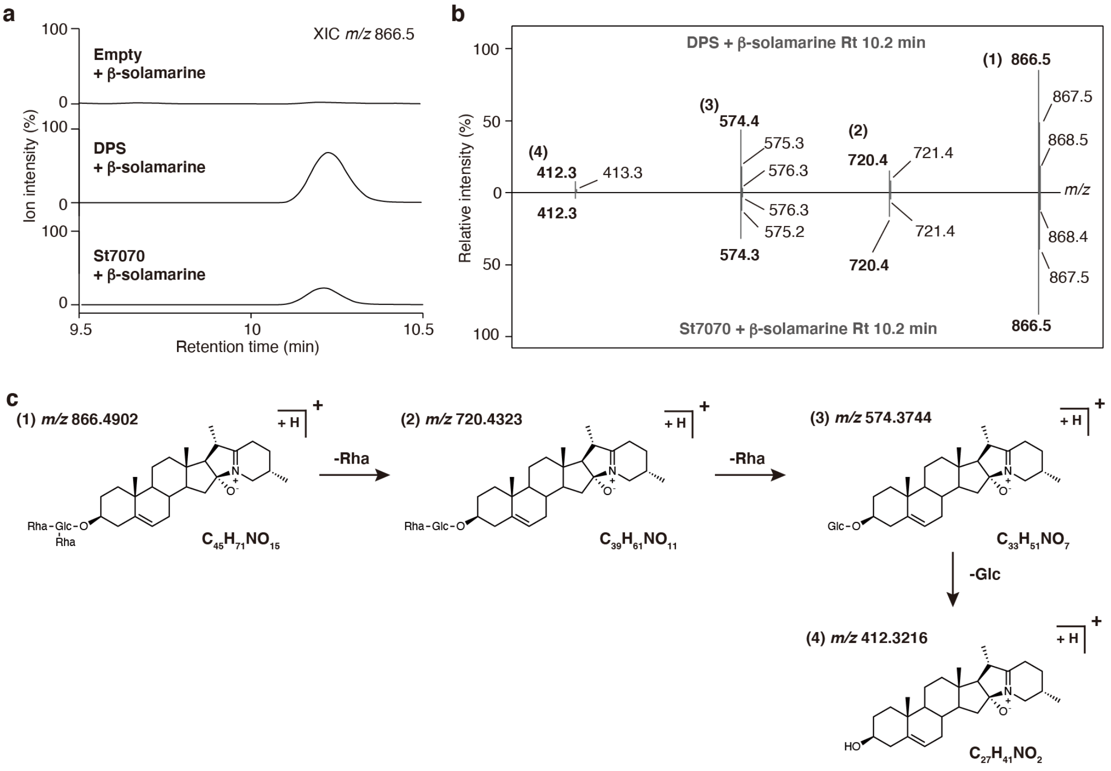

Supplementary Fig. 12| LC-MS analyses of the reaction products from the recombinant DPS and St7070 proteins with β-solamarine as a substrate. a Extracted ion chromatograms of *m/z* 866.5 $[M+H]^+$, parental mass of product of DPS enzymatic reaction using β-solamarine **8** as a substrate. Empty means a negative control reaction carried out with the proteins produced in *E. coli* transformed with an empty vector. **b** ESI mass fragmentation spectra. Both MS data were obtained in positive ionization mode with a full-scan range of 350-1250 *m/z*. The spectra verify that, despite their different origins, the products are chemically identical. All spectra were obtained from the center of the peaks. **c** MS fragmentation pathways for the product of DPS enzymatic reaction using β-solamarine **8** as a substrate. Abbreviations: XIC, extracted ion chromatogram; Rt, retention time; *m/z*, mass to charge; Glc, glucose; Rha, rhamnose.

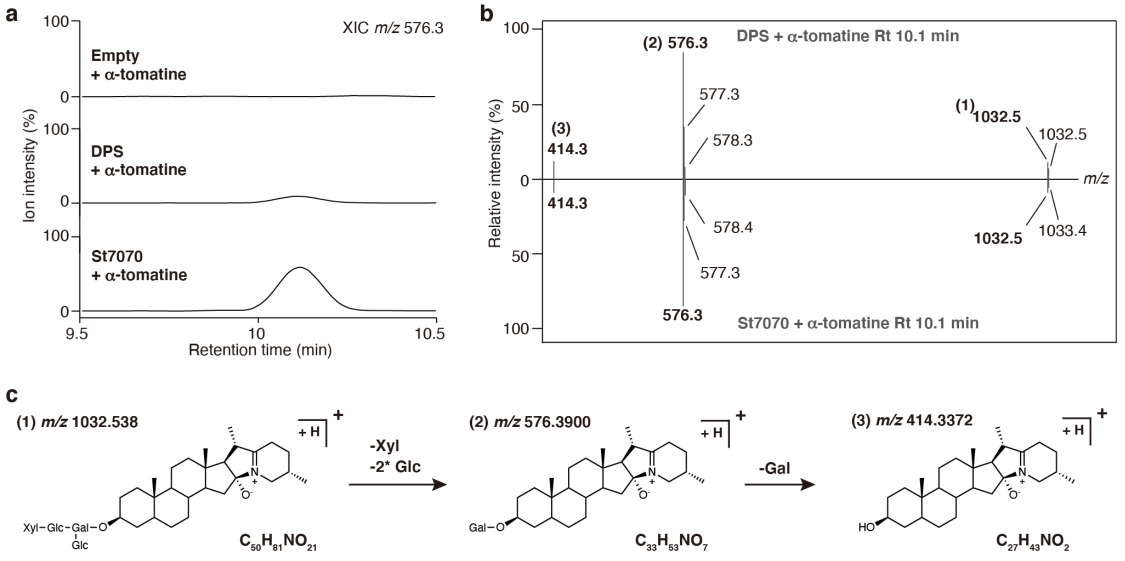

Supplementary Fig. 13| LC-MS analyses of the reaction products from the recombinant DPS and St7070 proteins with α-tomatine as a substrate. a Extracted ion chromatograms of *m/z* 576.3 [M+H-Xyl-2*Glc]$^{+}$, fragment mass of product of DPS enzymatic reaction using α-tomatine **5** as a substrate. Empty means a negative control reaction carried out with the proteins produced in *E. coli* transformed with an empty vector. **b** ESI mass fragmentation spectra. Both MS data were obtained in positive ionization mode with a full-scan range of 350-1250 *m/z*. The spectra verify that, despite their different origins, the products are chemically identical. All spectra were obtained from the center of the peaks. **c** MS fragmentation pathways for product of DPS enzymatic reaction using α-tomatine **5** as a substrate. Abbreviations: XIC, extracted ion chromatogram; Rt, retention time; *m/z*, mass to charge; Glc, glucose; Rha, rhamnose; Xyl, xylose.

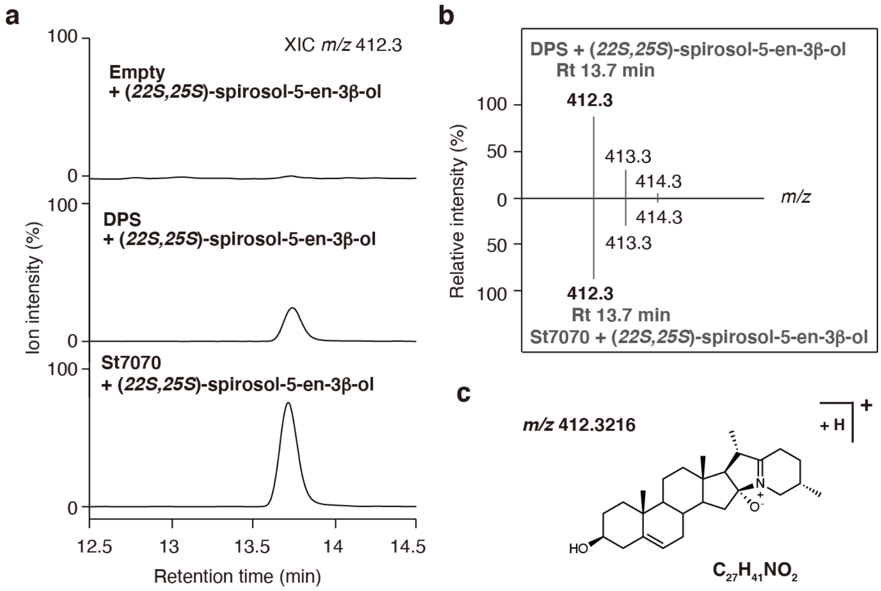

Supplementary Fig. 14| LC-MS analyses of the reaction products from the recombinant DPS and St7070 proteins with (*22S*,*25S*)-spirosol-5-en-3β-ol as a substrate. **a** Extracted ion chromatograms of *m/z* 412.3 $[M+H]^+$, parental mass of the product of DPS enzymatic reaction using (*22S*,*25S*)-spirosol-5-en-3β-ol **3** as a substrate. Empty means a negative control reaction carried out with the proteins produced in *E. coli* transformed with an empty vector. **b** ESI mass fragmentation spectra. Both MS data were obtained in positive ionization mode with a full-scan range of 350-450 *m/z*. The spectra verify that, despite their different origins, the products are chemically identical. All spectra were obtained from the center of the peaks. **c** MS fragment of the product of DPS enzymatic reaction using (*22S*,*25S*)-spirosol-5-en-3β-ol **3** as a substrate. Abbreviations: XIC, extracted ion chromatogram; Rt, retention time; *m/z*, mass to charge.

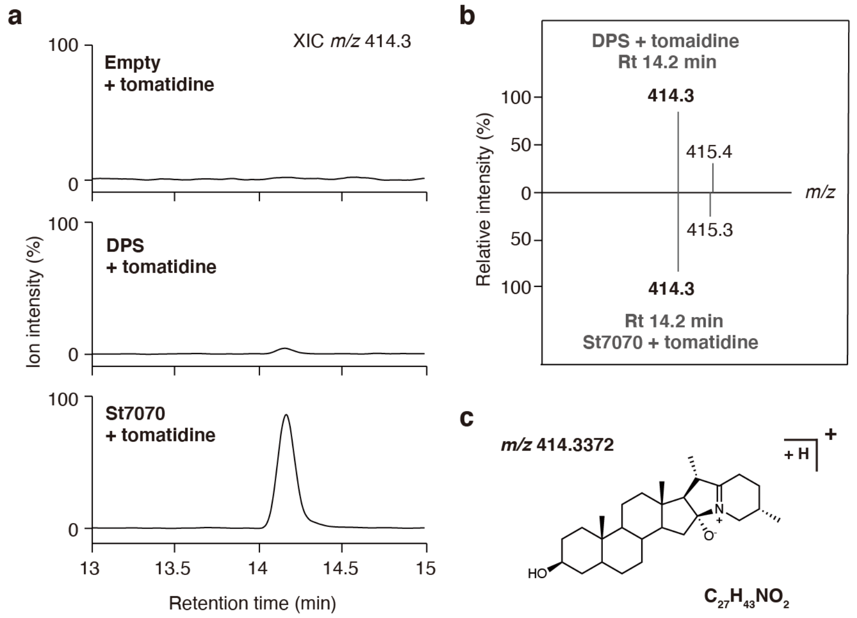

Supplementary Fig. 15| LC-MS analyses of the reaction products from the recombinant DPS and St7070 proteins with tomatidine as a substrate. a Extracted ion chromatograms of *m/z* 412.3 $[M+H]^+$, parental mass of the product of DPS enzymatic reaction using tomatidine **4** as a substrate. Empty means a negative control reaction carried out with the proteins produced in *E. coli* transformed with an empty vector. **b** ESI mass fragmentation spectra. Both MS data were obtained in positive ionization mode with a full-scan range of 350-450 *m/z*. The spectra verify that, despite their different origins, the products are chemically identical. All spectra were obtained from the center of the peaks. **c** MS fragment of the product of DPS enzymatic reaction using tomatidine **4** as a substrate. Abbreviations: XIC, extracted ion chromatogram; Rt, retention time; *m/z*, mass to charge.

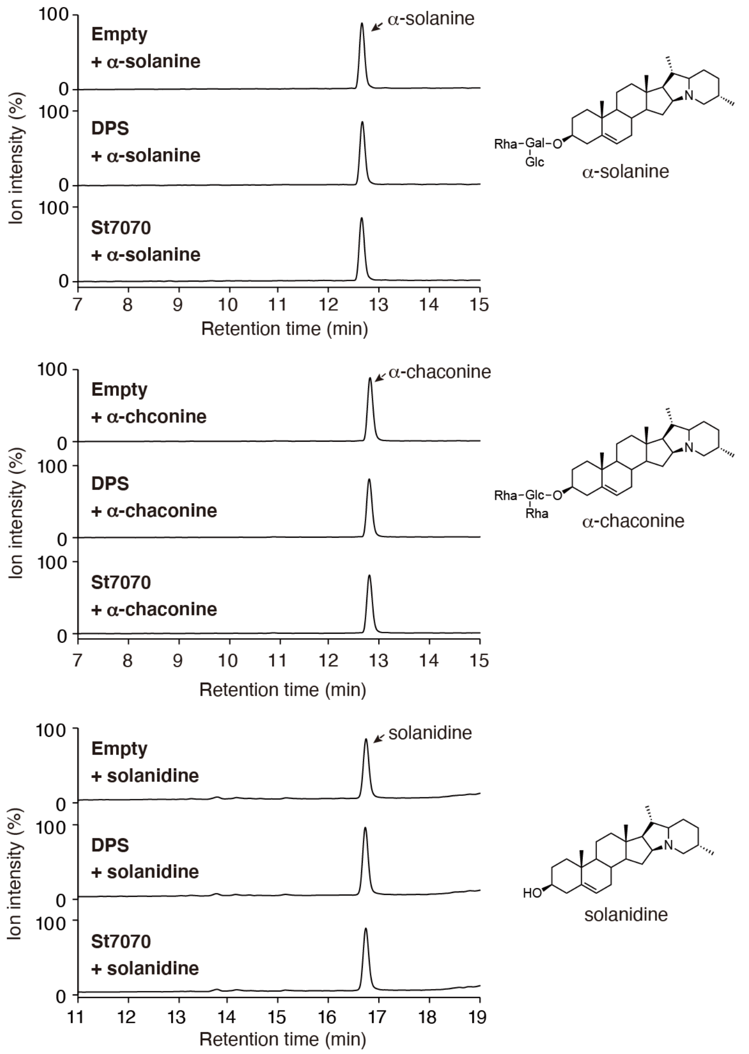

Supplementary Fig. 16| LC-MS analyses of the reaction products from the recombinant DPS and St7070 with solanidane substrates. Total ion current chromatogram obtained in positive ionization mode with a full-scan range of 350-1250 m/z are shown. Empty means a negative control reaction performed using a purified protein fraction from *E. coli* cells transformed with an empty vector.

[Molecular Formula]
Elements : C 50/50, H 82/80, N 1/1, O 22/21, Na 1/0
Mass Tolerance : 5ppm
Unsaturation (U.S.) : -100.0 - 100.0

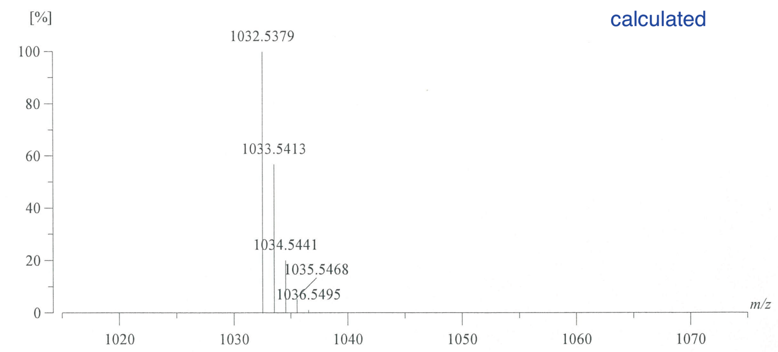

[Mass Spectrum]
Data : FAB_114 Date : 22-Mar-2018 15:30
Instrument : MStation JMS-MS700V
Sample : DOX130 (H2O)
Note : Matrix: Glycerol Calibrant: ULT, dual-target
Inlet : Direct Ion Mode : FAB+
Scan# : (8,10)

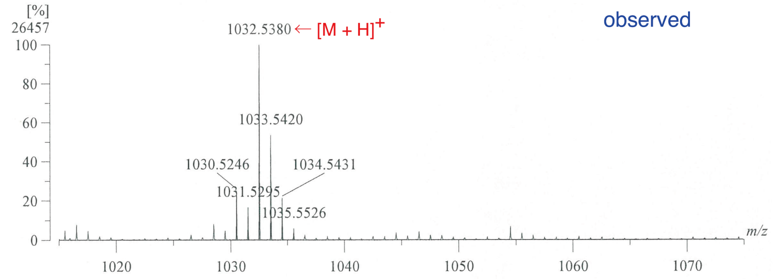

	Observed m/z	Int%	Err[ppm / mmu]	U.S.	Composition
1	1030.5246	25.18	+2.2 / +2.3	11.5	C50 H80 N O21
2	1031.5295	16.65	-0.6 / -0.6	11.0	C50 H81 N O21
3	1032.5380	100.00	+0.1 / +0.1	10.5	C50 H82 N O21 ← $[M + H]^+$
	1033.5420	53.66			
	1034.5431	21.51			
	1035.5526	5.96			

Supplementary Fig. 17| High resolution mass spectrometric analysis of DPS enzymatic reaction product.

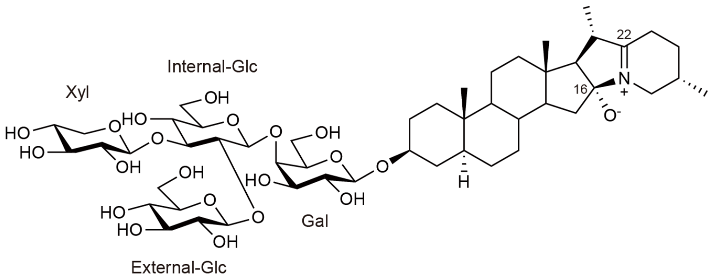

Supplementary Fig. 18| The structure of the DPS enzymatic reaction product, as confirmed by HRMS and NMR analyses. Abbreviations: Glc, glucose; Gal, galactose; Xyl, xylose

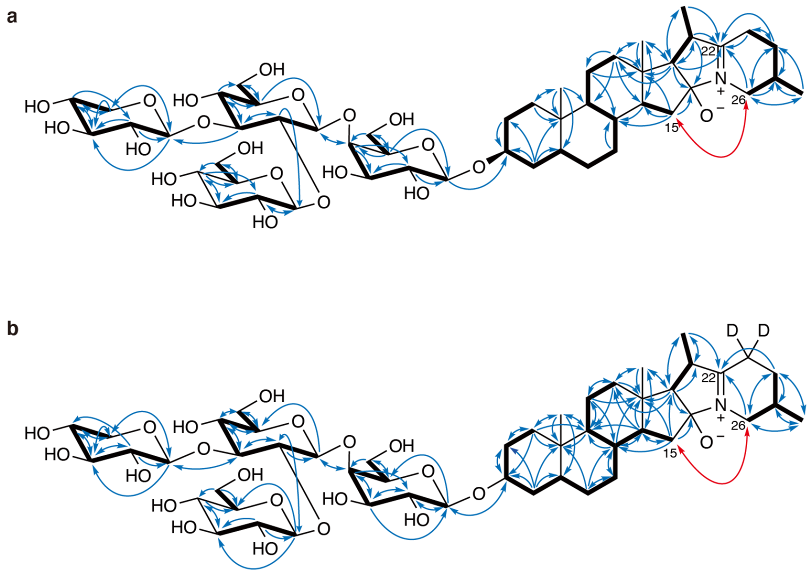

Supplementary Fig. 19| COSY (bold bond), HMBC (blue arrow), and key NOE (red arrow) correlations of the DPS product in CD_3SOCD_3 (a) and CD_3OD (b). The configuration is omitted for clarity. Abbreviations: COSY, correlation spectroscopy; HMBC, heteronuclear multiple-bond correlation spectroscopy; NOE, nuclear Overhauser effect.

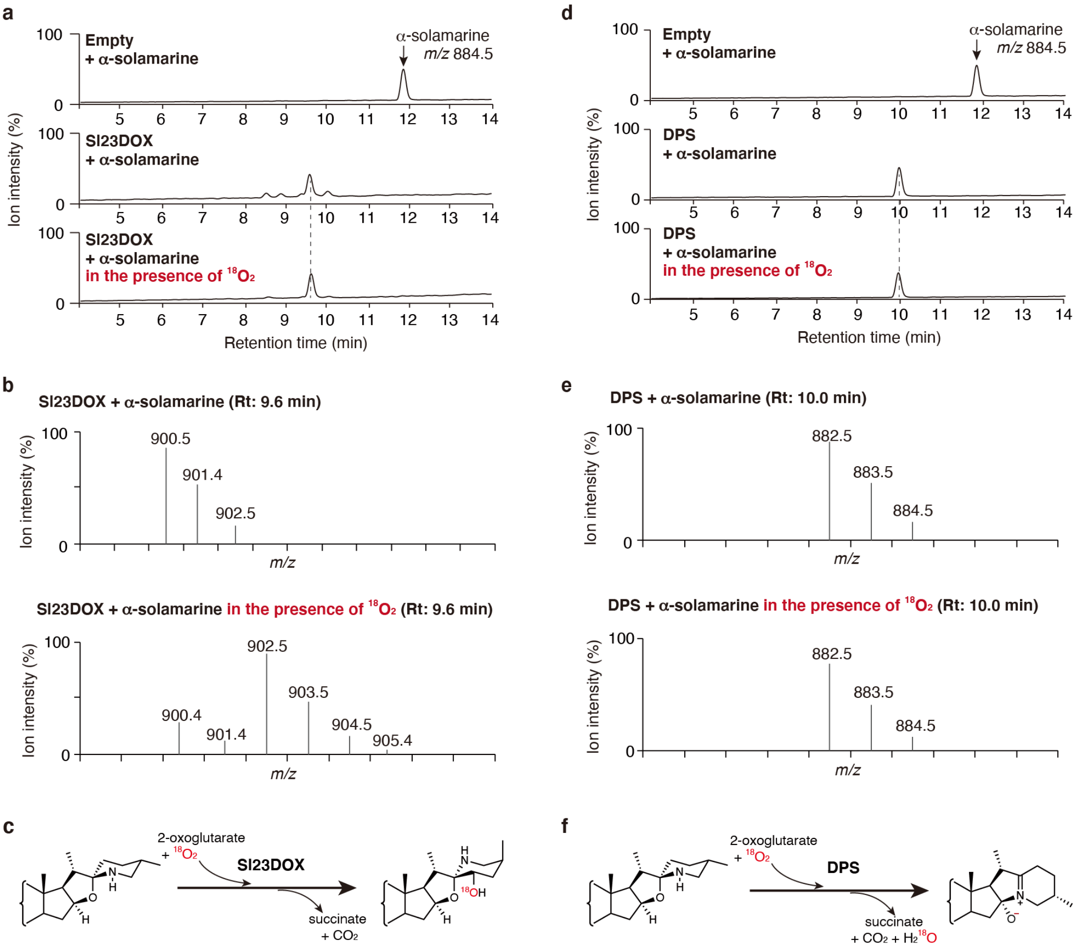

Supplementary Fig. 20| In vitro enzyme activity of Sl23DOX and DPS in the presence of $^{18}O_2$. **a** LC-MS total ion current chromatogram of the enzyme assays of Sl23DOX with α-solamarine **7** as a substrate under normal atmosphere conditions or under an $^{18}O_2$ atmosphere. **b** MS-spectra of hydroxylated α-solamarine showing incorporation of ^{18}O. **c** Reaction catalyzed by Sl23DOX in the presence of $^{18}O_2$. **d** LC-MS total ion current chromatograms of the enzyme assays of DPS with α-solamarine **7** as a substrate under normal atmosphere conditions or under an $^{18}O_2$ atmosphere. **e** DPS enzymatic reaction product. **c** Reaction catalyzed by DPS in the presence of $^{18}O_2$. Abbreviation: *m/z*, mass to charge.

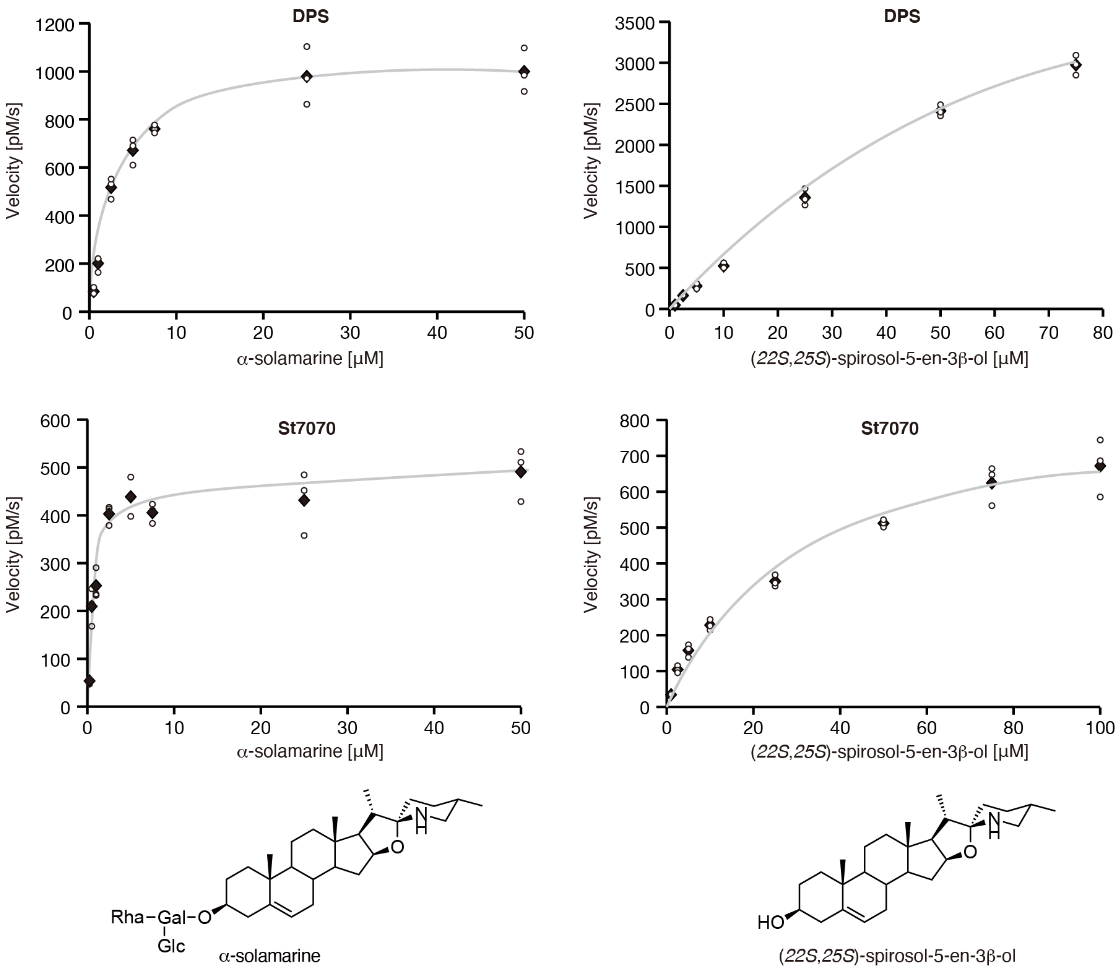

Supplementary Fig. 21| Enzyme kinetics of purified recombinant DPS and St7070 with α-solamarine and (*22S*,*25S*)-spirosol-5-en-3β-ol. Purified recombinant enzymes of DPS and St7070 were assayed using different concentrations of α-solamarine **7** (up to 75 μM) or (*22S*,*25S*)-spirosol-5-en-3β-ol (up to 100 μM) and a fixed concentration of 2-oxoglutrate (100 mM). A Michalis-Menten curve was fitted to the values obtained and the calculated kinetic parameters are showed in Supplementary Table 4. Error bars represent ± standard error of mean (n = 3).

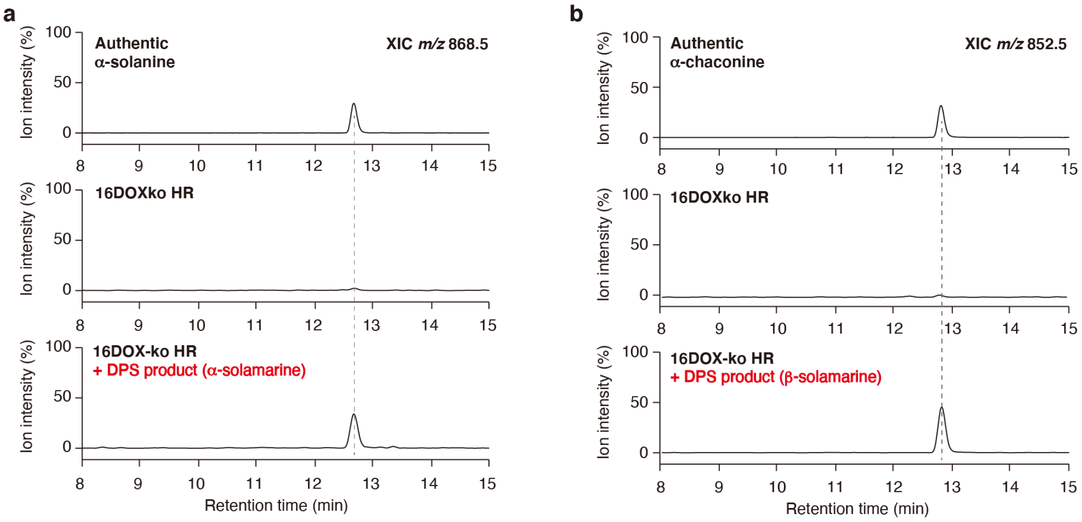

Supplementary Fig. 22| Bioconversion of DPS enzymatic reaction product in 16DOX-disrupted potato hairy roots. **a** Administration of the product from the DPS enzymatic reaction using α-solamarine **7** as a substrate to 16DOX-disrupted potato hairy roots. XICs correspond to parental mass of α-solanine **9**, *m/z* 868.5 $[M+H]^+$. **a** Administration of the product from the DPS enzymatic reaction using β-solamarine **8** as a substrate to 16DOX-disrupted potato hairy roots. 16DOXko HR 16DOX-disrupted potato hairy roots, XICs correspond to parental mass of α-chaconine **10**, *m/z* 852.5 $[M+H]^+$. Abbreviations: XIC, extracted ion chromatogram; Rt, retention time; *m/z*, mass to charge.

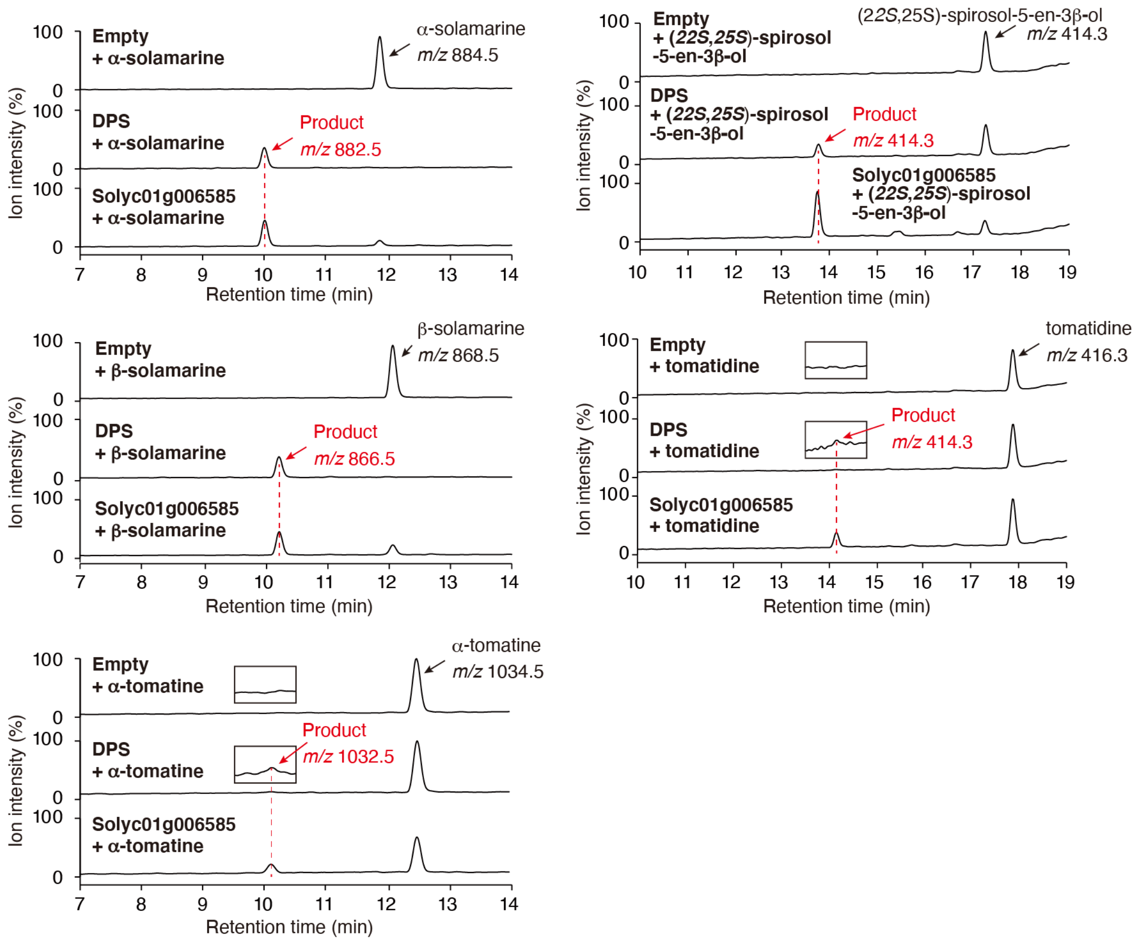

Supplementary Fig. 23| LC-MS analyses of products from the reactions of recombinant Solyc01g006585 proteins with various spirosolane substrates. Empty means a negative control reaction performed using a purified protein fraction from *E. coli* cells transformed with an empty vector. Mass to charge (*m/z*) is shown for substrate and reaction product. MS spectra of each product are provided in Supplementary Figs. 23-27

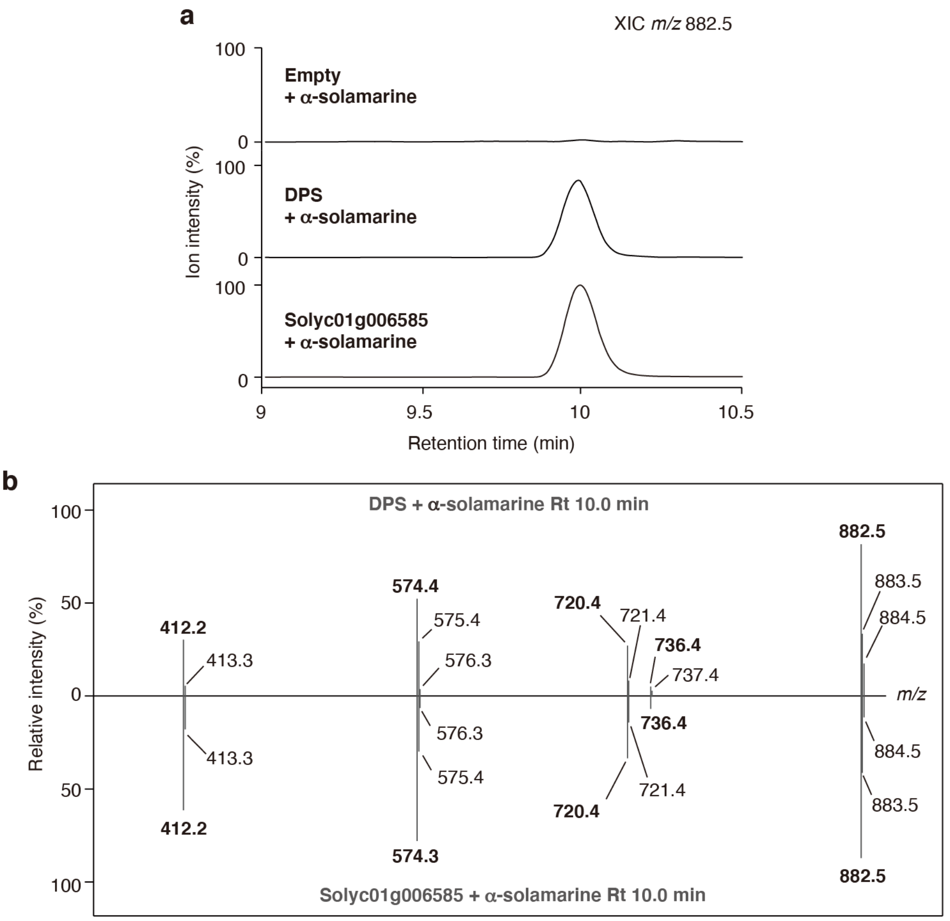

Supplementary Fig. 24| LC-MS analyses of the reaction products from the recombinant DPS and Solyc01g006585 proteins with α-solamarine as a substrate. a Extracted ion chromatograms of *m/z* 882.5 $[M+H]^+$, parental mass of the product of the DPS enzymatic reaction using α-solamarine **7** as a substrate. Empty means a negative control reaction carried out with the proteins produced in *E. coli* transformed with an empty vector. **b** ESI mass fragmentation spectra. Both MS data were obtained in positive ionization mode with a full-scan range of 350-1250 *m/z*. The spectra verify that, despite their different origins, the products are chemically identical. All spectra were obtained from the center of the peaks. Abbreviations: XIC, extracted ion chromatogram; Rt, retention time; *m/z*, mass to charge.

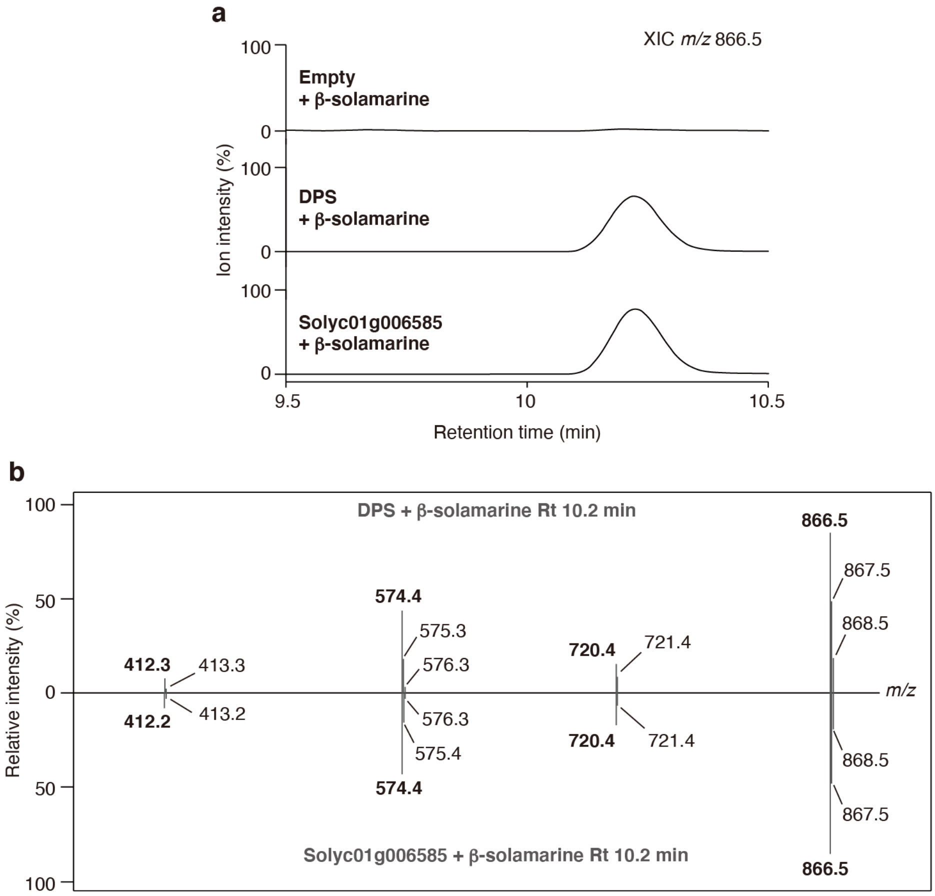

Supplementary Fig. 25| LC-MS analyses of the reaction products from the recombinant DPS and Solyc01g006585 proteins with β-solamarine as a substrate. a Extracted ion chromatograms of *m/z* 866.5 $[M+H]^+$, parental mass of the product of the DPS enzymatic reaction using β-solamarine **8** as a substrate. Empty means a negative control reaction carried out with the proteins produced in *E. coli* transformed with an empty vector. **b** ESI mass fragmentation spectra. Both MS data were obtained in positive ionization mode with a full-scan range of 350-1250 *m/z*. The spectra verify that, despite their different origins, the products are chemically identical. All spectra were obtained from the center of the peaks. Abbreviations: XIC, extracted ion chromatogram; Rt, retention time; *m/z*, mass to charge; Glc, glucose; Rha, rhamnose.

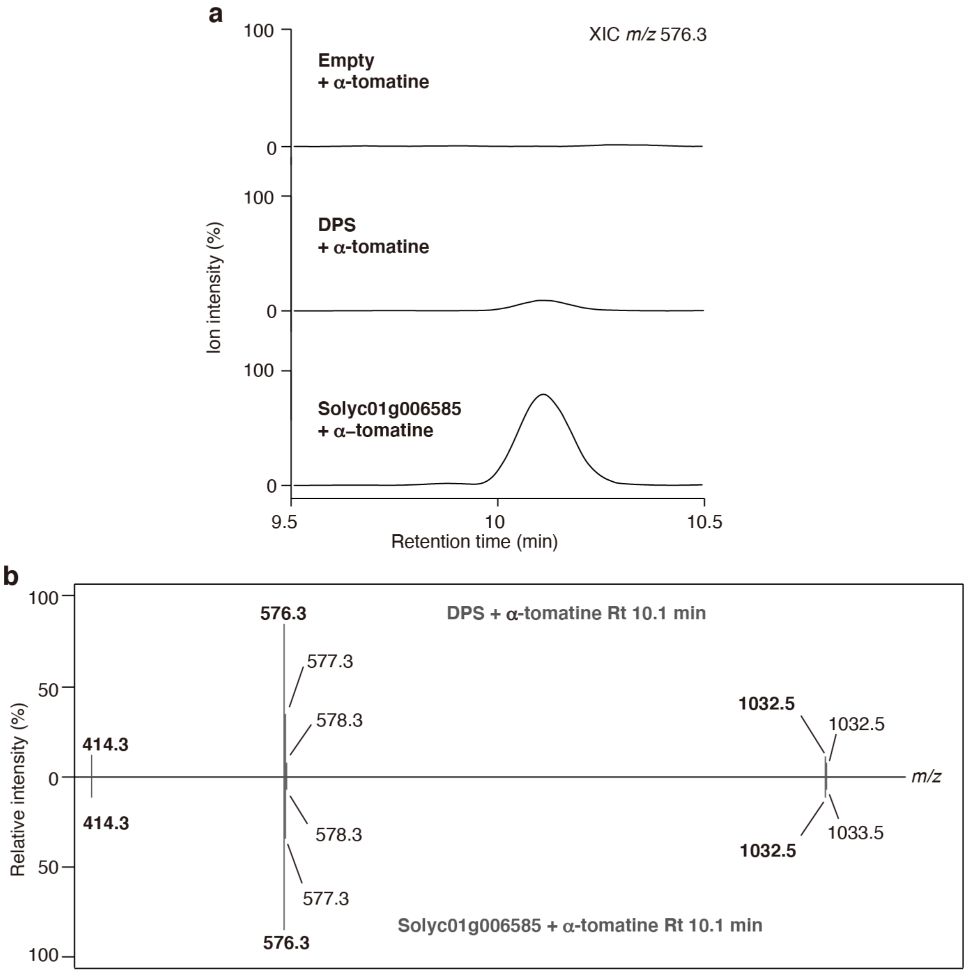

Supplementary Fig. 26| LC-MS analyses of the reaction products from the recombinant DPS and Solyc01g006585 proteins with α-tomatine as a substrate. a Extracted ion chromatograms of *m/z* 576.3 [M+H-Xyl-2*Glc]$^+$, fragment mass of the product of the DPS enzymatic reaction using α-tomatine **5** as a substrate. Empty means a negative control reaction carried out with the proteins produced in *E. coli* transformed with an empty vector. **b** ESI mass fragmentation spectra. Both MS data were obtained in positive ionization mode with a full-scan range of 350-1250 *m/z*. The spectra verify that, despite their different origins, the products are chemically identical. All spectra were obtained from the center of the peaks. Abbreviations: XIC, extracted ion chromatogram; Rt, retention time; *m/z*, mass to charge.

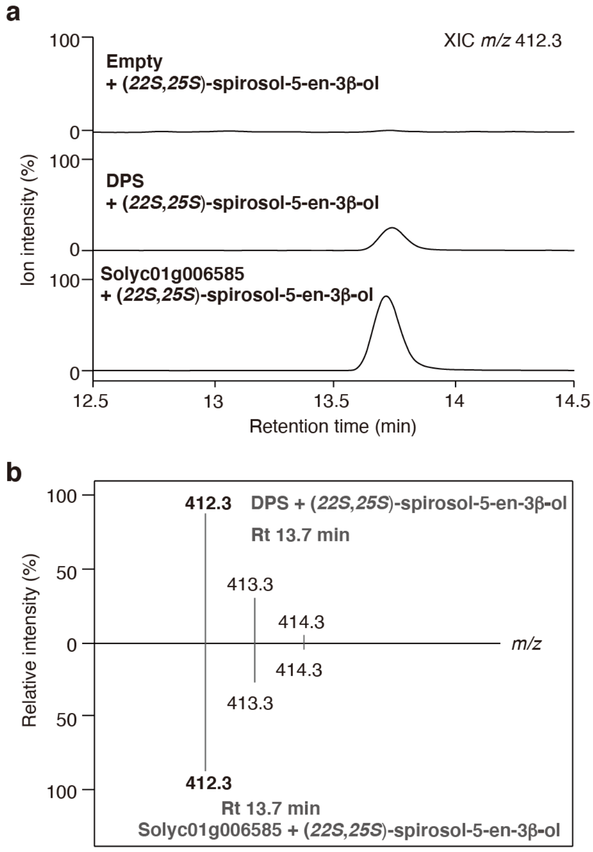

Supplementary Fig. 27| LC-MS analyses of the reaction products from the recombinant DPS and Solyc01g006585 proteins with (*22S*,*25S*)-spirosol-5-en-3β-ol as a substrate. a Extracted ion chromatograms of *m/z* 412.3 $[M+H]^+$, parental mass of product of DPS enzymatic reaction using (*22S*,*25S*)-spirosol-5-en-3β-ol **3** as a substrate. Empty means a negative control reaction carried out with the proteins produced in *E. coli* transformed with an empty vector. **b** ESI mass fragmentation spectra. Both MS data were obtained in positive ionization mode with a full-scan range of 350-450 *m/z*. The spectra verify that, despite their different origins, the products are chemically identical. All spectra were obtained from the center of the peaks. Abbreviations: XIC, extracted ion chromatogram; Rt, retention time; *m/z*, mass to charge.

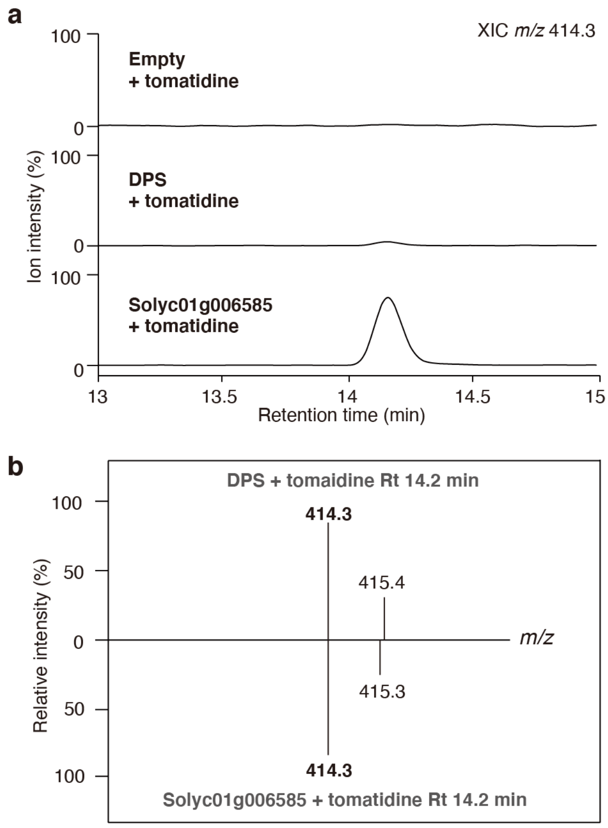

Supplementary Fig. 28| LC-MS analyses of the reaction products from the recombinant DPS and Solyc01g006585 proteins with tomatidine as a substrate. a Extracted ion chromatograms of *m/z* 412.3 $[M+H]^+$, parental mass of the product of the DPS enzymatic reaction using tomatidine **4** as a substrate. Empty means a negative control reaction carried out with the proteins produced in *E. coli* transformed with an empty vector. **b** ESI mass fragmentation spectra. Both MS data were obtained in positive ionization mode with a full-scan range of 350-450 *m/z*. The spectra verify that, despite their different origins, the products are chemically identical. All spectra were obtained from the center of the peaks. Abbreviations: XIC, extracted ion chromatogram; Rt, retention time; *m/z*, mass to charge.

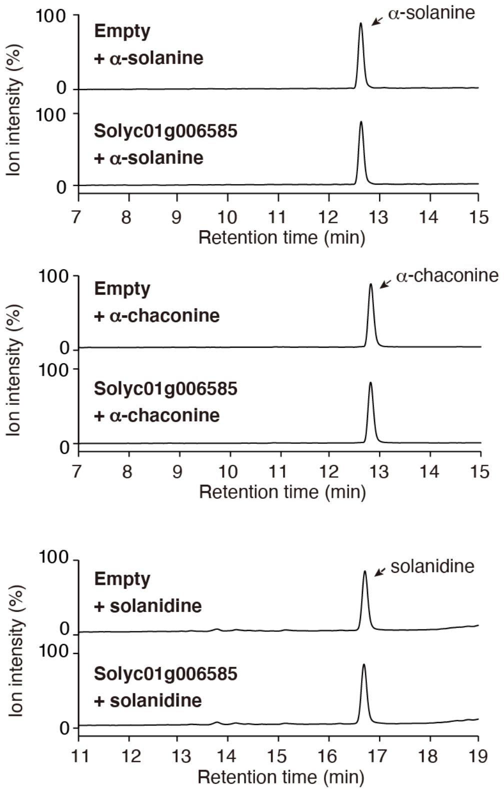

Supplementary Fig. 29| LC-MS analyses of the reaction products from the recombinant DPS and Solyc01g006585 with solanidane substrates. Total ion current chromatogram obtained in positive ionization mode with a full-scan range of 350-1250 m/z are shown. Empty means a negative control reaction performed using a purified protein fraction from *E. coli* cells transformed with an empty vector.

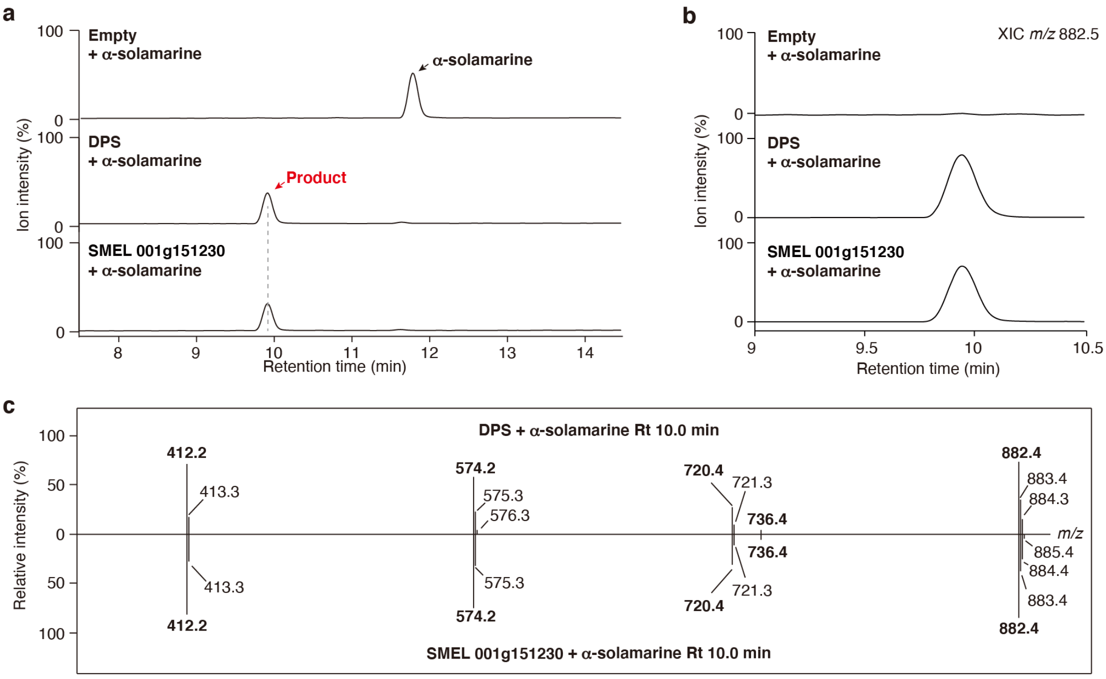

Supplementary Fig. 30| LC-MS analyses of the reaction products from the recombinant DPS and SMEL 001g151230 with α-solamarine as a substrate. Total ion current chromatogram obtained in positive ionization mode with a full-scan range of 350-1250 m/z are shown. Empty means a negative control reaction performed using a purified protein fraction from *E. coli* cells transformed with an empty vector.

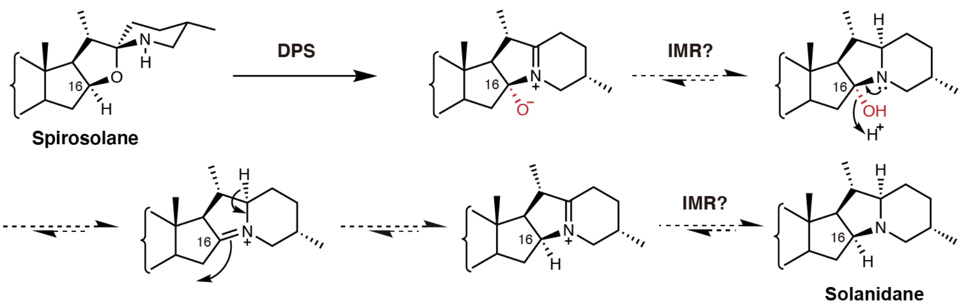

Supplementary Fig. 31| Putative biosynthetic pathway to furnish solanidane skeleton. Putative imine reductase (IMR) reduces the iminium moiety of the DPS product to give hemiaminal. Then, the lone pair on the nitrogen atom pushes the hydroxy group at C-16 to leave as water to form iminium species, which would isomerize. Finally, a putative IMR again reduces iminium moiety to furnish solanidane.

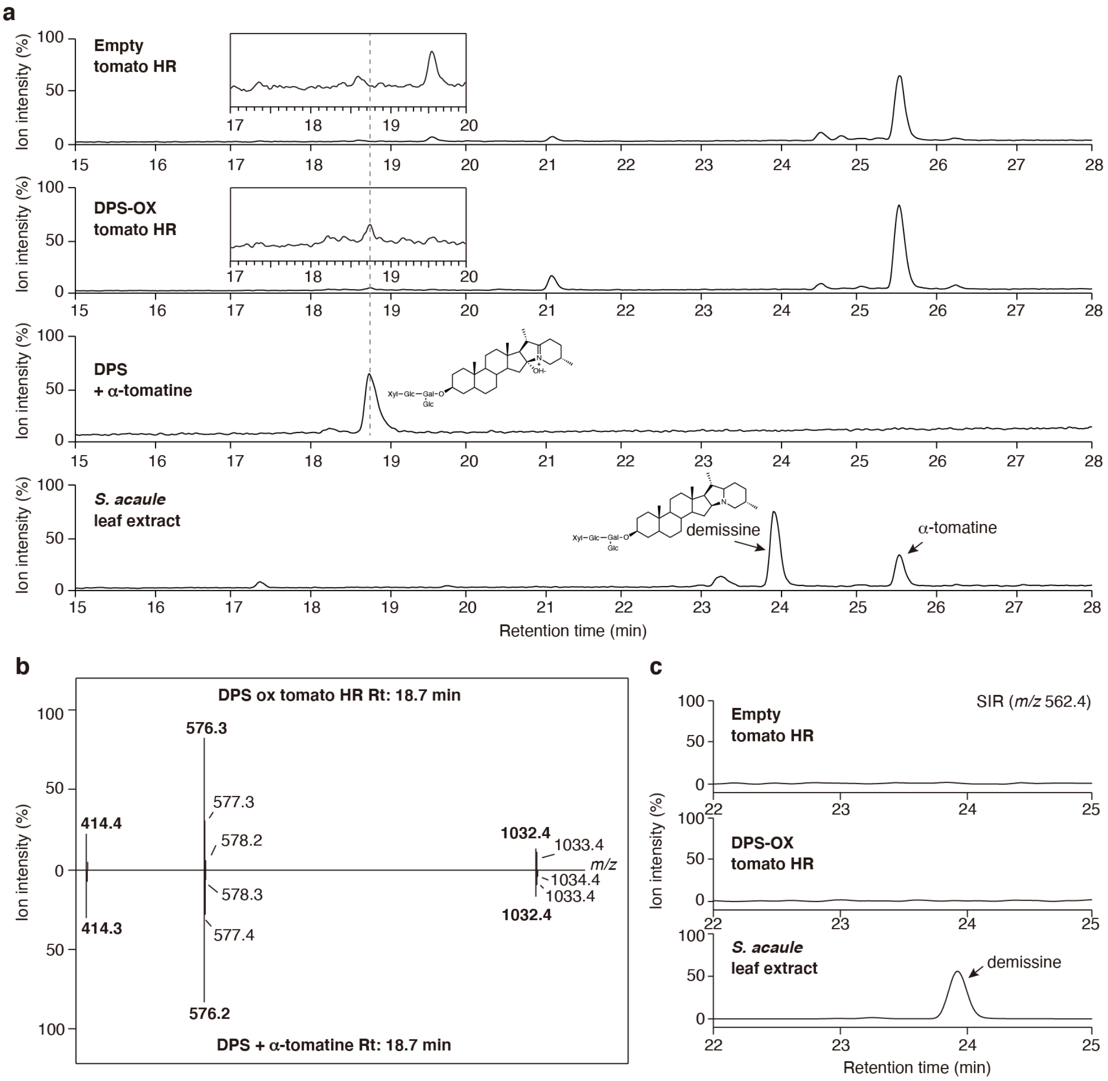

Supplementary Fig. 32| LC-MS analysis of accumulated SGAs in DPS over-expressing tomato hairy roots. a Total ion current chromatogram obtained in positive ionization mode with a full-scan range of 350-1250 *m/z*. **b** ESI mass spectra of peaks at a retention time of 18.7 min. The spectra verify that, despite their different origins, the products are chemically identical. All spectra were obtained from the center of the peaks. **c** Selected ion recording (SIR) chromatograms of *m/z* 562.5 $[M+H\text{-}Xyl\text{-}2*Glc]^+$, major fragment mass of demissine **11**. Abbreviations: HR, hairy roots; SIR, selected ion recording; Rt, retention time; *m/z*, mass to charge; Glc, glucose; Gal, galactose; Xyl, xylose.

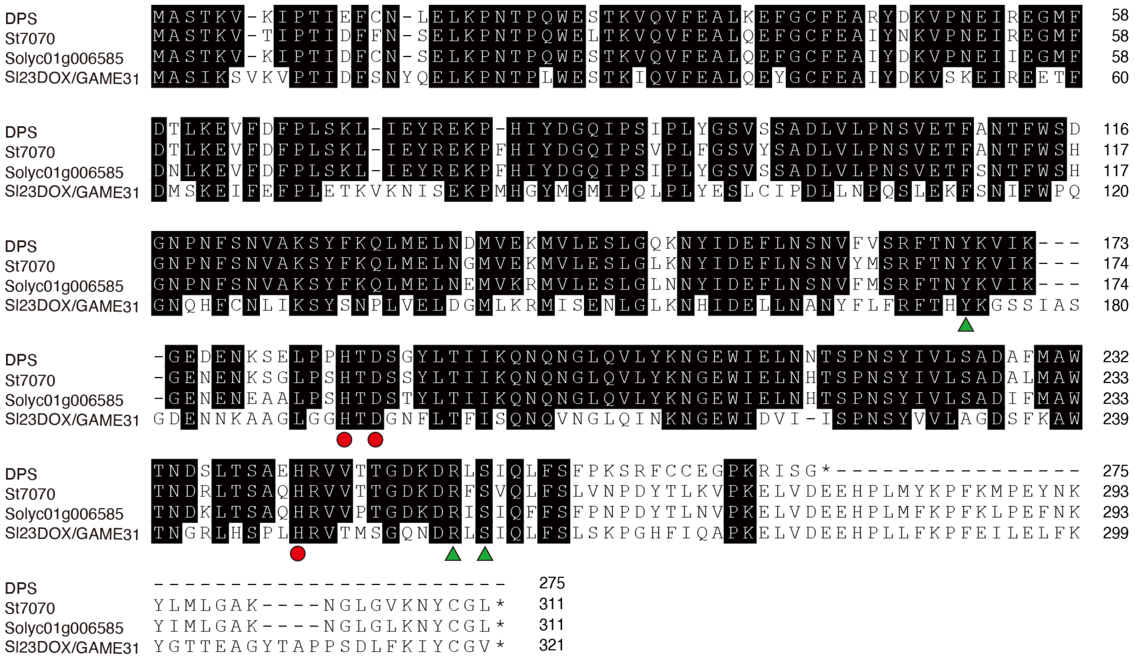

Supplementary Fig. 33| Alignment of the amino acid sequences of DPS, St7070, Solyc01g006585, and Sl23DOX/GAME31. Sequences were aligned using Clustal Omega. Shaded black boxes indicate residues that are identical in at least DPS, St7070, and Solyc01g006585. The catalytic residues/motifs conserved in the DOX family are marked as follows: a canonical HXDXnH catalytic triad (red circles) required to coordinate Fe (II); a YXnRXS motif (green triangles) implicated in 2-oxoglutrate binding.

```
DPS     TCCTATATTGTTTTATCAGCAGATGCTTTCATGGCATGGACAAATGATAGTTTGACATCT   717
         S  Y  I  V  L  S  A  D  A  F  M  A  W  T  N  D  S  L  T  S  239
         S  Y  I  V  L  S  A  D  A  L  M  A  W  T  N  D  R  L  T  S  240
St7070  TCCTATATTGTTTTATCAGCAGATGCTCTTATGGCATGGACAAATGATAGATTGACATCT   720

DPS     GCTGAACACAGAGTAGTAACAACAGGAGACAAAGATAGATTATCTATTCAATTATTTTCC   777
         A  E  H  R  V  V  T  T  G  D  K  D  R  L  S  I  Q  L  F  S  259
         A  Q  H  R  V  V  T  T  G  D  K  D  R  F  S  V  Q  L  F  S  260
St7070  GCTCAACATAGGGTTGTAACAACAGGAGACAAAGATAGATTCTCTGTTCAATTATTTTCC   780

DPS     TTTCCCAAATCAAGATTTTGCTGTGAAGGTCCCAAAAGAATTAGTGGATGAAGACCACCC   837
         F  P  K  S  R  F  C  C  E  G  P  K  R  I  S  G stop          275
         L   V  N  P  D  Y  T  L  K  V  P  K  E  L  V  D  E  E  H  P 280
St7070  CTC-GTAAATCCAGATTATACTTTGAAGGTCCCAAAAGAATTAGTGGATGAAGAACACCC   839

DPS     TTTACTCTTCAAGCCTTTTAACTTGCTTGAATTTCCTAAATAAACTATGTCAGGTGATAA   897

          L  M  Y  K  P  F  K  M  P  E  Y  N  K  Y  L  M  L  G  A  K   300
St7070  TTTAATGTACAAGCCTTTTAAGATGCCTGAATATAATAAATATCTTATGTTAGGTGCTAA   899

DPS     AAATTGAATTAATCTCAAGAATTATTGTGGTCTTTAA     934

          N   G  L  G  V  K  N  Y  C  G  L Stop  311
St7070  AAATGGATTGGGTGTCAAGAATTATTGTGGTCTTTAA     936
```

Supplementary Fig. 34| Nucleotide sequence alignment of C-terminus sequences of *DPS* and *St7070*. Shaded in yellow is the inserted nucleotide base that resulted in the frame shift of the DPS protein.

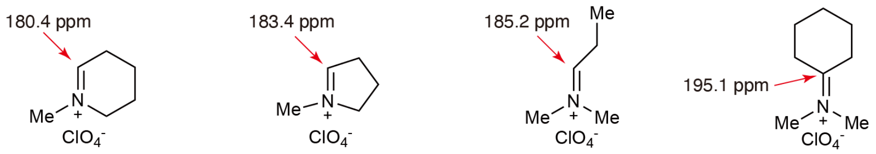

Supplementary Fig. 35| ^{13}C chemical shift of the carbon atom in iminium cations (ppm, CD_3NO_2, TMS).

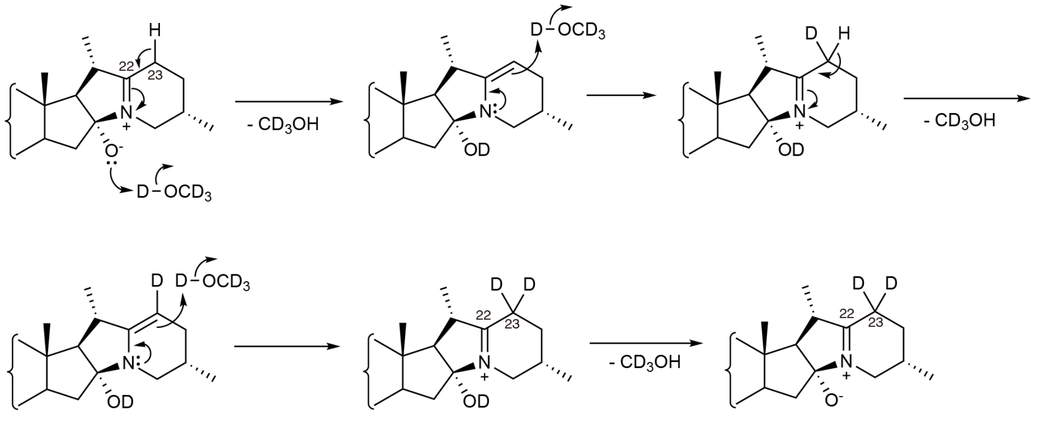

Supplementary Fig. 36| Putative mechanism for the H-D exchange of the H-22 from the DPS product in CD_3OD.

Supplementary Table 1| The list of the *DOX* genes highly expressed in tuber sprouts. The list includes the top 30 genes exhibiting high levels of expression in tuber sprouts. The FPKM values of the *DOX* genes were obtained from transcriptome dataset in various tissues of *S. tuberosum* Group Phureja DM1-3 in the Spud DB potato genomics resource (http://solanaceae.plantbiology.msu.edu/index.shtml).

Gene ID	Transcript ID	RH Stamen	RH Tuber Pith	RH Tuber Peel	**RH tuber sprout**	RH Tuber Cortex	RH Flower	RH Leaf	RH Petiole	RH Shoot Apex	RH Stem	RH Stolon	RH Young tuber	RH Mature Tuber	RH Root
Sotub07g016570 (St16DOX)	PGSC0003DMT400030676	1	1	80	**843**	4	339	1954	10	1298	301	2214	2279	109	14
**Sotub01g007130 (DPS)**	PGSC0003DMT400008936	0	0	17	**88**	1	3	22	0	99	3	39	217	7	3
Sotub02g031570	PGSC0003DMT400064315	79	39	58	**51**	36	90	30	42	22	19	23	23	20	67
Sotub01g007110 (St7110)	PGSC0003DMT400008930	0	0	7	**49**	0	0	16	0	52	4	27	107	4	2
Sotub01g007100 (St7100)	PGSC0003DMT400082027	0	0	7	**49**	0	1	7	0	59	1	17	102	3	2
Sotub01g007070 (St7070)	PGSC0003DMT400081912	0	0	6	**48**	0	0	9	0	51	2	16	78	5	1
Sotub09g026180	PGSC0003DMT400044419	1	8	19	**39**	5	15	21	33	31	30	43	57	6	22
Sotub06g018240	PGSC0003DMT400079111	9	63	70	**38**	65	17	17	45	74	32	27	43	79	52
Sotub04g026810	PGSC0003DMT400045704	8	12	14	**36**	16	33	12	19	145	38	73	124	43	17
Sotub03g017050	PGSC0003DMT400001512	1	0	16	**36**	1	4	16	100	3	335	27	10	8	30
Sotub03g029700	PGSC0003DMT400001594	1	5	4	**36**	5	3	7	1	11	4	4	14	8	21
Sotub02g024300	PGSC0003DMT400043087	5	1	0	**35**	2	61	36	51	20	15	27	49	5	90
Sotub03g029710	PGSC0003DMT400001611	0	3	1	**33**	3	1	3	1	7	2	1	11	5	22
Sotub02g031540	PGSC0003DMT400064257	10	23	31	**32**	21	18	13	22	23	13	24	22	22	38
Sotub02g011530	PGSC0003DMT400033881	9	28	27	**31**	23	11	12	16	26	17	26	20	13	32
Sotub01g042800	PGSC0003DMT400083733	0	59	56	**31**	48	8	15	26	7	22	3	3	25	71
Sotub04g007350	PGSC0003DMT400075876	17	18	16	**31**	21	21	17	24	39	28	26	40	24	17
Sotub03g029750 or Sotub03g029760	PGSC0003DMT400084684	0	5	3	**31**	6	5	8	1	12	5	3	16	7	25
Sotub04g007790	PGSC0003DMT400075915	43	10	17	**31**	14	49	56	41	7	16	14	5	11	41
Sotub03g029750	PGSC0003DMT400001604	1	5	3	**30**	5	5	10	1	14	4	3	14	7	23
Sotub02g027300	PGSC0003DMT400009156	6	23	25	**30**	25	8	10	17	14	14	12	15	15	37
Sotub11g028800	PGSC0003DMT400070461	0	1	2	**29**	0	7	29	15	6	38	21	6	0	1
Sotub05g009120	PGSC0003DMT400078389	22	17	14	**29**	18	26	21	32	23	24	24	24	17	29
Sotub02g017740	PGSC0003DMT400007992	1	0	22	**28**	1	12	41	7	5	1	18	6	4	33
Sotub01g022480	PGSC0003DMT400081749	15	33	26	**28**	27	18	11	26	32	33	15	44	20	29
Sotub03g019160	PGSC0003DMT400056112	28	10	14	**27**	13	22	16	25	21	20	53	21	12	34
Sotub02g031170	PGSC0003DMT400064198	1	23	17	**27**	24	3	5	4	37	13	9	17	29	9
Sotub09g021590	PGSC0003DMT400009727	10	5	8	**25**	10	21	19	19	8	16	9	17	10	4
Sotub11g028790	PGSC0003DMT400070465	1	4	4	**23**	2	18	40	33	10	51	27	15	6	4
Sotub02g008310	PGSC0003DMT400055332	29	0	5	**22**	1	248	472	163	2	18	142	30	0	1

Supplementary Table 2| ^{13}C and ^{1}H assignments for the aglycon of the DPS enzymatic reaction product. TMS (0.00 ppm) was used as an internal standard. Values in parentheses denote multiplicity and coupling constants (in Hz) for each signal. [a] Data after H-D exchange are presented. [b] Severely broadened signal due to C-D coupling. [c] Before H-D exchange, the corresponding signals were observed as follows: 2.84 (br ddd, J = 21.3, 8.9, 6.8 Hz); and 3.12 (1H, br ddd, J = 21.3, 4.1, 4.1).

solvent		CD_3SOCD_3		CD_3OD[a]	
		δ_C	δ_H	δ_C	δ_H
C/H		201 MHz	800 MHz	151 MHz	600 MHz
1	α	36.29	0.94 (m)	38.12	1.01 (m)
	β		1.64 (m)		1.73 (m)
2	α	28.90	1.74 (m)	30.40	1.88 (m)
	β		1.39 (m)		1.53 (m)
3		76.26	3.53 (dddd, 11.0, 11.0, 4.7, 4.7)	79.27	3.68 (dddd, 11.2, 11.2, 4.8, 4.8)
4	α	33.86	1.62 (m)	35.30	1.71 (m)
	β		1.17 (m)		1.32 (m)
5		43.81	1.07 (m)	45.92	1.13 (m)
6	α	28.07	1.28 (m)	29.72	1.34 (m)
	β		1.21 (m)		1.32 (m)
7	α	31.57	0.96 (m)	33.32	1.04 (m)
	β		1.60 (m)		1.73 (m)
8		33.96	1.43 (m)	35.82	1.60 (m)
9		53.14	0.74 (m)	55.33	0.79 (ddd, 11.4, 10.8, 3.9)
10		35.19	–	36.88	–
11	α	20.01	1.51 (m)	21.81	1.62 (m)
	β		1.23 (m)		1.35 (m)
12	α	37.83	1.24 (m)	40.00	1.37 (m)
	β		1.74 (m)		1.85 (m)
13		42.83	–	45.05	–
14		52.56	1.44 (m)	54.58	1.46 (m)
15	α	36.95	2.00 (dd, 13.1, 4.9)	38.38	2.21 (dd, 12.9, 5.5)
	β		1.83 (m)		1.84 (dd, 13.9, 12.9)
16		110.95	–	117.85	–
17		63.90	1.81 (br s)	58.74	2.16 (d, 2.1)
18		13.16	0.54 (s)	14.93	0.70 (s)
19		11.92	0.77 (s)	12.67	0.86 (s)
20		41.43	3.24 (m)	43.70	3.37 (m)
21		18.61	1.33 (d, 7.6)	17.76	1.45 (d, 7.6)
22		192.21	–	196.80	–
23	a	24.71	2.86 (br ddd, 21.4, 7.5, 7.5)	26.38[b]	–[c]
	b		3.08 (br ddd, 21.4, 5.4, 5.4)		–[c]
24	a	23.61	1.45 (m)	25.35	1.55 (dd, 13.8, 11.1)
	b		1.83 (m)		1.97 (ddd, 13.8, 3.4, 1.2)
25		25.55	2.09 (m)	27.94	2.10 (m)
26	a	47.80	3.28 (dd, 15.0, 8.7)	50.68	3.18 (m)
	b		3.78 (m)		3.81 (m)
27		16.98	0.99 (d, 6.7)	18.03	1.12 (d, 6.7)

Supplementary Table 3| ^{13}C and ^{1}H assignments for the sugar moiety of the DPS enzymatic reaction product. TMS (0.00 ppm) was used as an internal standard. Values in parentheses denote multiplicity and coupling constants (in Hz) for each signal. [a] Data after H-D exchange are presented.

solvent		CD_3SOCD_3		CD_3OD^a	
		δ_C	δ_H	δ_C	δ_H
C/H		201 MHz	800 MHz	151 MHz	600 MHz
Gal	1	100.86	4.21 (d, 7.7)	102.69	4.37 (d, 7.7)
	2	71.39	3.20 (dd, 9.4, 7.7)	73.20	3.61 (dd, 9.8, 7.7)
	3	73.46	3.33 (m)	75.65	3.50 (dd, 9.8, 3.3)
	4	78.91	3.78 (m)	80.21	4.02 (br d, 3.3)
	5	73.74	3.34 (dd, 6.6, 5.7)	75.35	3.50 (m)
	6	59.39	3.39 (dd, 10.6, 5.7)	61.10	3.61 (m)
			3.73 (dd, 10.6, 6.6)		3.91 (m)
Internal-Glc	1	103.23	4.41 (d, 7.7)	104.76	4.58 (d, 7.8)
	2	79.27	3.57 (dd, 8.9, 7.7)	81.09	3.75 (dd, 8.9, 7.8)
	3	84.89	3.60 (dd, 8.9, 8.9)	87.93	3.70 (dd, 8.9, 8.6)
	4	68.71	3.12 (dd, 8.9, 8.9)	70.49	3.27 (m)
	5	75.81	3.23 (m)	77.53	3.33 (m)
	6	61.23	3.37 (dd, 10.7, 7.6)	63.16	3.57 (dd, 11.6, 7.5)
			3.71 (dd, 10.7, 10.7)		3.90 (m)
External-Glc	1	102.32	4.71 (d, 8.1)	104.32	4.91 (d, 8.1)
	2	74.20	2.97 (dd, 8.1, 8.1)	75.91	3.18 (dd, 8.9, 8.1)
	3	75.98	3.15 (m)	78.01	3.35 (m)
	4	69.58	3.14 (m)	71.59	3.34 (m)
	5	76.88	3.14 (m)	78.52	3.32 (m)
	6	60.80	3.56 (m)	62.74	3.81 (dd, 12.3, 5.5)
			3.72 (m)		3.88 (br d, 12.3)
Xyl	1	103.23	4.50 (d, 7.8)	104.99	4.60 (d, 7.7)
	2	73.42	3.04 (dd, 8.9, 7.8)	75.29	3.24 (dd, 9.3, 7.7)
	3	76.46	3.12 (dd, 8.9, 8.9)	78.35	3.30 (m)
	4	69.32	3.32 (m)	71.01	3.51 (m)
	5	65.79	3.10 (dd, 11.0, 8.9)	67.23	3.26 (m)
			3.77 (dd, 11.0, 5.2)		3.91 (m)

Supplementary Table 4| Kinetic parameters of DPS and St7070 with α-solamarine and tomatidenol. Kinetic parameters were determined by non-liner regression with ANEMONA.

Enzyme	Substrate	K_m (μM)	k_{cat} (s^{-1})	k_{cat}/K_m (μM^{-1} s^{-1})
DPS	α-solamarine	4.6 ± 0.19	0.67 ± 0.08	0.15
St7070	α-solamarine	1.7 ± 0.24	0.064 ± 0.011	0.038
DPS	(*22S*,*25S*)-spirosol -5-en-3β-ol	170 ± 0.34	0.023 ± 0.013	0.00013
St7070	(*22S*,*25S*)-spirosol -5-en-3β-ol	19.6 ± 0.056	0.17 ± 4.5E-06	0.0085

Supplementary Table 5| RPKM values of Sl6585, Sl16DOX, and Sl23DOX in tomato. The RPKM values of Sl6585, Sl16DOX, and Sl23DOX from transcriptome dataset in various tissues of *S. lycopersicum* cv. Heinz in Tomato Functional Genomics Database (http://ted.bti.cornell.edu/) are shown.

Gene ID	Unopened flower bud	Fully opened flower	Leaf	Root	1 cm fruit	2 cm fruit	3 cm fruit	Mature green fruit	Breaker fruit	Breaker fruit + 10 days
Solyc01g006585 (Sl6585)	0	0	0	0	0	0	0	0	2	0.3
Solyc07g043420 (Sl16DOX)	2474	983	1280	441	5013	1405	329	49	8	0
Solyc02g062460 (Sl23DOX/SlGAME31)	74	27	1	10	14	15	10	117	186	568

Supplementary Table 6| FPKM values of SMEL_001g151230 and SMEL_007g282440 in eggplant. The FPKM values of SMEL_001g151230 (the DPS ortholog) and SMEL_007g282440 (the 16DOX ortholog) were obtained from transcriptome dataset in various tissues of *S. melongena*[28].

Gene_ID	Root	Leaf	Flower	Fruit		
				Stage 1	Stage 2	Stage 3
SMEL_001g151230	32.0899	0	0.335754	2.18488	0.42712	0
SMEL_007g282440 (16DOX)	320.815	138.129	221.691	616.872	145.852	88.8157

Supplementary Table 7| PCR primers used in this work.

No.	primer name	sequence (5` to 3`)
1	DPS qPCR Fw	GAGAGTGGATAGAGCTCAATAATACA
2	DPS qPCR Rv	CTCCTGTTGTTACTACTCTGTGTTC
3	St7070 qPCR Fw	GATTACCTTCCCACACAGATAGTTC
4	St7070 qPCR Rv	CTATCATTTGTCCATGCCATAAG
5	StEf1α qPCR Fw	ATTGGAAACGGATATGCTCCA
6	StEf1α qPCR Rv	TCCTTACCTGAACGCCTGTCA
7	DPS cloning Fw	CATATGGCATCTACCAAAGTTAAGATTCCC
8	DPS cloning RV	GTCGACTTAAAGACCACAATAATTCTTGAGATTAATTCA
9	St7070 cloning Fw	CATATGGCATCTACCAAAGTTACGATTCCC
10	St7070 cloning RV	GTCGACTTAAAGACCACAATAATTCTTGACACCC
11	AtPDS third intron Fw	TCTAGATTTGGATCCAAAGGTACTTTGATTGGTCTC
12	AtPDS third intron Rv	GGATCCCAACACTATTTGGGAGGACCAA
13	DPS fragment Fw	GTCGACTCTAGACCAAACACTCCACAATGGGA
14	DPS fragment Rv	CATATGCTCGAGCAACACTATTTGGGAGGACCAA
15	NPT2 Fw	TAAAGCACGAGGAAGCGGT
16	NPT2 Rv	GCACAACAGACAATCGGCT
