# Supplementary material for: The biosynthetic pathway of potato solanidanes diverged from that of spirosolanes due to evolution of a dioxygenase
Source: Nat Commun. 2021 Feb 26;12:1300. doi: 10.1038/s41467-021-21546-0 (PMC7910490; doi:10.1038/s41467-021-21546-0)
Supplement: Supplementary file 2 — Descriptions of Additional Supplementary Files [file 41467_2021_21546_MOESM2_ESM.pdf]

Descriptions of Additional Supplementary Files

Supplementary Data 1

Description: NMR Data

Supplementary Data 2

Description: List of DOX genes
