# Supplementary material for: The biosynthetic pathway of potato solanidanes diverged from that of spirosolanes due to evolution of a dioxygenase
Source: Nat Commun. 2021 Feb 26;12:1300. doi: 10.1038/s41467-021-21546-0 (PMC7910490; doi:10.1038/s41467-021-21546-0)

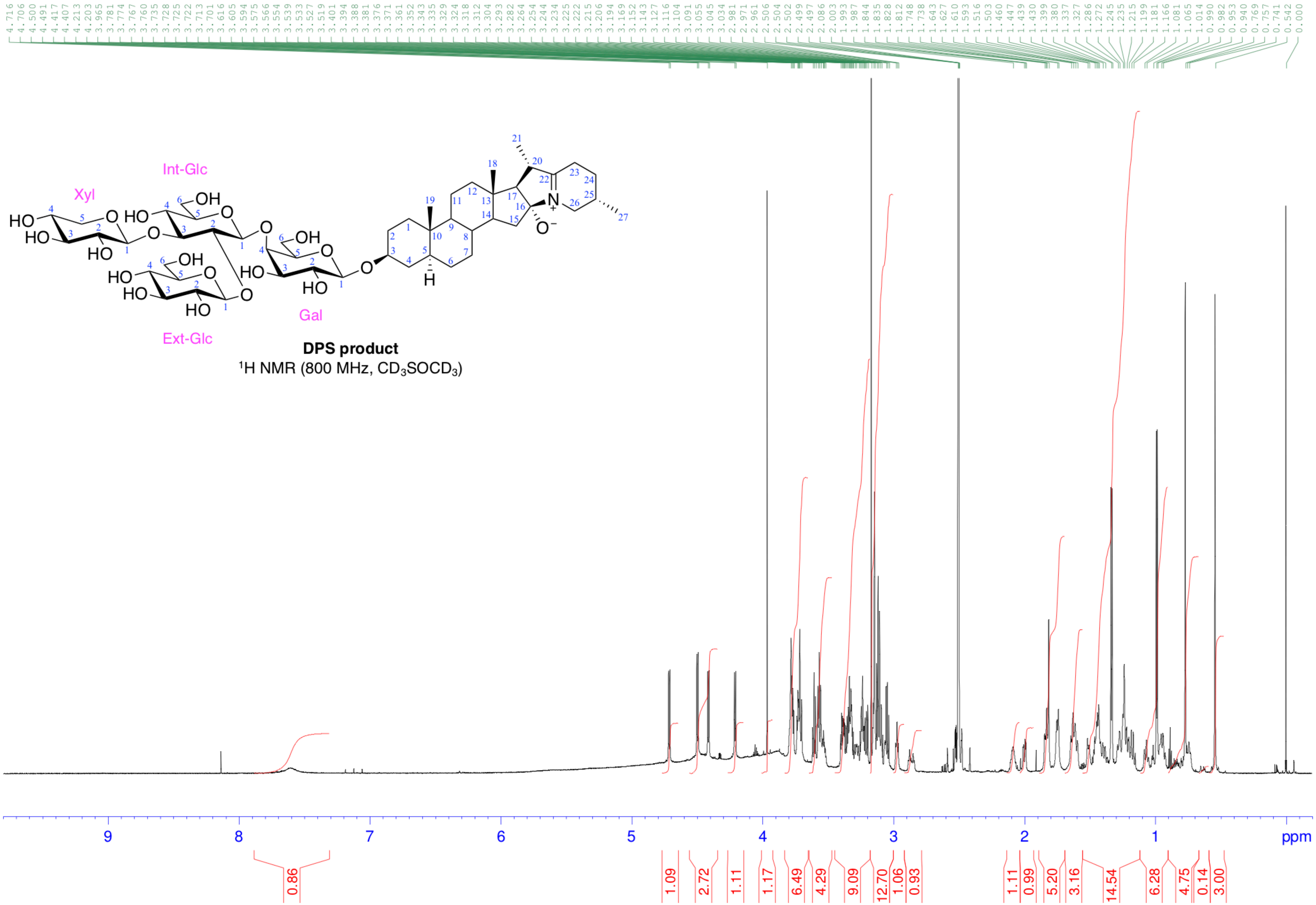
4.716 4.706 4.500 4.491 4.417 4.407 4.213 4.203 3.965 3.781 3.774 3.767 3.760 3.735 3.728 3.725 3.722 3.713 3.701 3.616 3.605 3.594 3.575 3.566 3.554 3.539 3.533 3.527 3.519 3.401 3.394 3.388 3.381 3.376 3.371 3.361 3.352 3.343 3.335 3.329 3.324 3.318 3.312 3.304 3.293 3.282 3.264 3.254 3.244 3.234 3.225 3.222 3.215 3.206 3.194 3.169 3.152 3.143 3.127 3.116 3.104 3.091 3.055 3.045 3.034 2.981 2.971 2.961 2.506 2.504 2.502 2.499 2.497 2.086 2.003 1.993 1.987 1.844 1.835 1.828 1.812 1.748 1.738 1.643 1.627 1.610 1.593 1.516 1.503 1.460 1.447 1.439 1.430 1.399 1.380 1.337 1.327 1.286 1.272 1.245 1.235 1.215 1.199 1.181 1.166 1.081 1.065 1.014 0.990 0.982 0.953 0.940 0.769 0.757 0.741 0.542 0.000
Xyl
Int-Glc
Ext-Glc
Gal
DPS product
^{1}H NMR (800 MHz, CD_3SOCD_3)
9
8
7
6
5
4
3
2
1
ppm
0.86 1.09 2.72 1.11 1.17 6.49 4.29 9.09 12.70 1.06 0.93 1.11 0.99 5.20 3.16 14.54 6.28 4.75 0.14 3.00

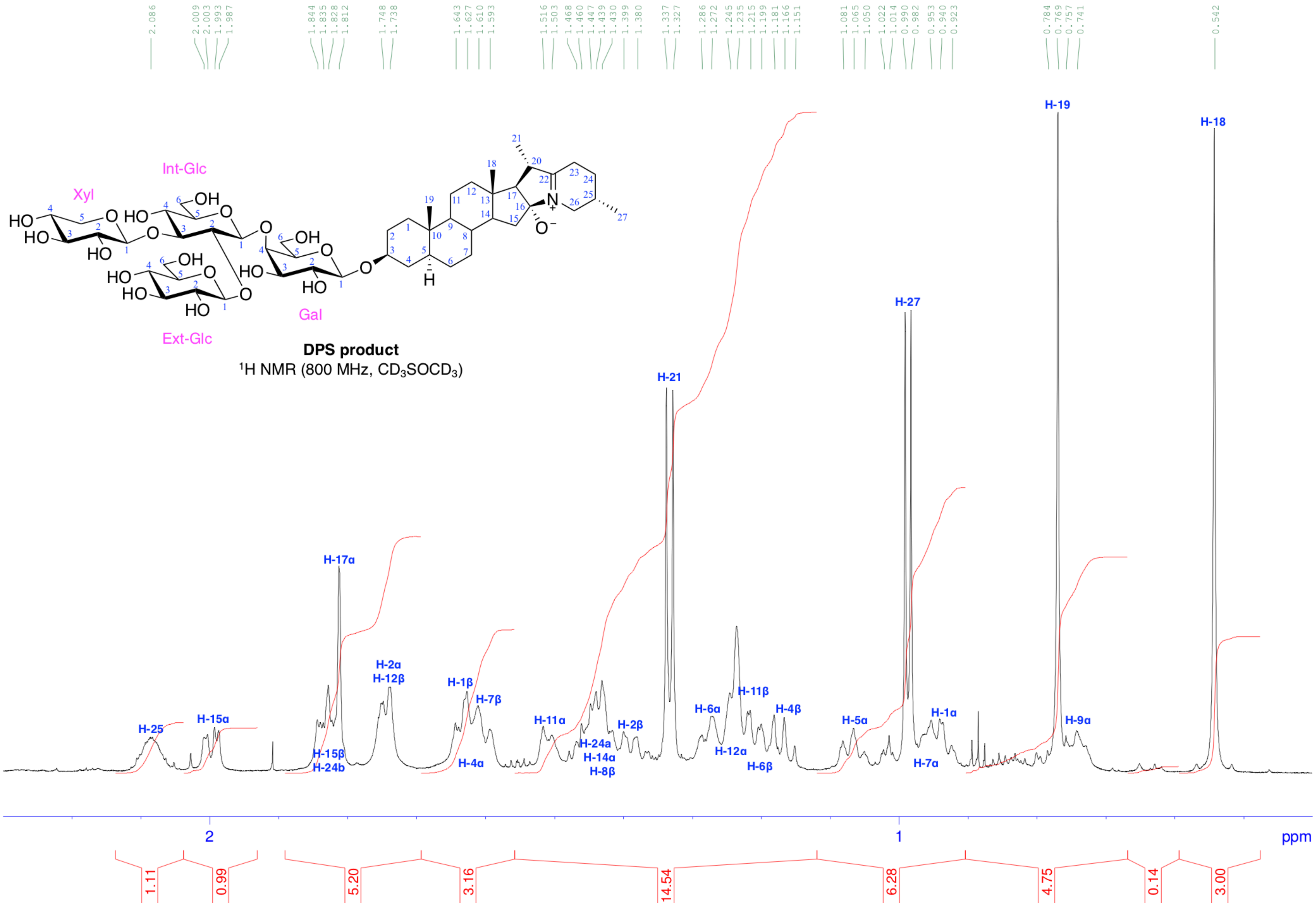

2.086
2.009
2.003
1.993
1.987
1.844
1.835
1.828
1.812
1.748
1.738
1.643
1.627
1.610
1.593
1.516
1.503
1.468
1.460
1.447
1.439
1.430
1.399
1.380
1.337
1.327
1.286
1.272
1.245
1.235
1.215
1.199
1.181
1.166
1.151
1.081
1.065
1.050
1.022
1.014
0.990
0.982
0.953
0.940
0.923
0.784
0.769
0.757
0.741
0.542
Xyl
Int-Glc
Ext-Glc
Gal
DPS product
¹H NMR (800 MHz, CD₃SOCD₃)
H-19
H-18
H-27
H-21
H-17α
H-2α
H-12β
H-1β
H-7β
H-25
H-15α
H-15β
H-24b
H-4α
H-11α
H-24a
H-14α
H-8β
H-2β
H-6α
H-11β
H-12α
H-6β
H-4β
H-5α
H-1α
H-7α
H-9α
2
1
ppm
1.11
0.99
5.20
3.16
14.54
6.28
4.75
0.14
3.00

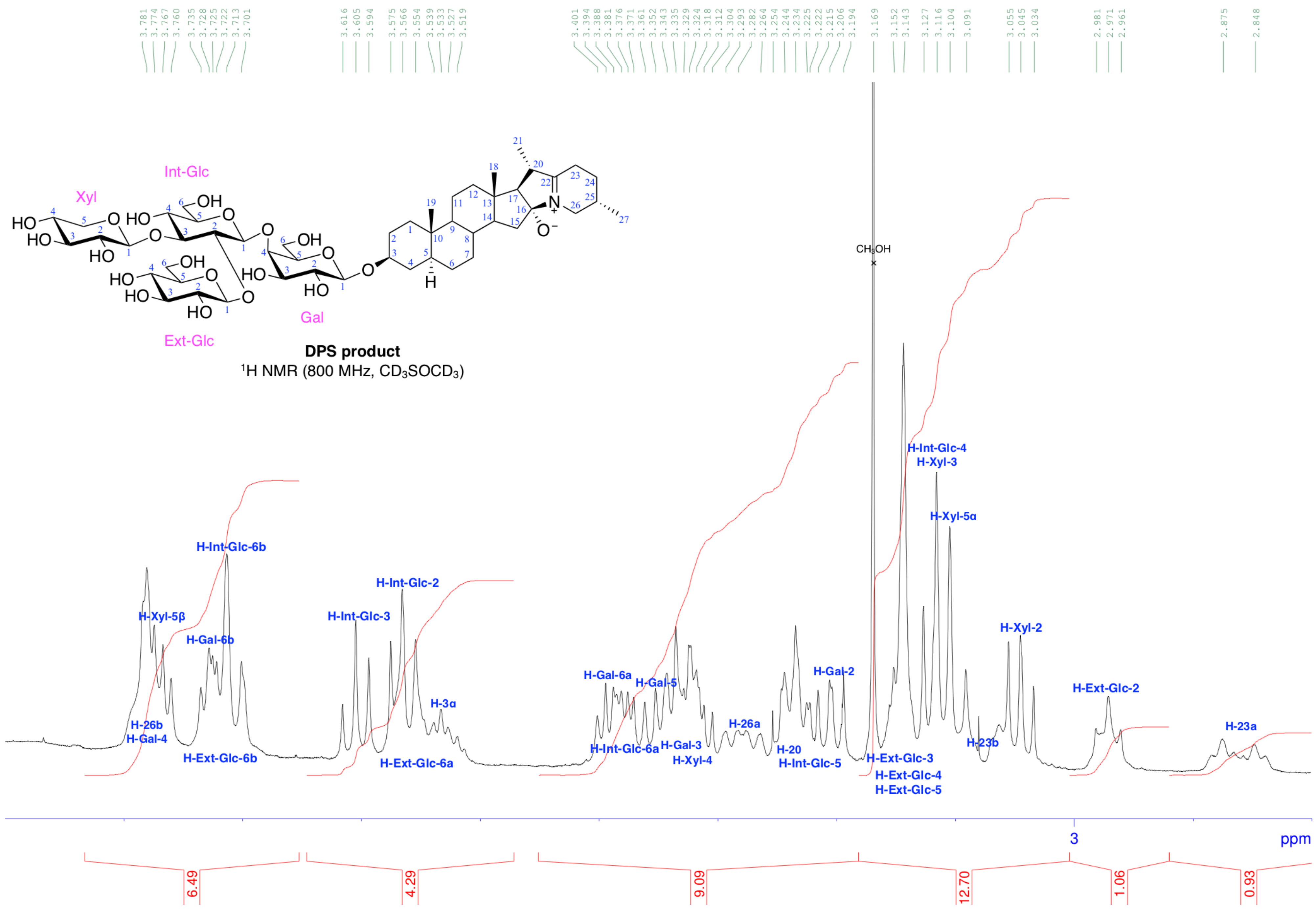

Xyl
Int-Glc
Ext-Glc
Gal
DPS product
^{1}H NMR (800 MHz, CD_3SOCD_3)
CH$_3$OH
H-26b
H-Gal-4
H-Xyl-5β
H-Gal-6b
H-Int-Glc-6b
H-Ext-Glc-6b
H-Int-Glc-3
H-Int-Glc-2
H-Ext-Glc-6a
H-3α
H-Gal-6a
H-Gal-5
H-Int-Glc-6a
H-Gal-3
H-Xyl-4
H-26a
H-20
H-Int-Glc-5
H-Gal-2
H-Ext-Glc-3
H-Ext-Glc-4
H-Ext-Glc-5
H-Int-Glc-4
H-Xyl-3
H-Xyl-5α
H-23b
H-Xyl-2
H-Ext-Glc-2
H-23a
3
ppm
6.49
4.29
9.09
12.70
1.06
0.93

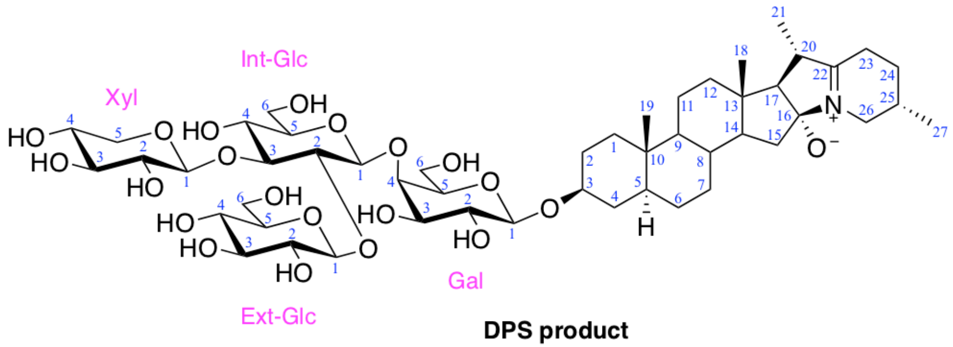

DPS product
^{13}C NMR (201 MHz, CD_3SOCD_3)

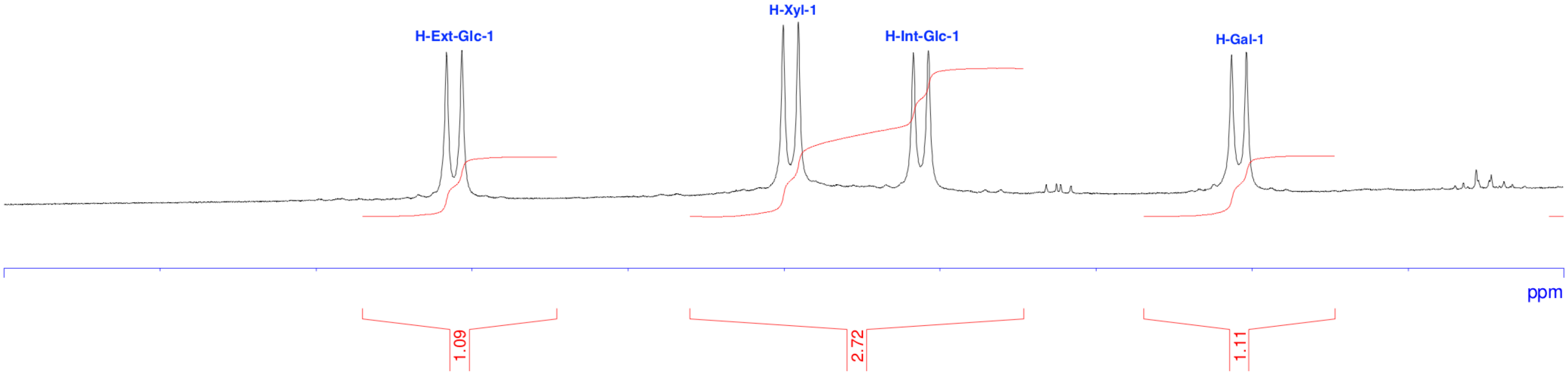

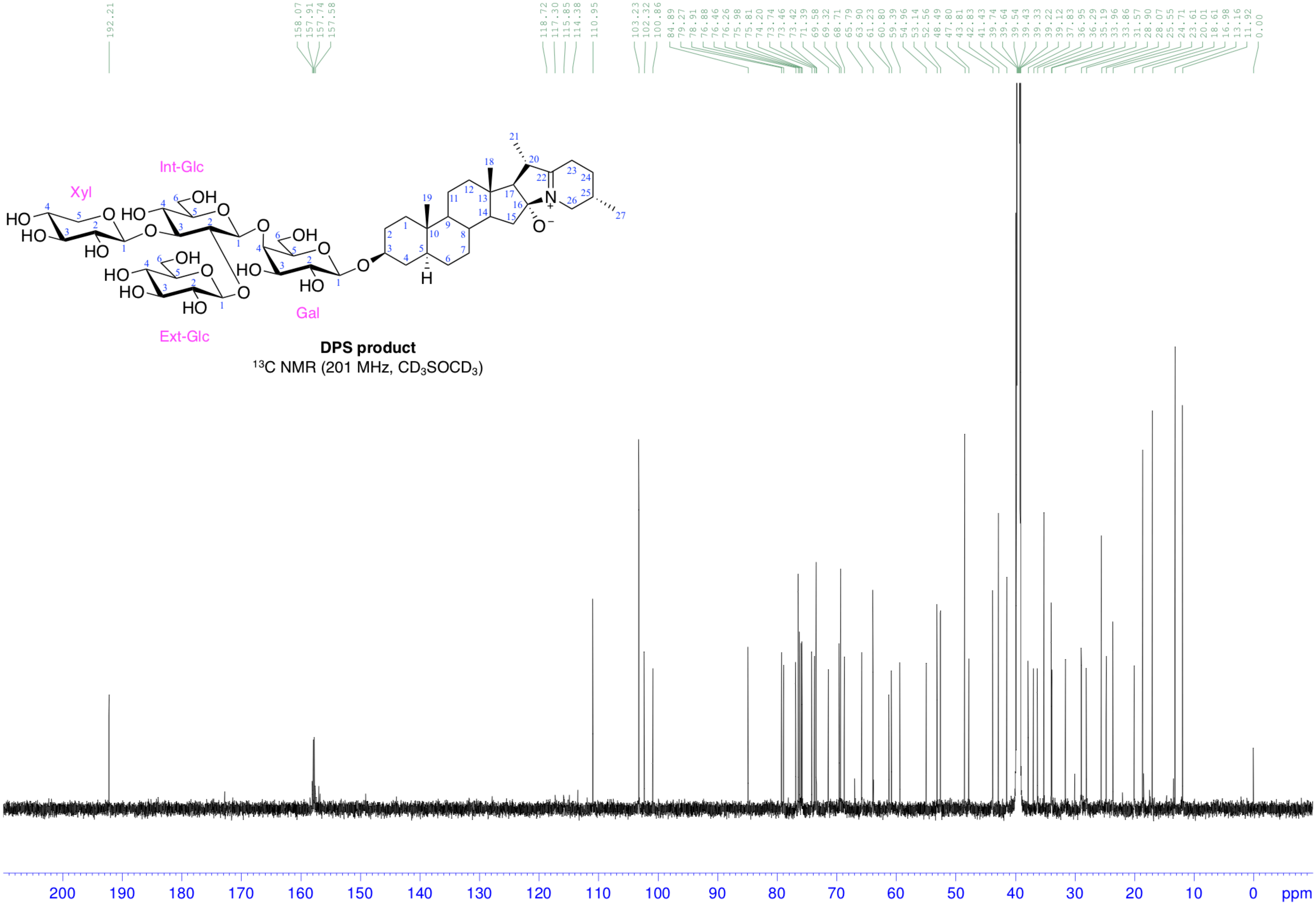

DPS product

^{13}C NMR (201 MHz, CD_3SOCD_3)

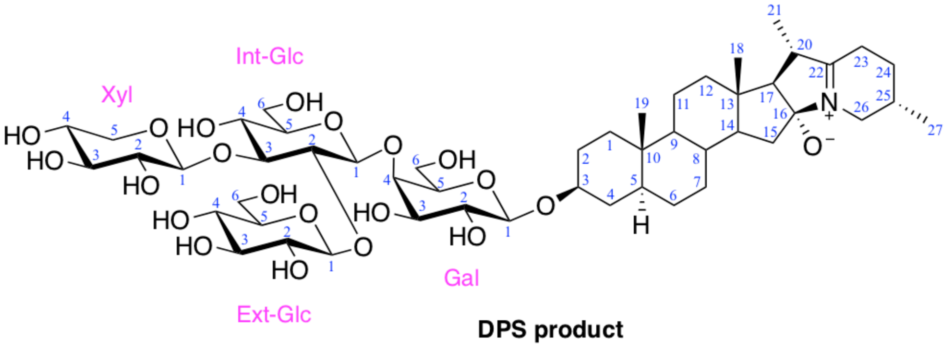

DPS product

^{13}C NMR (201 MHz, CD_3SOCD_3)

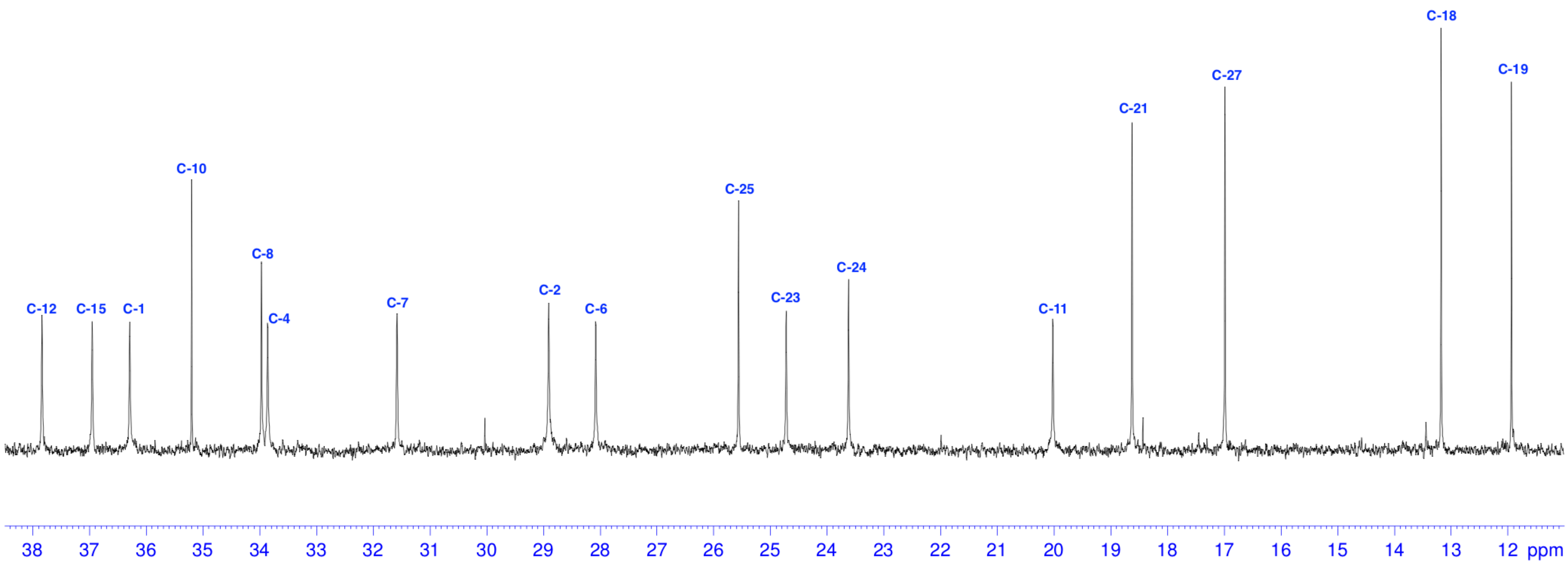

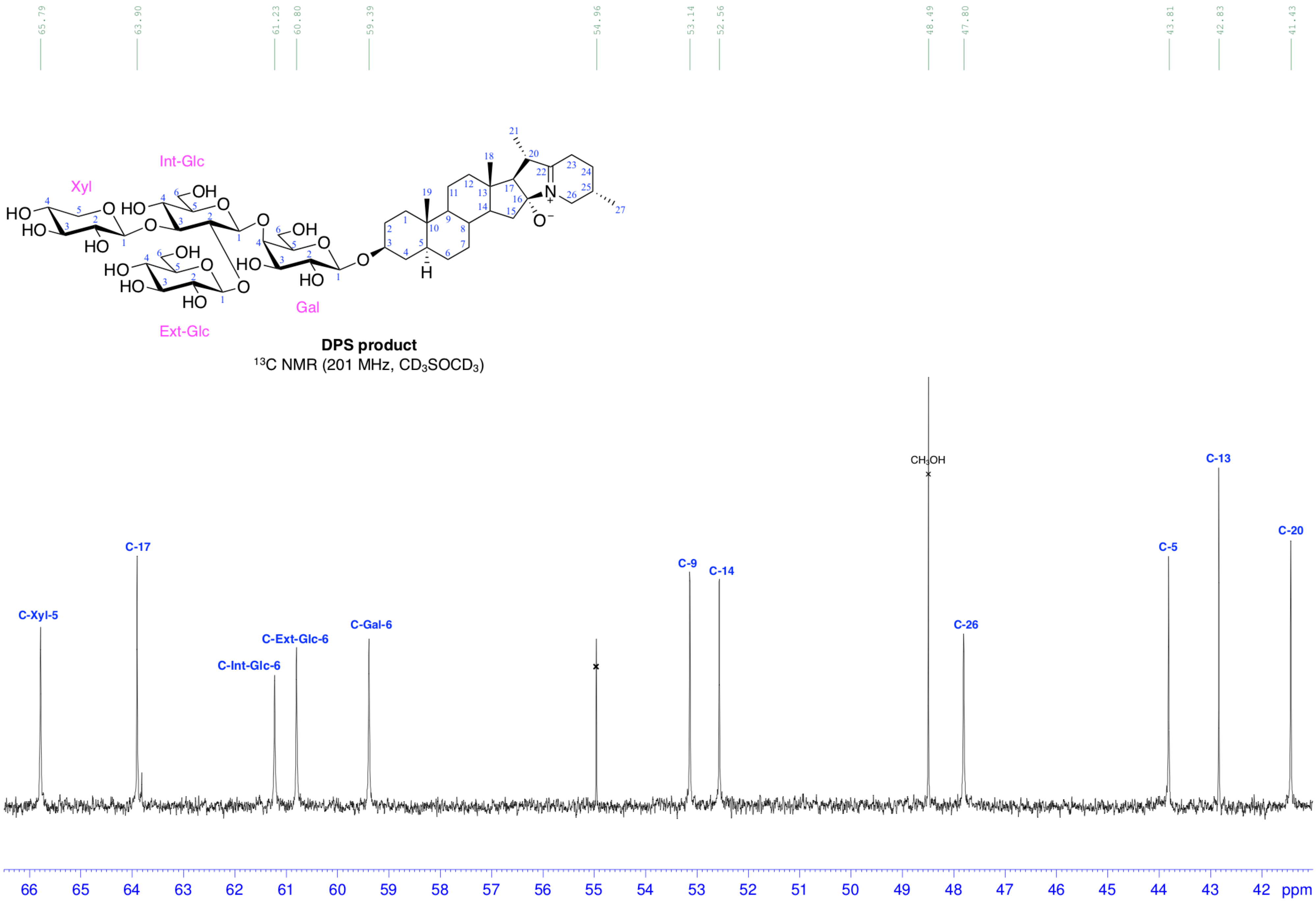

DPS product

^{13}C NMR (201 MHz, CD_3SOCD_3)

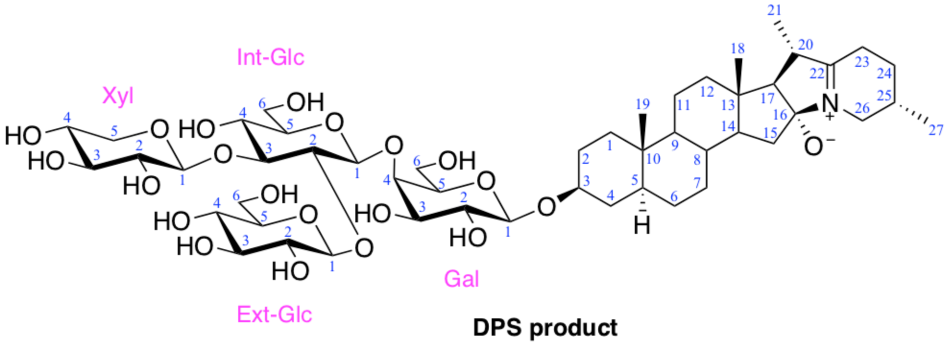

DPS product

^{13}C NMR (201 MHz, CD_3SOCD_3)

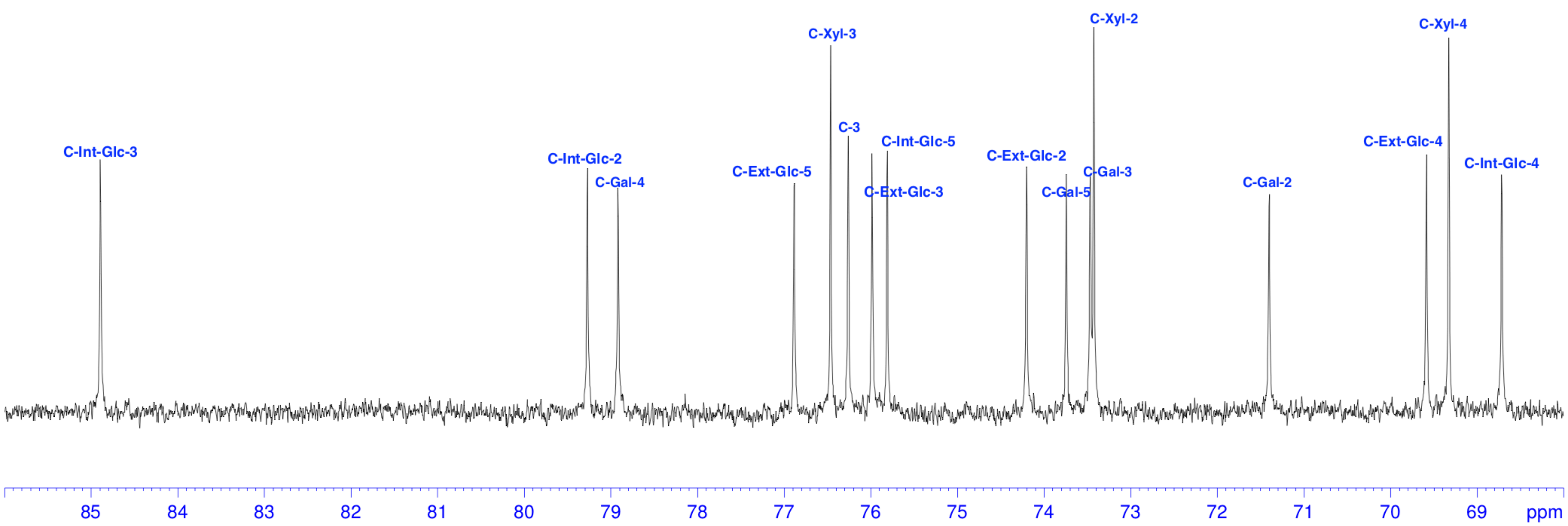

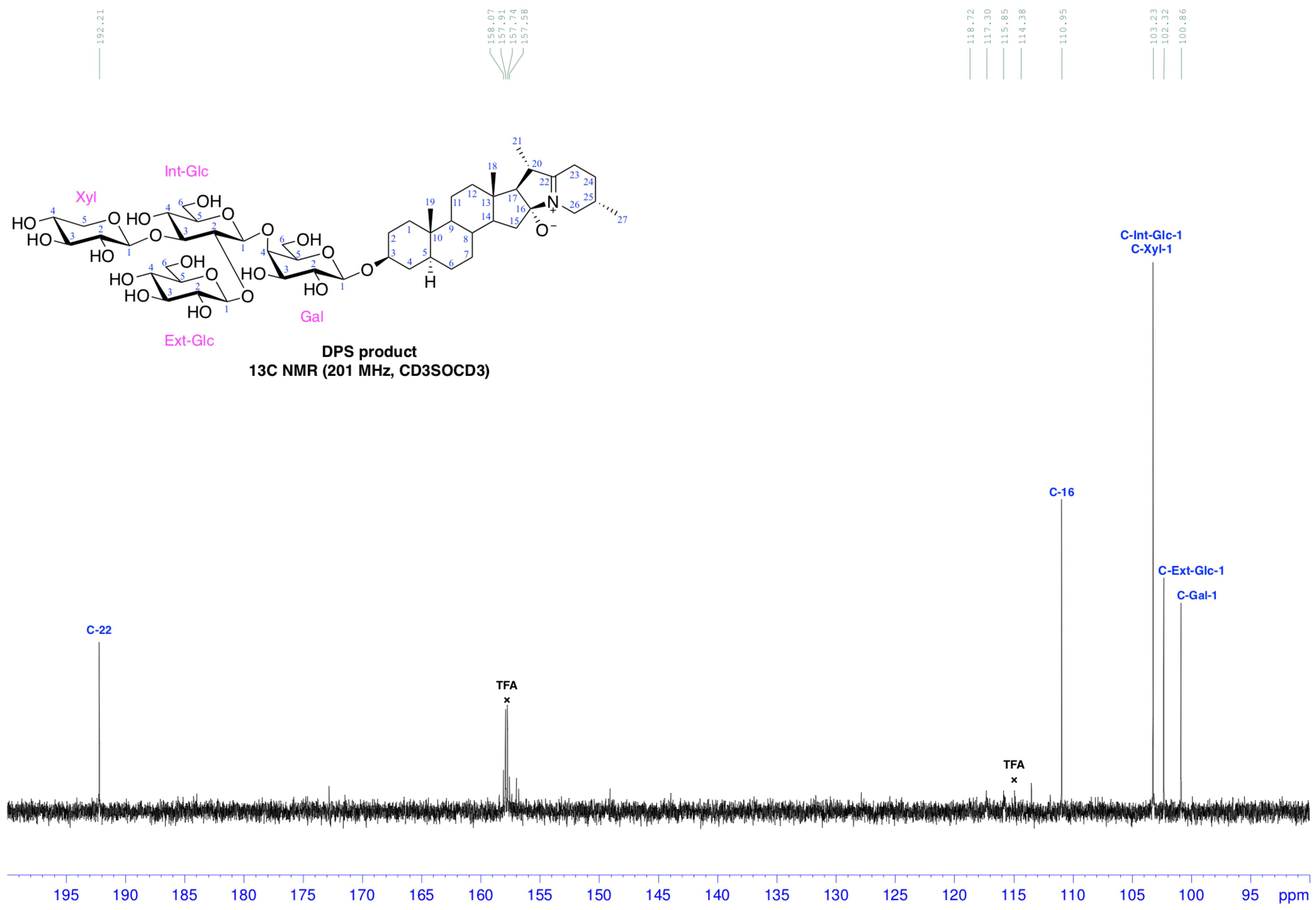
192.21
158.07
157.91
157.74
157.58
118.72
117.30
115.85
114.38
110.95
103.23
102.32
100.86
Xyl
Int-Glc
Ext-Glc
Gal
DPS product
13C NMR (201 MHz, CD3SOCD3)
C-Int-Glc-1
C-Xyl-1
C-16
C-Ext-Glc-1
C-Gal-1
C-22
TFA
TFA
195 190 185 180 175 170 165 160 155 150 145 140 135 130 125 120 115 110 105 100 95 ppm

DPS product

COSY (CD_3SOCD_3)

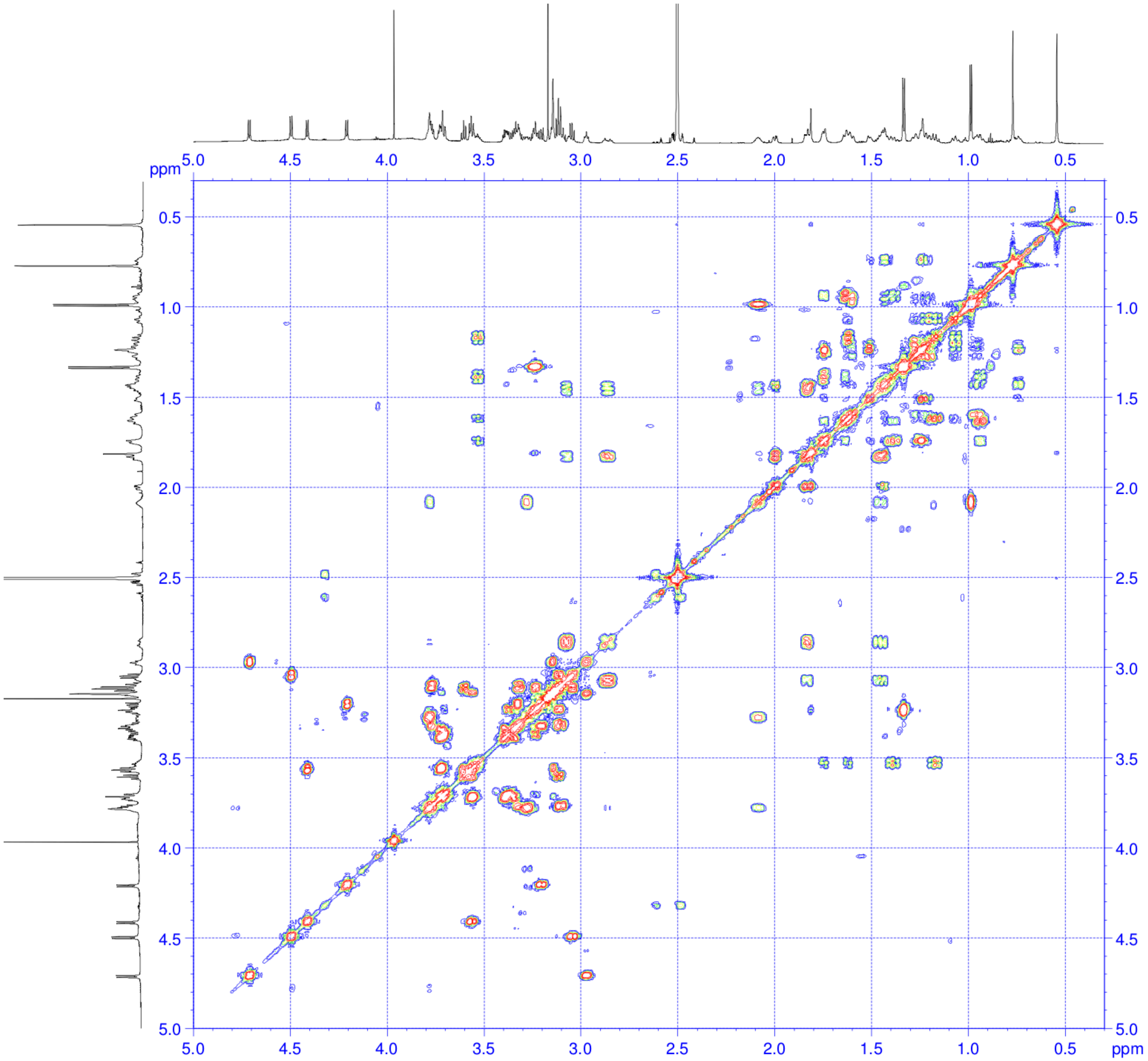

DPS product
NOESY (CD_3SOCD_3)

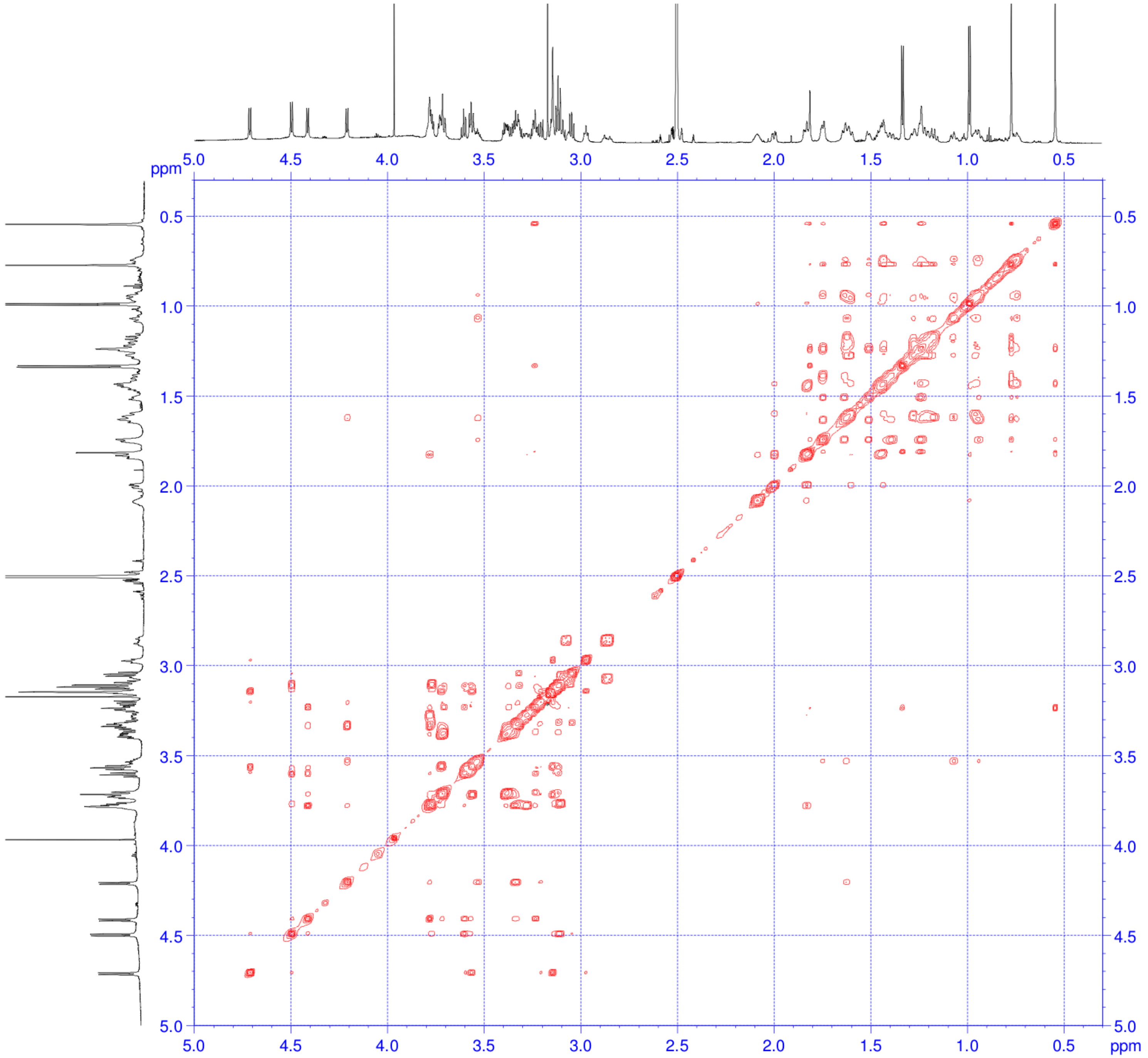

DPS product

HSQC (CD_3SOCD_3)

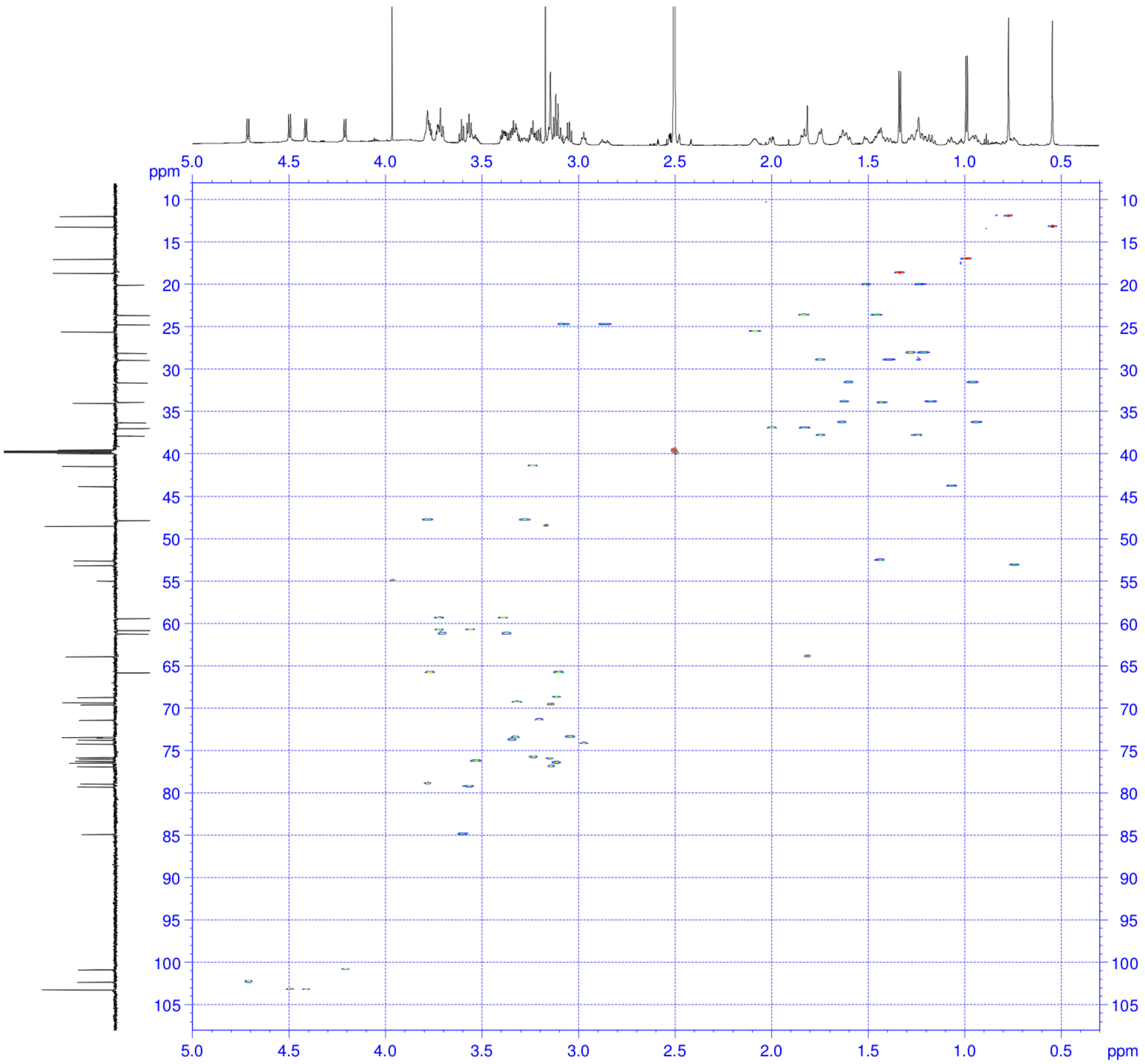

DPS product

HMBC (CD_3SOCD_3)

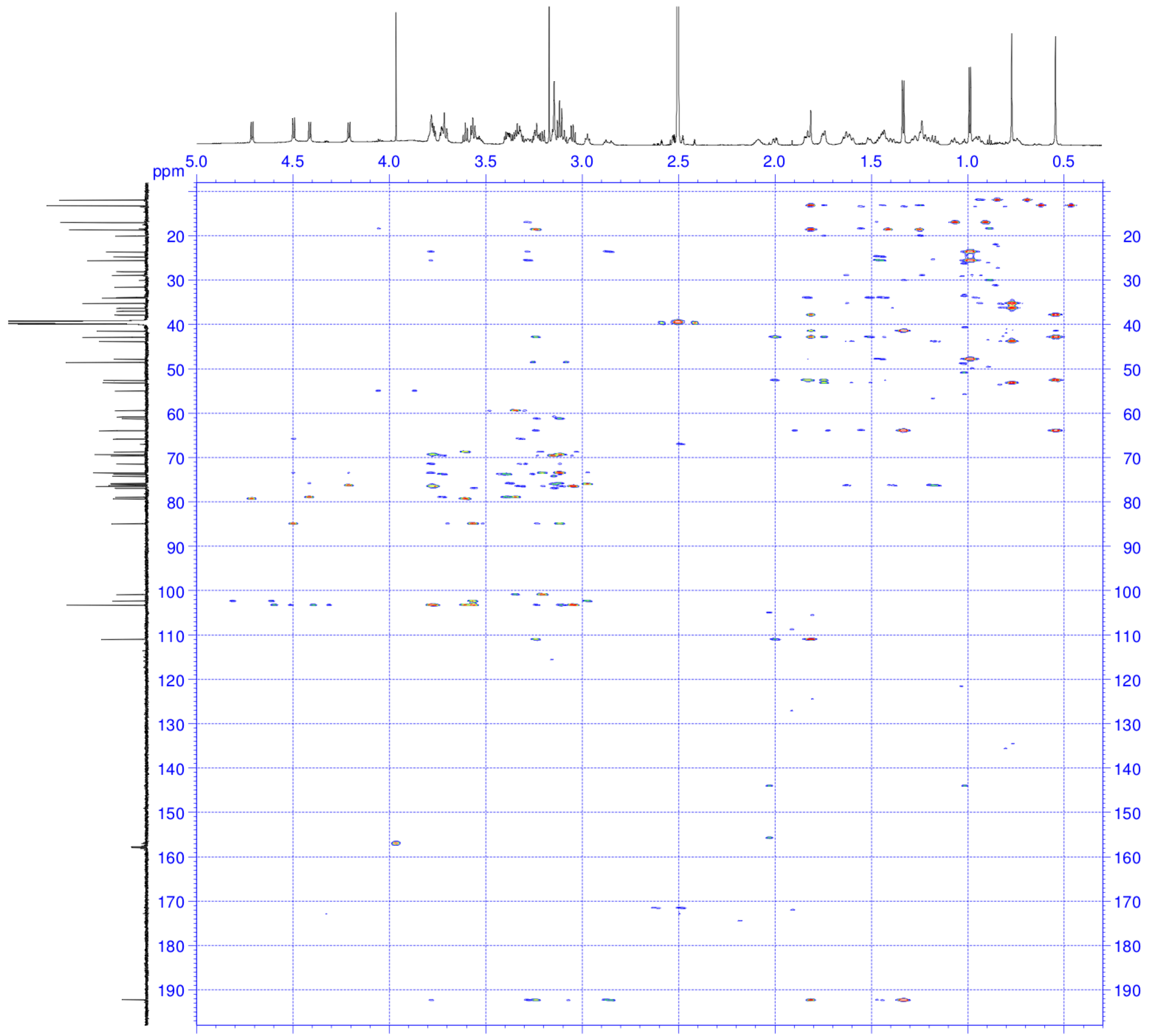

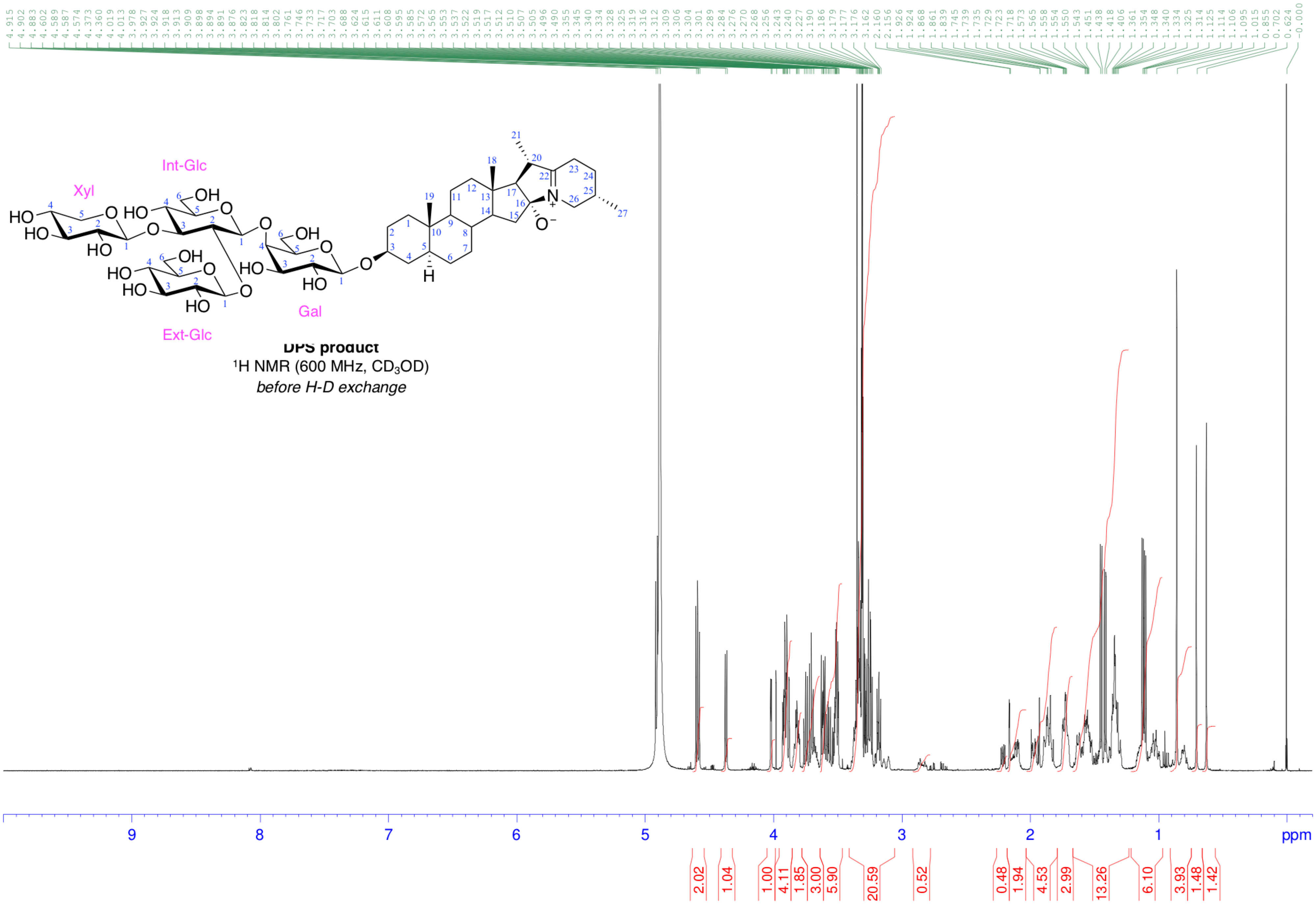
Xyl
Int-Glc
Ext-Glc
Gal
DPS product
^1H NMR (600 MHz, CD_3OD)
before H-D exchange
ppm
2.02
1.04
1.00
4.11
1.85
3.00
5.90
20.59
0.52
0.48
1.94
4.53
2.99
13.26
6.10
3.93
1.48
1.42

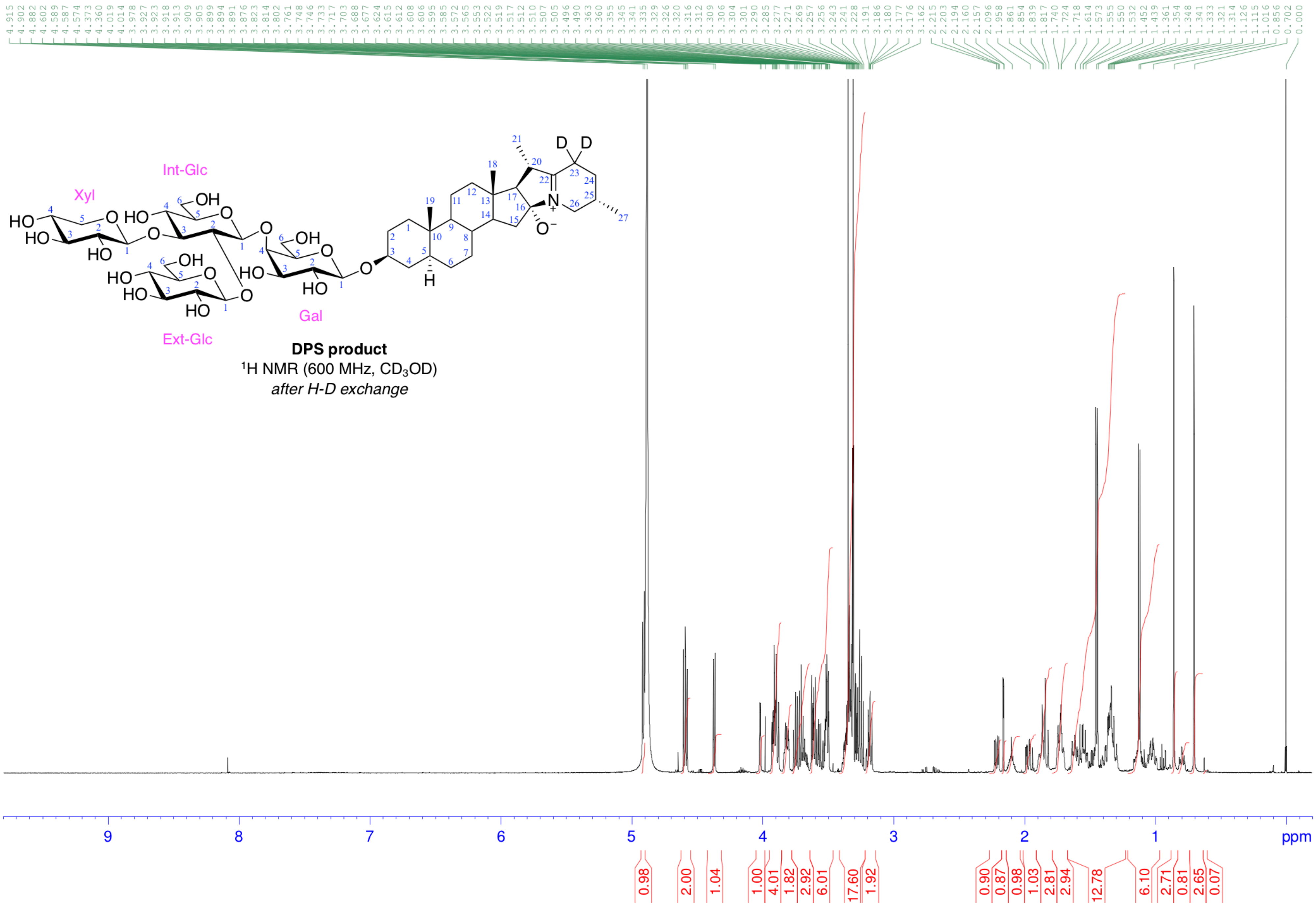

Xyl
Int-Glc
Ext-Glc
Gal
DPS product
¹H NMR (600 MHz, CD₃OD)
after H-D exchange
ppm

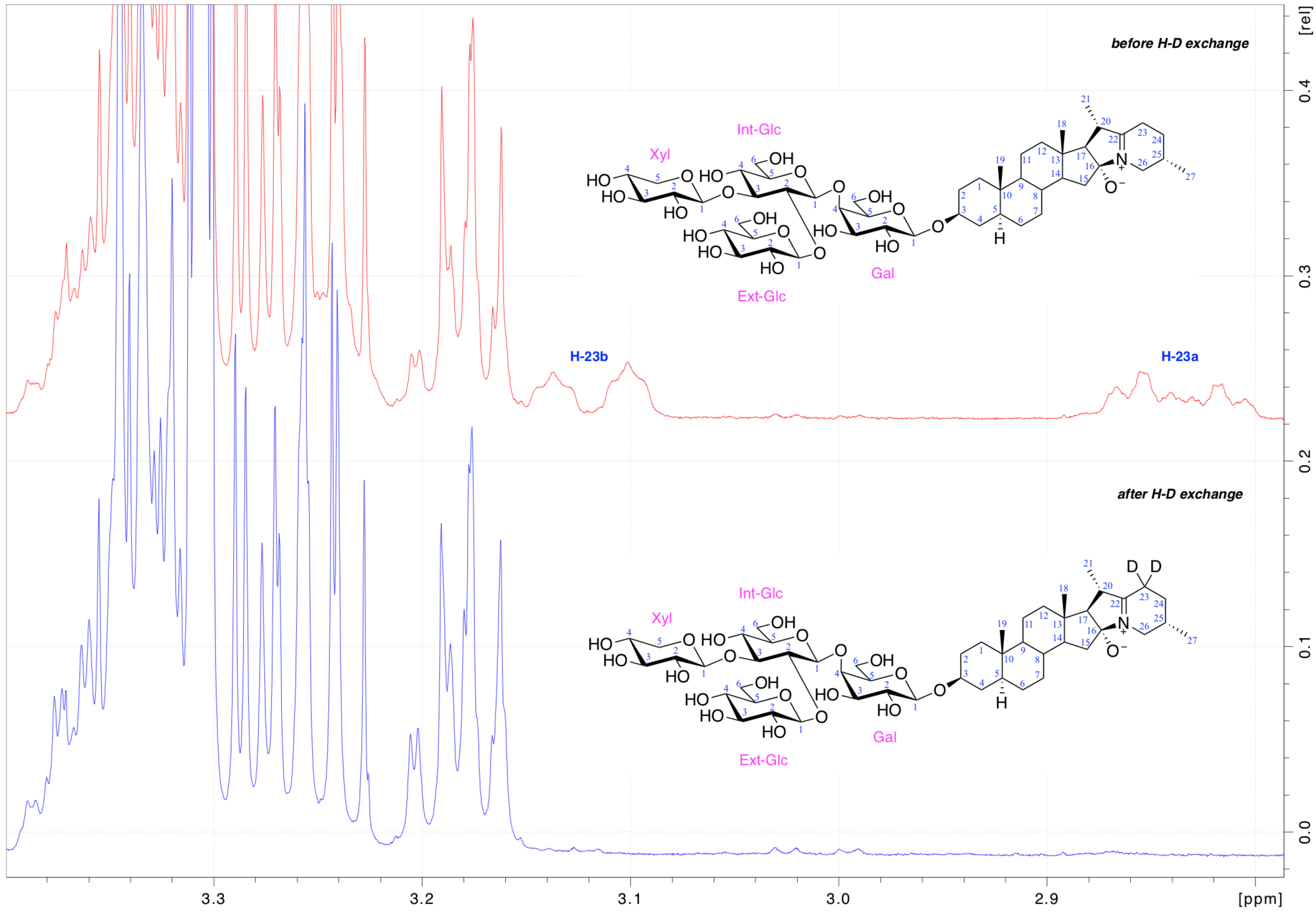

before H-D exchange
after H-D exchange
H-23b
H-23a
Xyl
Int-Glc
Ext-Glc
Gal
[rel]
[ppm]

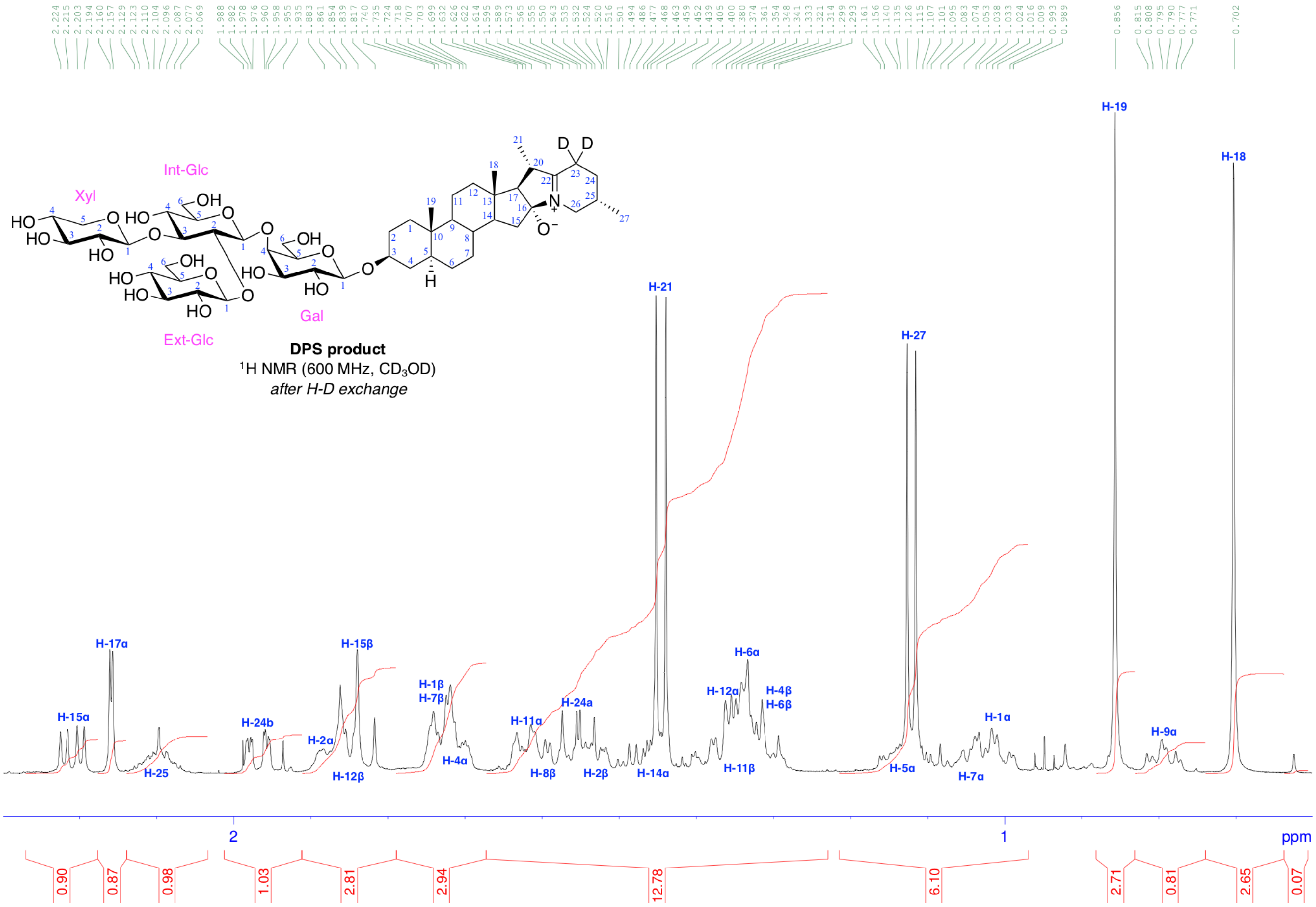

DPS product

^{1}H NMR (600 MHz, CD_3OD)

after H-D exchange

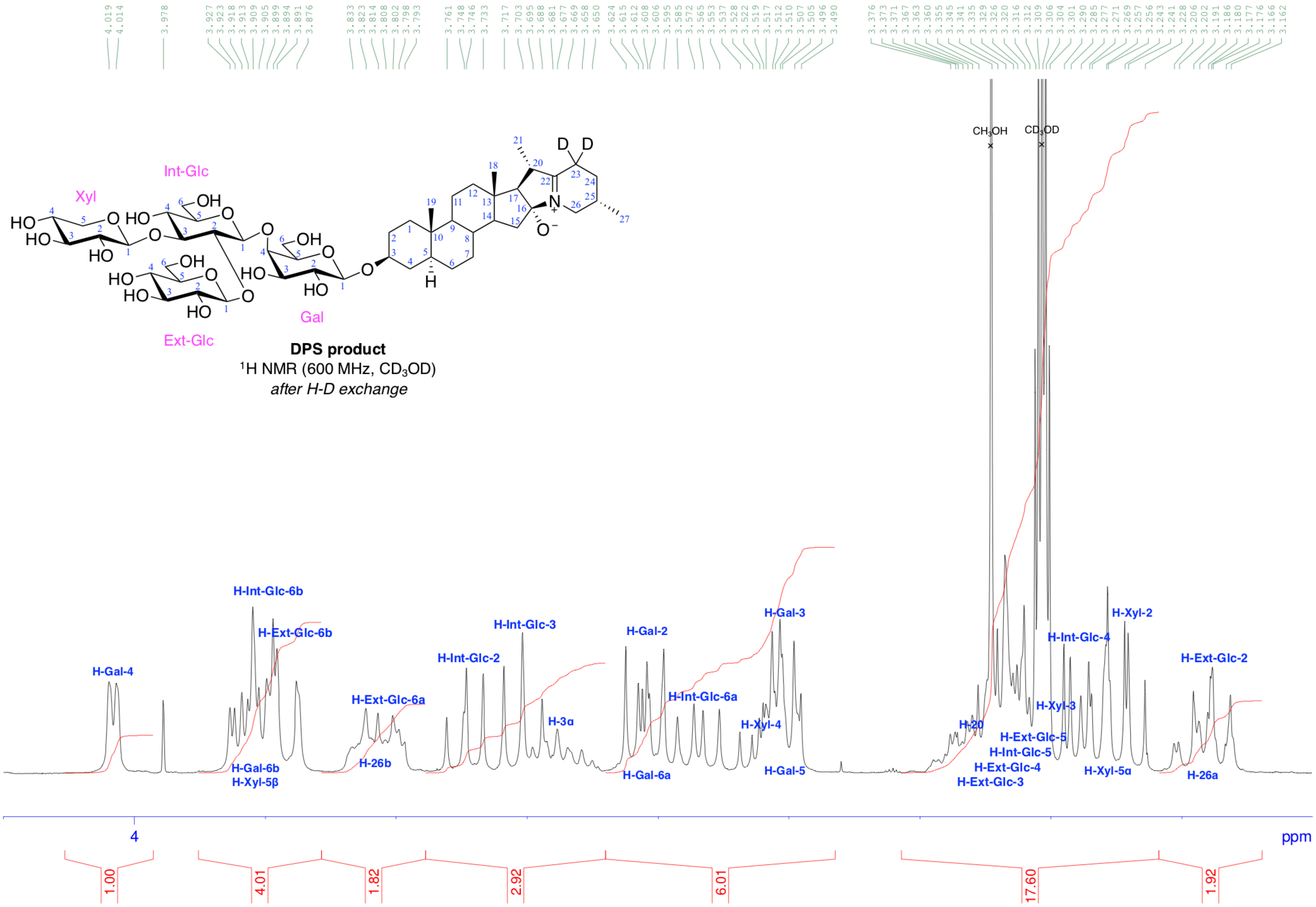

DPS product

^{1}H NMR (600 MHz, CD_3OD)

after H-D exchange

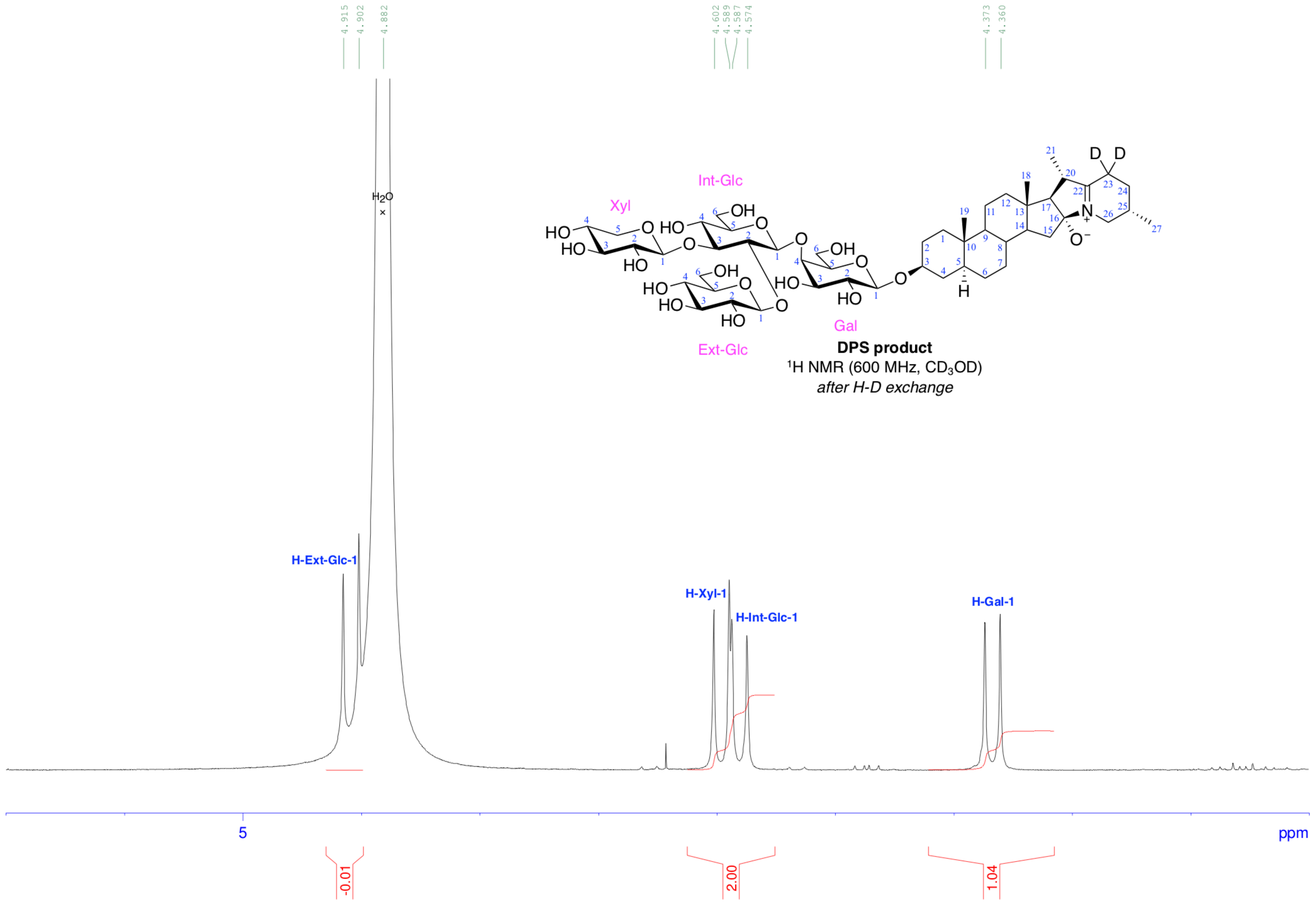

4.915
4.902
4.882
4.602
4.589
4.587
4.574
4.373
4.360
Int-Glc
Xyl
Ext-Glc
Gal
DPS product
^{1}H NMR (600 MHz, CD_3OD)
after H-D exchange
H_2O
H-Ext-Glc-1
H-Xyl-1
H-Int-Glc-1
H-Gal-1
5
ppm
-0.01
2.00
1.04

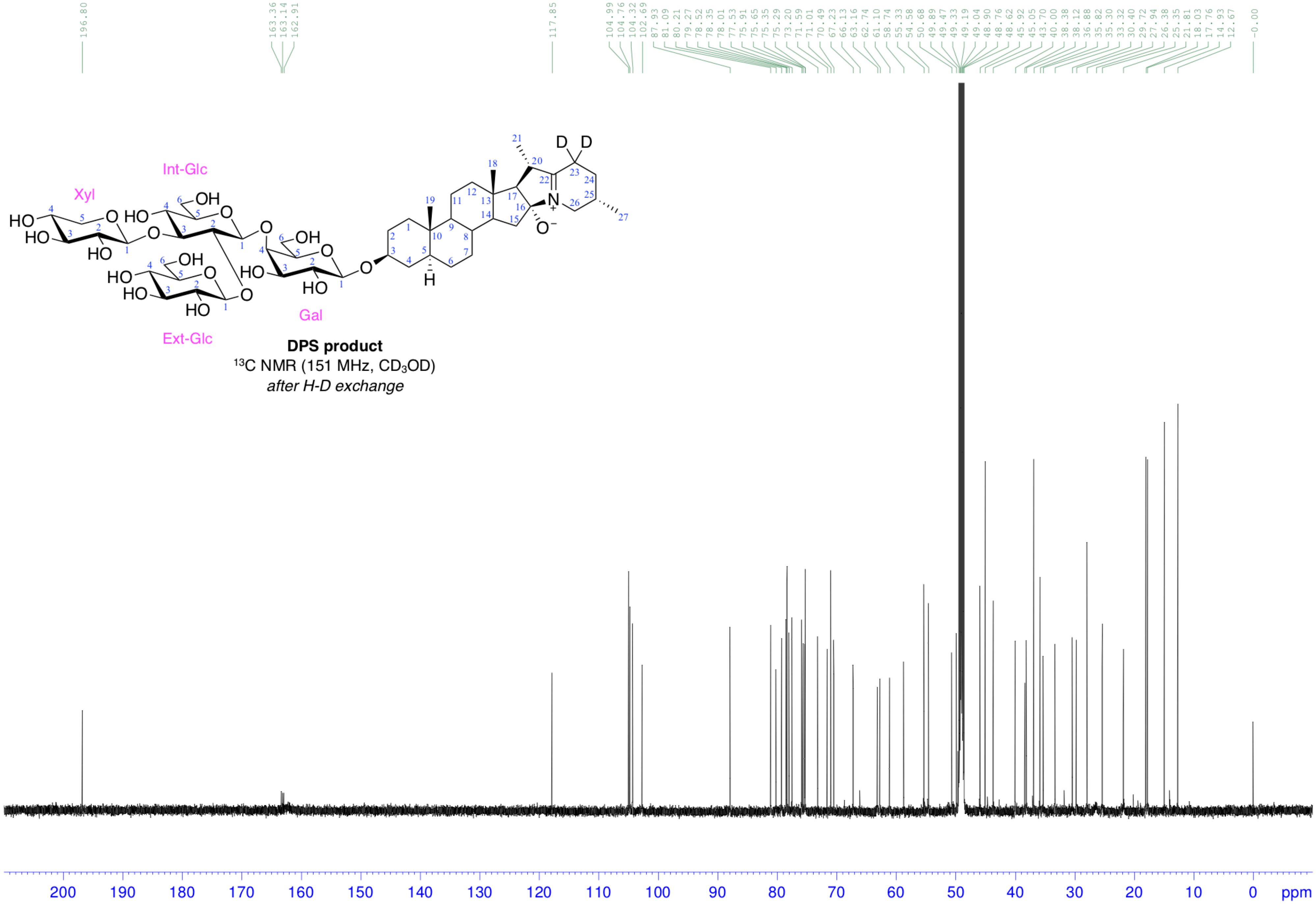
196.80
163.36
163.14
162.91
117.85
104.99
104.76
104.32
102.69
87.93
81.09
80.21
79.27
78.52
78.35
78.01
77.53
75.91
75.65
75.35
75.29
73.20
71.59
71.01
70.49
67.23
66.13
63.16
62.74
61.10
58.74
55.33
54.58
50.68
49.89
49.47
49.33
49.19
49.04
48.90
48.76
48.62
45.92
45.05
43.70
40.00
38.38
38.12
36.88
35.82
35.30
33.32
30.40
29.72
27.94
26.38
25.35
21.81
18.03
17.76
14.93
12.67
-0.00
Xyl
Int-Glc
Ext-Glc
Gal
DPS product
13C NMR (151 MHz, CD3OD)
after H-D exchange
ppm

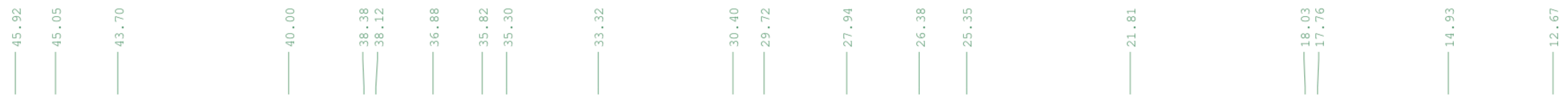

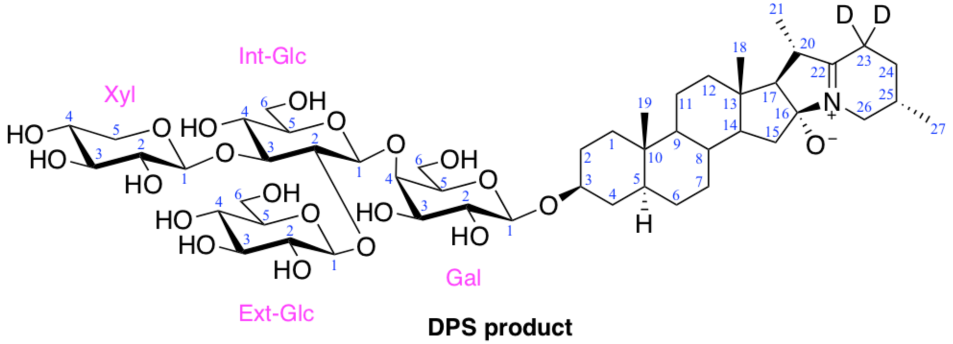

DPS product

^{13}C NMR (151 MHz, CD_3OD)

after H-D exchange

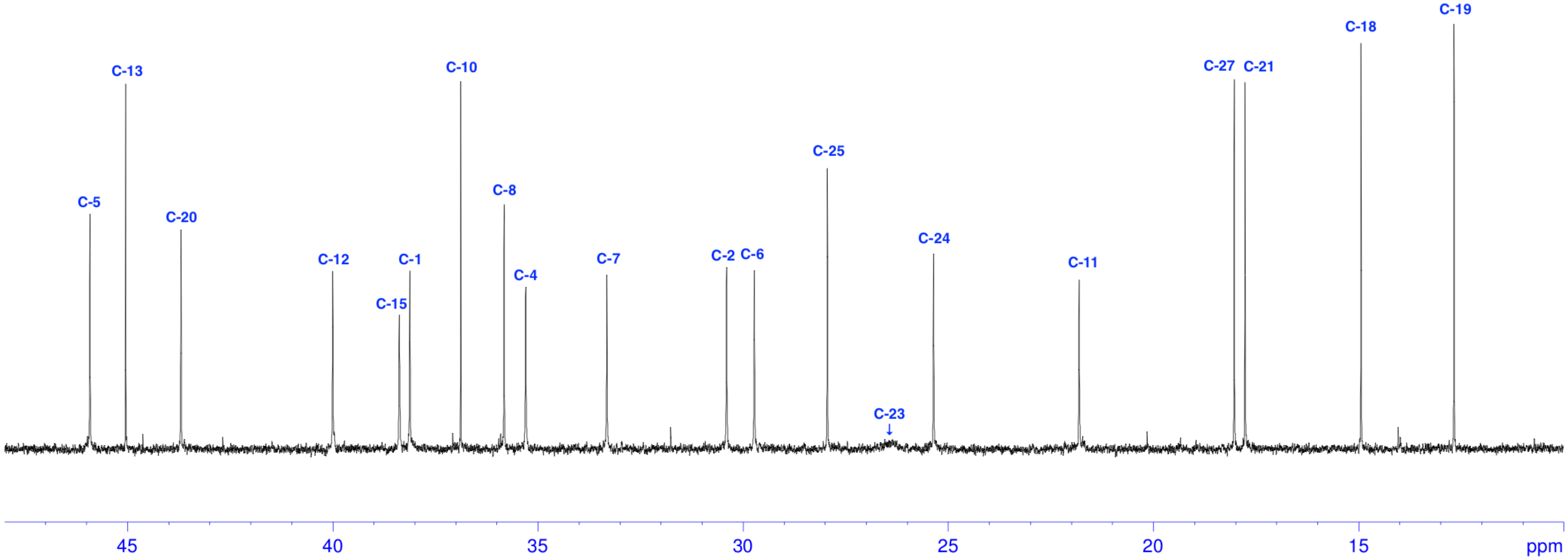

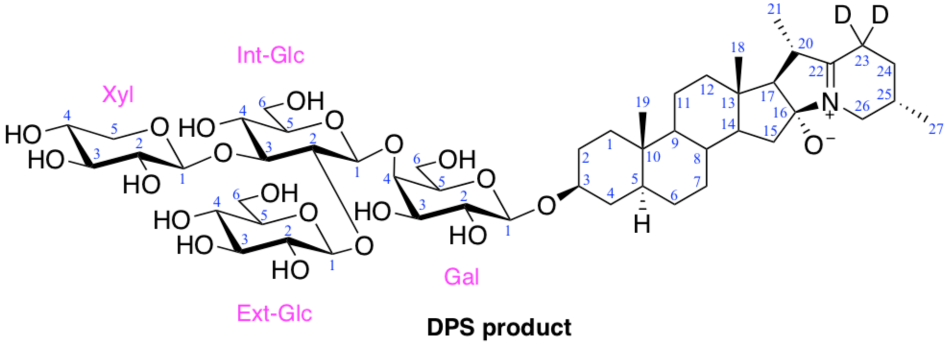

DPS product

^{13}C NMR (151 MHz, CD_3OD)

after H-D exchange

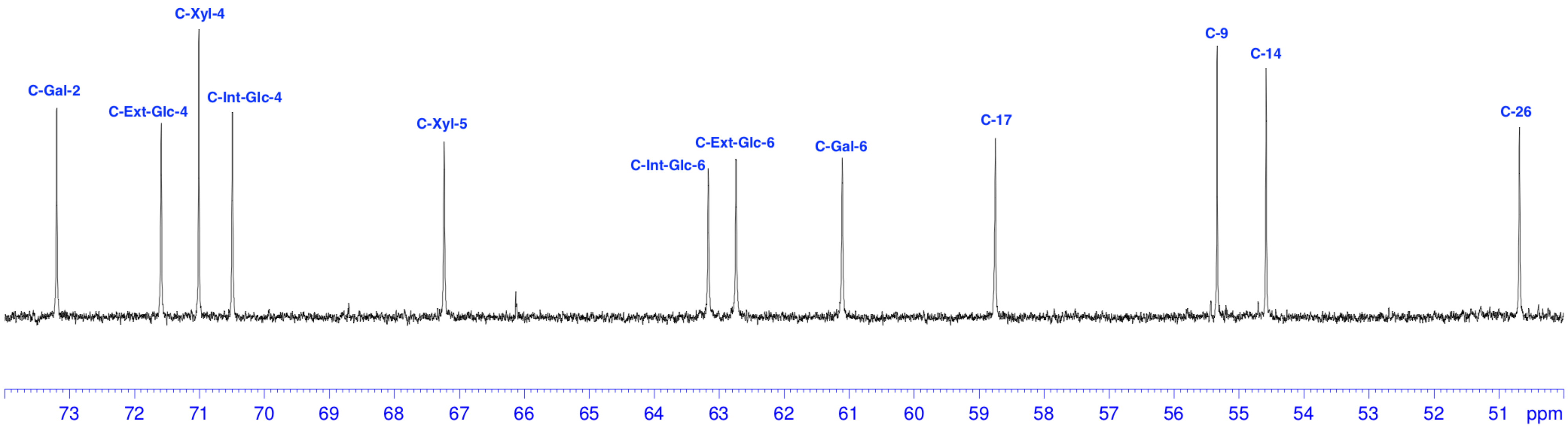

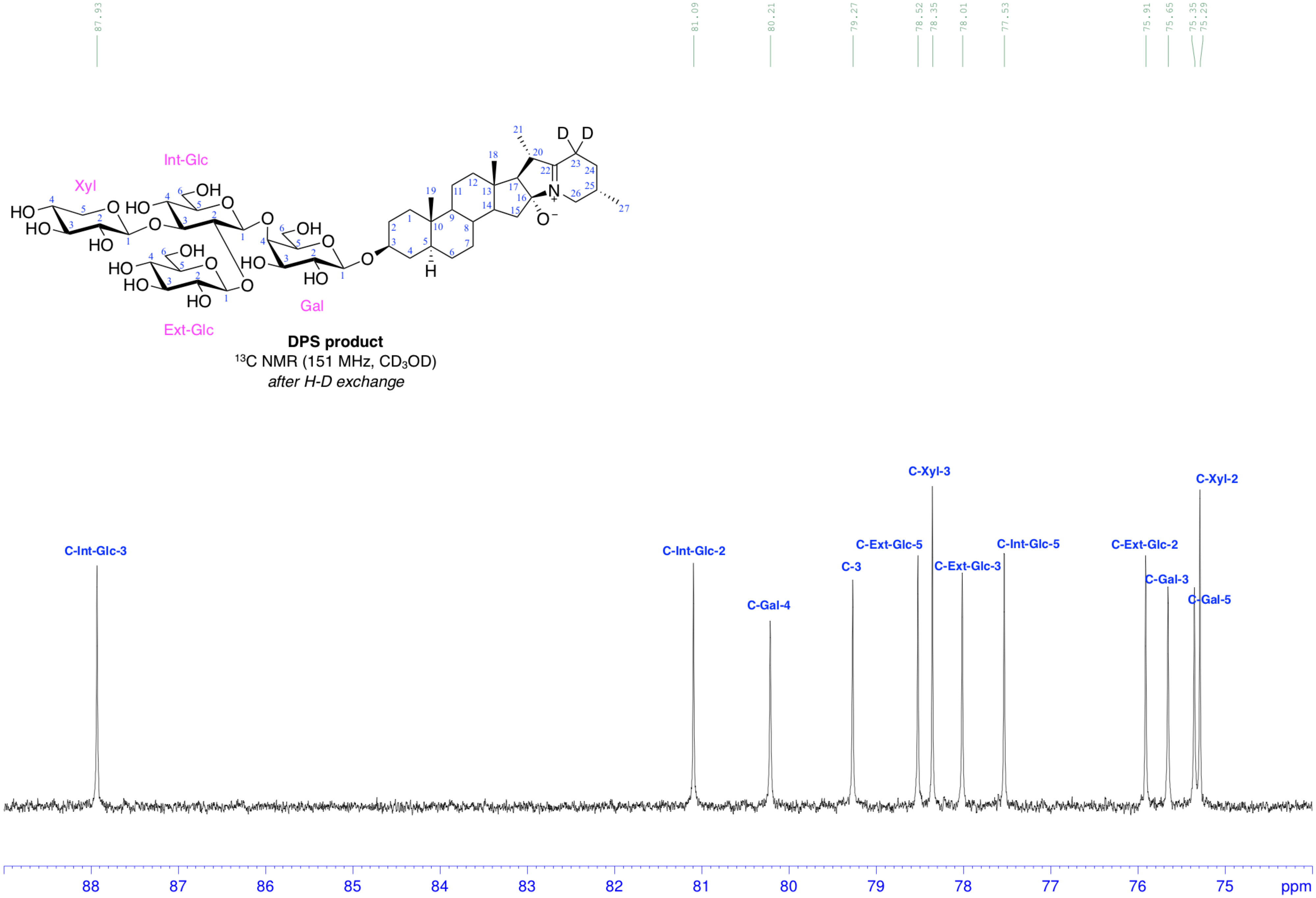

DPS product

^{13}C NMR (151 MHz, CD_3OD)

after H-D exchange

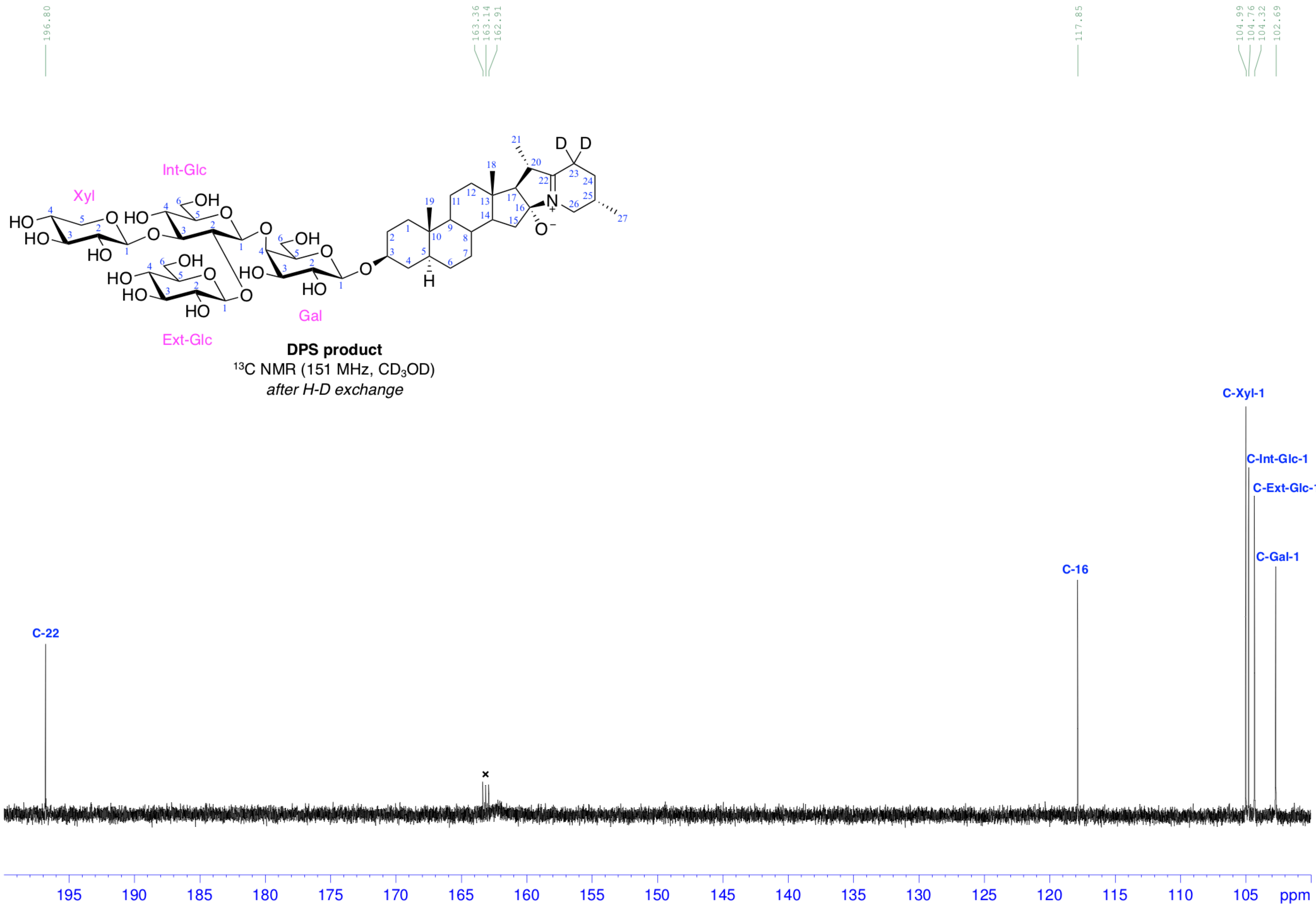

DPS product

^{13}C NMR (151 MHz, CD_3OD)

after H-D exchange

DPS product
COSY (CD_3OD)
after H-D exchange

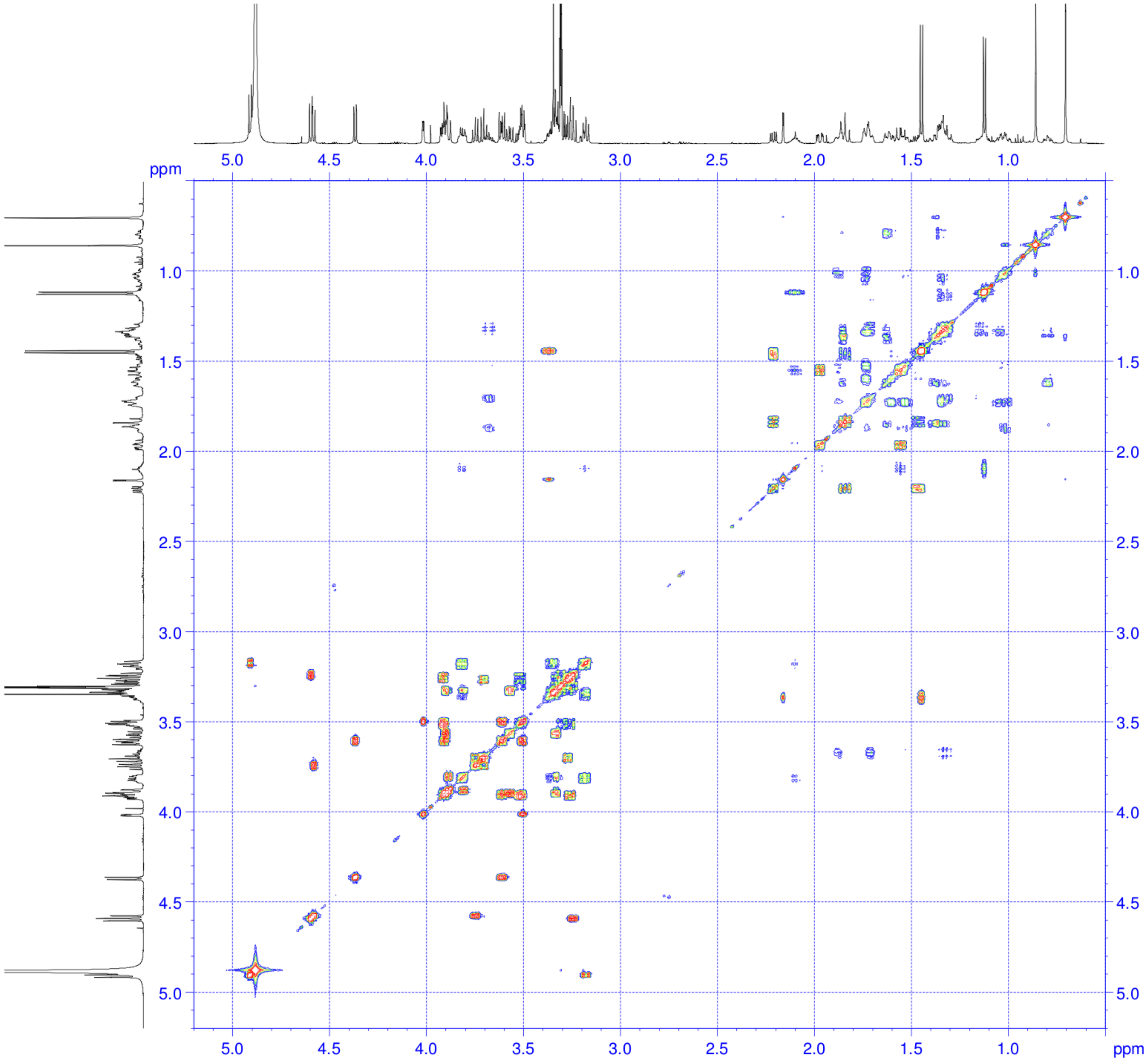

DPS product

NOESY (CD_3OD)

after H-D exchange

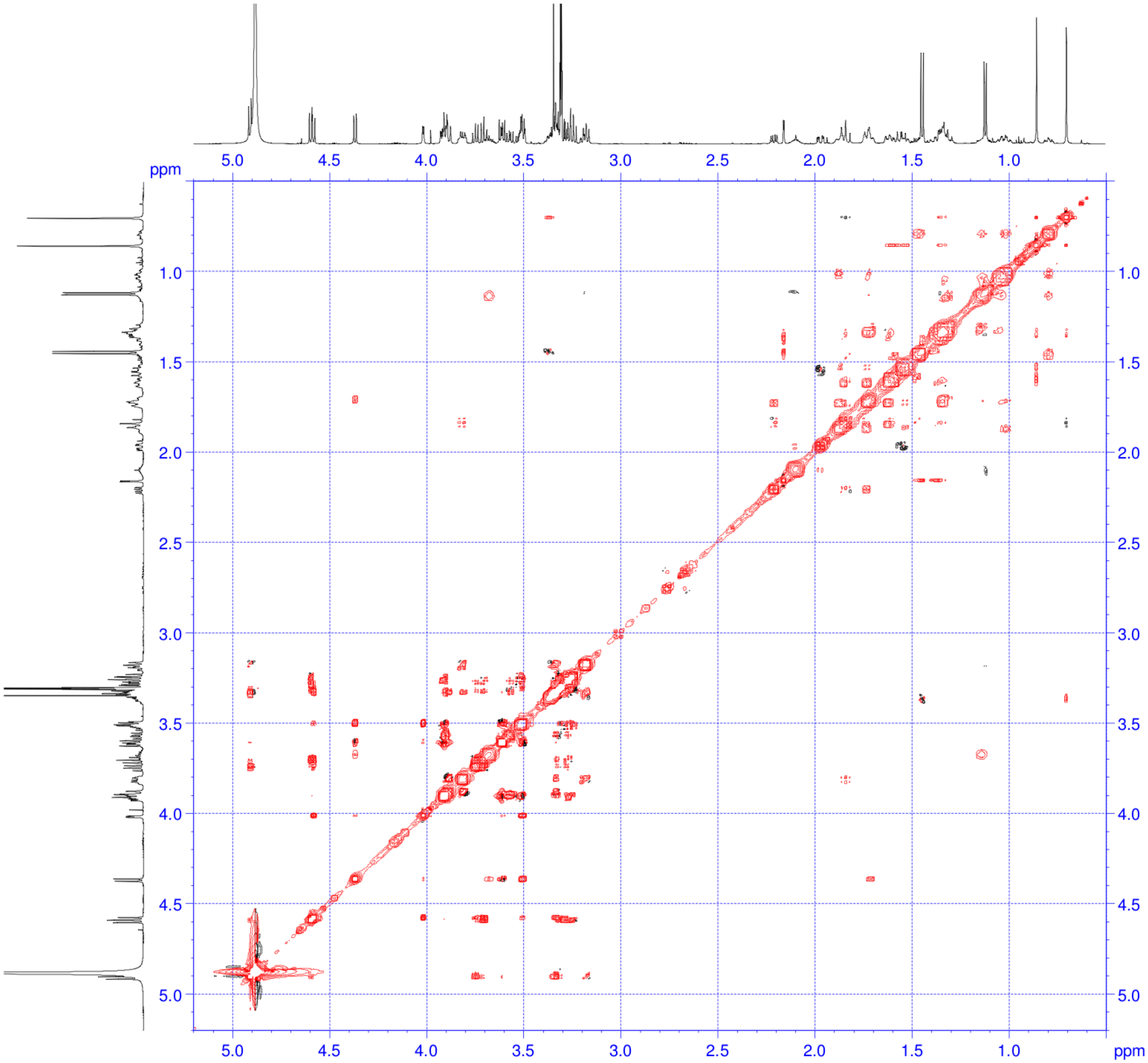

DPS product
HSQC (CD_3OD)
after H-D exchange

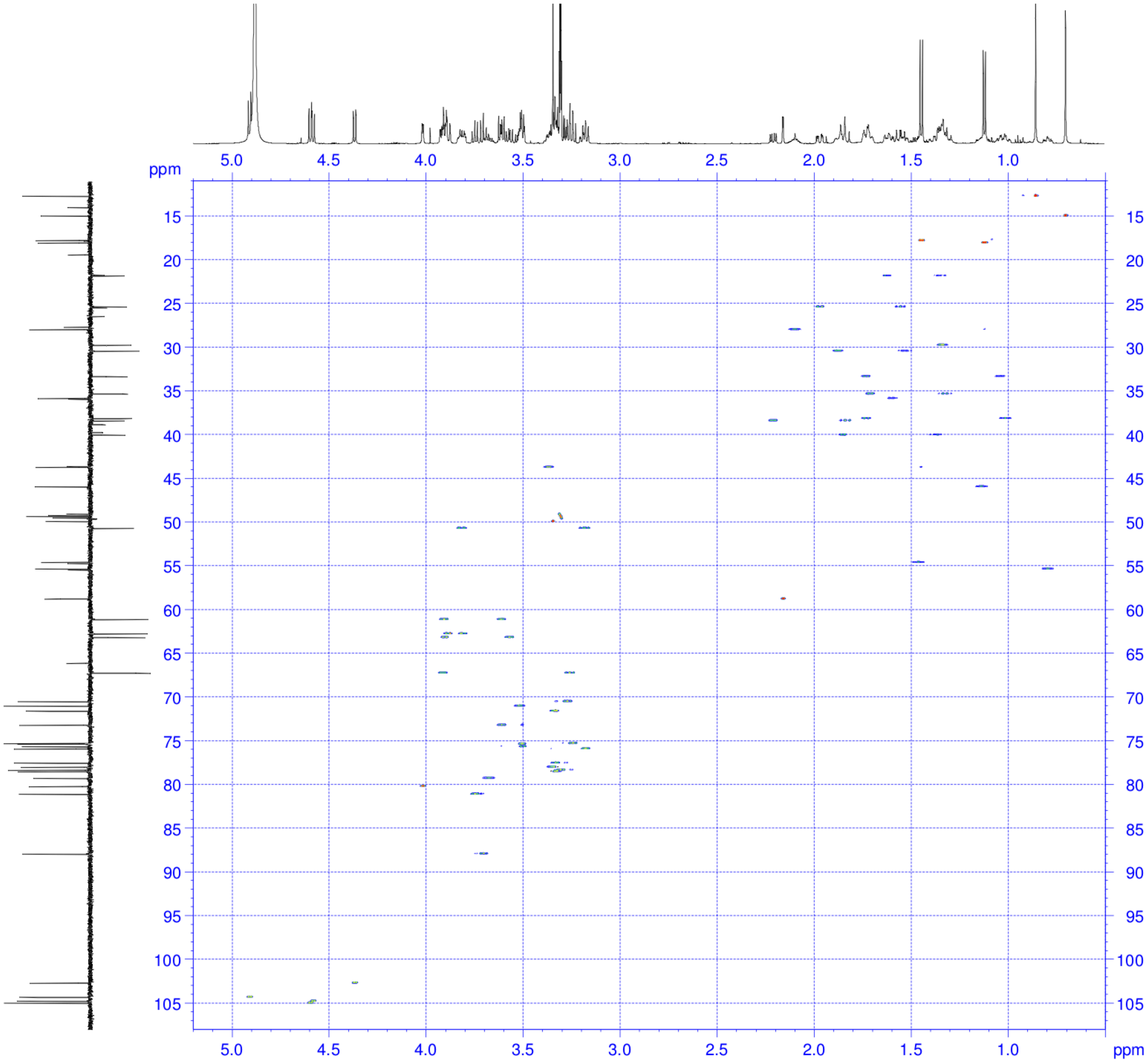

DPS product
HMBC (CD_3OD)
after H-D exchange

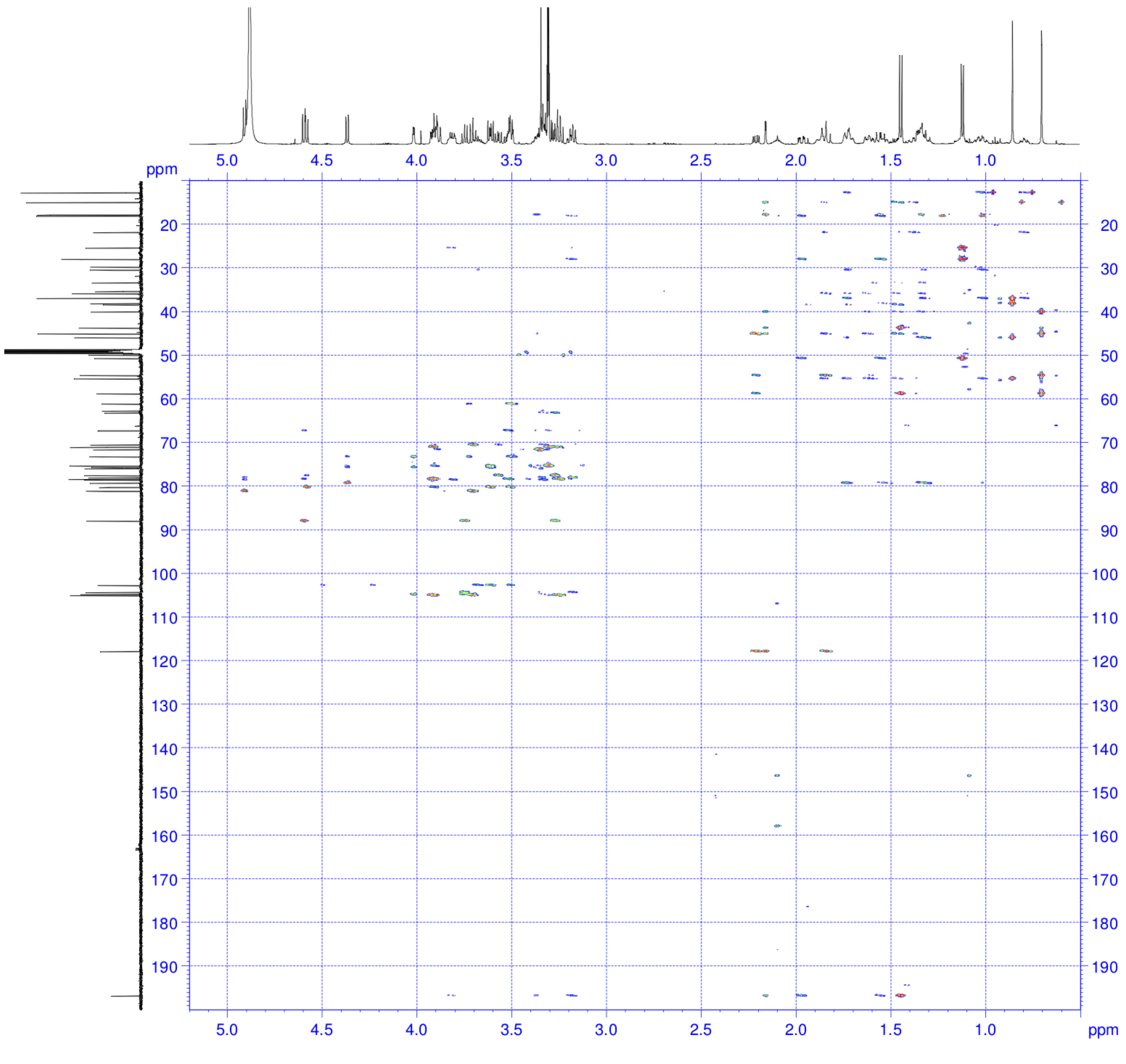

Supplement: Supplementary file 3 — Supplementary Data 1 [file 41467_2021_21546_MOESM3_ESM.pdf]
